# The architecture of mammalian ribosomal protein promoters

**DOI:** 10.1186/1471-2148-5-15

**Published:** 2005-02-13

**Authors:** Robert P Perry

**Affiliations:** 1 Fox Chase Cancer Center Philadelphia, PA 19111 USA

## Abstract

**Background:**

Mammalian ribosomes contain 79 different proteins encoded by widely scattered single copy genes. Coordinate expression of these genes at transcriptional and post-transcriptional levels is required to ensure a roughly equimolar accumulation of ribosomal proteins. To date, detailed studies of only a very few ribosomal protein (rp) promoters have been made. To elucidate the general features of rp promoter architecture, I made a detailed sequence comparison of the promoter regions of the entire set of orthologous human and mouse rp genes.

**Results:**

A striking evolutionarily conserved feature of most rp genes is the separation by an intron of the sequences involved in transcriptional and translational regulation from the sequences with protein encoding function. Another conserved feature is the polypyrimidine initiator, which conforms to the consensus (Y)_2_C^+1^TY(T)_2_(Y)_3_. At least 60 % of the rp promoters contain a largely conserved TATA box or A/T-rich motif, which should theoretically have TBP-binding capability. A remarkably high proportion of the promoters contain conserved binding sites for transcription factors that were previously implicated in rp gene expression, namely upstream GABP and Sp1 sites and downstream YY1 sites. Over 80 % of human and mouse rp genes contain a transposable element residue within 900 bp of 5' flanking sequence; very little sequence identity between human and mouse orthologues was evident more than 200 bp upstream of the transcriptional start point.

**Conclusions:**

This analysis has provided some valuable insights into the general architecture of mammalian rp promoters and has identified parameters that might coordinately regulate the transcriptional activity of certain subsets of rp genes.

## Background

Ribosomes are vital organelles, which catalyze protein synthesis in all living organisms. Eukaryotic ribosomes consist of four RNA molecules (rRNAs) and 79 different proteins. The mammalian genes encoding the rRNAs are multicopy and clustered at a few loci, whereas those encoding the ribosomal proteins (rp genes) are single copy and scattered throughout the genome [1]. In addition to the functional rp genes, all of which contain introns, mammalian genomes contain many nonfunctional intronless rp pseudogenes [2]. The earliest determinations of mouse rp gene sequences and of transcriptional start points (tsp's) revealed a salient feature of rp genes, namely that transcription is initiated at a C residue within a polypyrimidine tract [3-5]. A recent study by Kenmochi and coworkers [6] has demonstrated that this is a general property of virtually all human rp genes. Because of this novel feature, the rp mRNAs contain a 5' terminal oligopyrimidine sequence (TOP), which is essential for their translational control [7].

Coordinated expression of the rp genes at transcriptional and post-transcriptional levels is required to ensure a roughly equimolar accumulation of ribosomal proteins. Transcriptional run-on measurements with nuclei of rapidly proliferating cells indicated equivalent loading of RNA polymerases on three unlinked mouse rp genes [8], consistent with the equal abundance of the corresponding rp mRNAs [9]. Moreover, the promoters of these genes were of comparable strength in driving the expression of a common reporter gene [8]. These results suggested that similar promoter strength and mRNA processing efficiency might provide a basis for the coordinated expression of rp genes. However, whether this concept applies to all rp genes or to distinctive subsets of genes is presently unclear.

Despite the obvious importance of rp gene expression for cell viability, there have been very few experimental studies of rp promoter architecture and transcriptional regulation in higher eukaryotes. To date, efforts to identify functionally relevant cis-acting regulatory elements and transcription factor binding sites have been made for only 9 mammalian [8,10-23] and 2 amphibian [24,25] rp genes, less than 15 % of the total rp gene complement. These studies identified regulatory elements in the promoter-proximal regions, both upstream and downstream of the tsp. Some of these elements contained binding sites for known transcription factors, notably GABP, Sp1 and YY1. When the binding of any individual factor was eliminated by a site-specific mutation, transcriptional activity was reduced, but not abolished, indicating that the overall transcription efficiency is determined by a combinatorial effect of multiple factors. No regulatory element common to all rp genes was found, although certain elements were present in several of the genes that were studied. None of these rp genes contained a canonical TATA box in the -25 to -30 region, although some had a "TATA-like" A/T-rich sequence, which might bind the general transcription factor TBP under certain circumstances.

Because the previous experimental studies were limited to a small subset of rp genes, it is important to know whether the results of those studies can be used to predict special features of promoter architecture that characterize the entire set of rp genes or that could be used to sort the rp genes into classes with similar features. This applies not only to transcription factor-binding sites, but also to the polypyrimidine tract that spans the tsp. As an initiator element, this tract is atypical because the tsp of initiators is normally an adenine residue, flanked on both sides by pyrimidines [26].

To address these issues, I have applied the principle of "phylogenetic footprinting", which holds that important regulatory sequences will have a tendency to be evolutionarily conserved and thus revealed by a sequence comparison of corresponding regions of orthologous genes [27]. Some regulatory promoter elements may escape detection by this approach, but a substantial majority will probably be recognized with confidence [28]. Although previously done on a small scale for a few selected rp genes [19,21,29-32], such an analysis has not heretofore been made for the entire rp gene population. I have therefore compared the promoter regions of all 79 orthologous human and mouse rp genes and have extended the comparisons to include chicken, amphibian and fish counterparts when these sequences were available. I have compared the sequence organization of the rp promoters with that of the promoters of non-rp TOP genes and other genes in the housekeeping category. This analysis has provided some useful insights into the general architecture of mammalian rp promoters and has identified parameters that might coordinately regulate the transcriptional activity of certain subsets of rp genes.

## Results

### Compilation of the promoter sequences of orthologous rp genes

To make a study of the evolutionarily conserved features of ribosomal protein gene promoters, I first had to assemble a set of orthologous human and mouse rp gene sequences, in which the transcription start sites are reasonably well defined. For all of the human rp genes, annotated sequences are now available owing to the studies of Kato, Kenmochi and coworkers [6,33], who systematically determined the 5' termini of human rp mRNAs by the oligo-capping method. While these studies indicated some small variations in the exact 5' ends of individual rp mRNAs, the tsp's could usually be specified to within a few nucleotides. Independent determinations of the human tsp's by the oligo-capping procedure are also available on the database of transcription start sites (DBTSS) compiled by Sugano and colleagues [34]. The agreement between the two sets of oligo-capping data and the results of primer extension/nuclease protection (PE/S1) experiments is generally very good [see Additional file 1]. In all cases, the human tsp's selected in the present study correspond either to the most abundant oligo-capped cDNA or to an observed variant.

In contrast to the situation with the human rp genes, accurate determinations of the tsp's of mouse rp genes have been made in only a few cases, and therefore the annotations of rp genes in the mouse genome databases are generally incomplete. To overcome this problem, I used the mVISTA alignment program [35] to compare mouse sequences extending several kb upstream of the coding regions with the sequences of the corresponding human orthologues. Fortunately, the strong conservation of sequences in the proximal promoter regions and in the first exons enabled me to identify the probable tsp's of most mouse rp genes with confidence. For the most part, the tsp's that were predicted by this strategy corresponded exactly to those previously determined by primer extension and/or nuclease protection assays of murine rp genes [see Additional file 1]. Some information on putative tsp's of mouse rp genes is available on the DBTSS database. However, the most prominent of these tsp's did not agree with the PE/S1 data. Moreover, they were not coincident with the aligned C tsp's of the human orthologues, but were frequently at adjacent or nearby T residues. Conceivably, these discrepancies are caused by a technical bias, as is sometimes encountered in the generation of genome-wide full-length cDNA libraries [36]. Therefore, relying on maximum sequence conservation and the best agreement with previous experimental data, I compiled aligned sequences of all 79 pairs of orthologous human and mouse rp genes, and used these sequences for further analysis of the promoter regions.

In a preliminary analysis, I used the mVISTA sequence alignment program to examine six to ten kb of 5' flanking sequence of three pairs of orthologus rp genes (*rpS16*, *rpL30 *and *rpL32 *). These genes were selected because the mouse orthologues have been previously studied extensively in my laboratory. Scans with a 50 bp window indicated that the conserved (> 75 % identity) flanking sequences are largely confined to regions within a few hundred base pairs of the tsp. There was very little alignment of the upstream region sequences and many gaps caused by the presence of numerous insertion elements. With this analysis, I detected only one short block of conserved sequence at -2 kb in *rpL30 *(77 % identity over 84 bp), one short block at -1.5 kb in *rpL32 *(82 % identity over 60 bp), and none in *rpS16 *. Neither of the conserved blocks contained any recognizable transcription factor binding sites or other remarkable characteristics. The lack of any credible long-range regulatory elements is consistent with earlier conclusions based on transient transfection experiments with these genes [8,17,37]. Given these results, I restricted the analysis of the full set of rp genes to the transcribed portions of the genes and about one kilobase of 5' flanking sequence [see Additional file 2].

**Figure 1:**
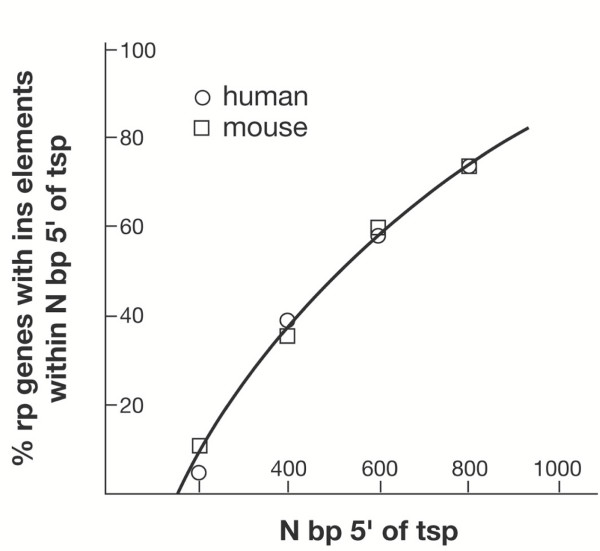
**The location of insertion elements in the 5'-flanking regions of rp genes**. Insertion (ins) elements were identified by the RepeatMasker program in scans of up to one kilobase of sequence 5' of the tsp, and the location of the element nearest to the tsp recorded for 79 human and mouse rp genes [see Additional file 2]. The distance from the tsp was divided into 200 bp intervals, the percentage of rp genes with an element within each interval determined, and the values plotted cumulatively against the distance from the tsp.

An initial analysis of the large data set revealed the presence of repetitive sequence insertion elements (sines for human and B1/B2 for mouse) in the 5' flanks of a very high proportion of the rp genes [see Additional file 2]. Indeed, as seen in Figure 1, half of the rp genes contain such an element within 500 bp of the tsp and over 80 % contain elements within 900 bp. The distribution is very similar in the human and mouse rp genes. Both ancestral elements, which are moderately conserved between mouse and human, and lineage-specific elements could be identified. As in the case of the three examples discussed above, very little sequence conservation was evident more than 200 bp upstream of the tsp. Accordingly, for the refined analysis of rp promoter architecture, I analyzed segments extending from 200 bp upstream to 100 bp downstream of the tsp. These segments were scrutinized for the quality of TATA box motifs in the -25 region, for conserved sequences in the initiator region, for transcription factor binding sites, and for the location of the AUG translation start codons.

### Criteria for the annotation of rp promoter sequences

**Figure 2:**
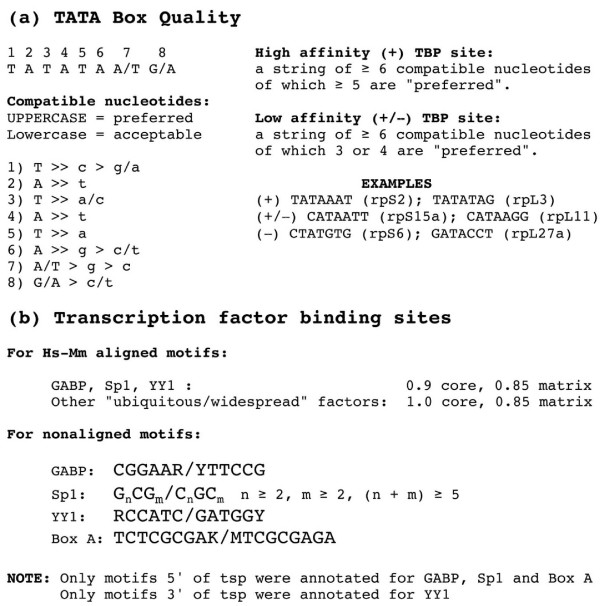
**Criteria for rp promoter annotation**. (a). Quality of the TATA box for TBP binding based on a structural analysis of TBP-DNA complexes. Rules adopted for + and +/- ranking and some examples in rp promoters are shown at the right. (b). Criteria used for identification of potential transcription factor-binding sites by the rVISTA program for motifs aligned in human-mouse promoter sequence comparisons and with the FindPatterns program for unaligned motifs.

To evaluate the quality of TATA box motifs, I established criteria based on rules derived from a crystallographic analysis of TBP-DNA complexes [38]. With these rules one can classify each nucleotide in the motif as being "preferred" or "acceptable" or "incompatible" with TBP binding (Figure 2a). I considered motifs with a string of 6 or more compatible nucleotides, of which at least 5 are "preferred", as being capable of binding TBP with high affinity (+ motifs). Those with a string of 6 compatible nucleotides, 3 or 4 of which are "preferred", were considered as possible low affinity sites (+/- motifs). Sequences that do not satisfy these criteria were judged to be incapable of unaided binding to DNA (- motifs).

Employing stringent criteria (Figure 2b), I used the rVISTA program [39] to search systematically for conserved (aligned) sites that would be predicted to bind the three transcription factors (GABP, Sp1, and YY1) that were identified in earlier experimental studies of rp promoters and also for highly conserved sites that might bind other ubiquitously expressed factors. In addition, I scanned both the human and mouse rp promoter sequences for unaligned optimal sites for the above-mentioned factors and for a motif termed Box A, which was previously implicated in rp gene expression [19]. I used the results of these analyses to complete the annotation of all 79 pairs of aligned human and mouse rp promoter sequences, which can be viewed individually on pages 1 through 80 of a supplementary file [see Additional file 3].

### Annotated comparisons of human/mouse promoter sequences

**Figure 3:**
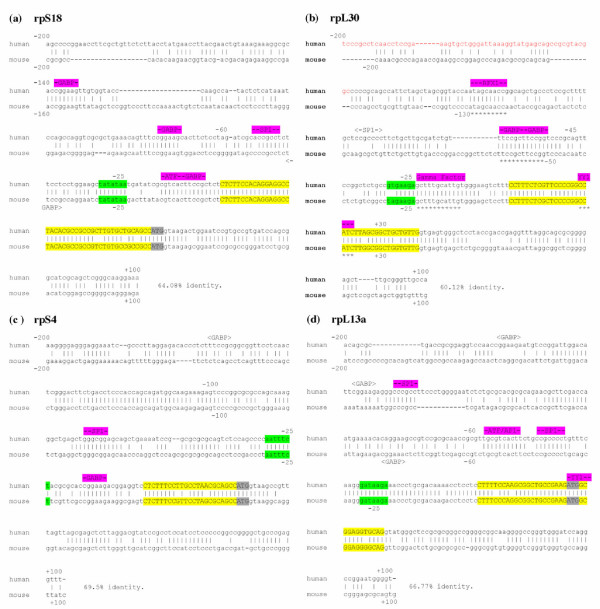
**Examples of comparisons of human and mouse rp promoter sequences from -200 to +100**. Exon I is in uppercase letters, highlighted in yellow with the ATG translation initiation codon, if present, highlighted in gray. The sequences evaluated for TATA box quality are highlighted in green. Putative transcription factor-binding sites in aligned sequences are shown above the sequences, highlighted in fuchsia; sites in unaligned sequences are shown above the human or below the mouse sequences and enclosed in carets. An upstream sequence identified as an insertion element is in red letters. Gaps inserted by the alignment program to maximize overall sequence identity are indicated by dashes. (a) *rpS18 *, (b) *rpL30 *, (c) *rpS4 *, (d) *rpL13a *. In *rpL30 *the elements with known functional relevance are underlined with asterisks.

Illustrative examples of four annotated promoter sequence comparisons are shown in Figure 3. All four of these examples illustrate the strong sequence conservation of the non coding portions of exon 1, which is a general feature of the entire rp gene family [see Additional file 3]. The AUG translation initiation codon is located within exon 1 of *rpL13a *(panel d) and within exon 2 of *rpL30 *(panel b), whereas in *rpS18 *(panel a) and *rpS4 *(panel c), it is at the extreme 3' end of exon 1. Many rp genes have this latter feature, which may be relevant to the evolution of vertebrate rp promoters (see below).

The *rpS18 *promoter contains a conserved TATA (+) motif, while the TATA motifs of *rpS4 *and *rpL13a *were scored as (+/-) and that of *rpL30 *as (-). Well-conserved (aligned) consensus binding sites for GABP and/or Sp1 are located upstream of the tsp in all four examples; downstream YY1 sites are evident in *rpL30 *and *rpL13a *. The *rpS18 *and *rpL13a *promoters also contain conserved consensus binding sites for the ubiquitous AP1/ATF factors. The promoter elements that were found to have functional significance in experimental studies of the mouse *rpL30 *gene [11] are all conserved in the human orthologue (panel b). This includes sites for RFX1 and the Gamma Factor as well as the GABP and YY1 sites. In addition to the conserved sites, a few non conserved (unaligned) GABP and Sp1 sites of uncertain relevance are evident in *rpL30 *and *rpL13a *.

The results of this type of analysis for the full set of rp promoters are tabulated in Table 1. The identity between human and mouse sequences in the 300 bp segment (-200 to +100) that was analyzed ranged from 50 to 75 % with an average of 61 %. The location of the translation initiation codon was conserved in all except one rp gene (*rpL29 *). In 29 % of the rp genes the AUG codon is at the extreme 3' end of exon 1 and in 47 % it is in exon 2. Thus, in 3/4 of the rp genes, the genetic elements involved in transcriptional/translational regulation are spatially separated from those with protein encoding function.

**Table 1:** Ribosomal protein promoters Quality of the TATA Box Motifs, the Number of Transcription Factor-Binding Sites and the Location of AUG Codons.

**RP GENE**	**TATA Quality**		**% H/M identity in 300 bp ‡**	**GABP (5' of tsp)**		**Sp1 (5' of tsp)**		**YY1 (3' of tsp)**		**OTHER (5' of tsp)**	**AUG †**
	**Hs**	**Mm**		**H-M**	**H**	**H-M**	**H**	**H-M**	**H**		
SA	+	+	58		1		2	**1**		**ATF, CREB**	E2
S2	+	+	54			**1**	1				E2
S3	+	+	60	**1**		**1**		**1**			E1
S3a	–	–	75	**2**		**1**				**AP1**	E1
S4	+/–	+/–	70	**1**	1	**1**					E1e
S5	+/–	+	62	**1**							E2
S6	–	–	56	3 *			1		1	Box A *	E1
S7	+/–	+	56		4		2			**Box A**	E2
S8	+/–	+/–	60			**1**					E1e
S9	+	+	50		1			**1**		**ATF, CREB, AP1**	E2
S10	–	–	64	**1**		**1**	1	**1**		**Nrf1**	E2
S11	+/–	+/–	53	**1**		**1**	1	**1**		**Box A, ATF, CREB, AP1**	E1
S12	+	+	62	**1**						**Nrf1**	E2
S13	–	+/–	61			**1**		**1**		**AP1**	E1
S14	–	–	62	3 *		**1**				**AP1**	E2
S15	+/–	+/–	52	**1**		**1**		**1**		**Nrf1**	E1e
S15a	+/–	+/–	59	**1**				**2**	1	**Box A**	E2
S16	–	+/–	74			2 *	2			**Nrf1**	E1
S17	+/–	+	60	**1**	1		2		1		E1e
S18	+	+	64	**3**		**1**				**ATF, CREB**	E1e
S19	+/–	+/–	72			**1**	1			**Box A (2)**	E2
S20	+	+	63	**1**		**1**					E1e
S21	+	+/–	52	**3**			1				E2
S23	–	–	65	**1**		**1**		**1**			E1e
S24	+	+	59		1		1	**2**			E1e
S25	–	–	56	**1**	1						E1e
S26	+	+	53					**1**			E1e
S27	+	+	71	**2**	**1**			**1**			E1
S27a	–	–	57			**1**	1	**1**			E2
S28	+	+	67	**1**	1		1	**1**		**AP1**	E1
S29	–	–	inc								E1
S30	–	–	68			**4**		**1**		**ATF, CREB**	E2
L3	+	+	56		2	**1**		**1**	1		E1e
L4	+	+	68				2				E1e
L5	+	+	62				1	**1**			E1e
L6	+/–	+/–	62	**1**	1			**1**			E2
L7	+/–	+/–	69	**1**				**1**		**Nrf1**	E1
L7a	+	+	51			**2**	1	**1**	1	Box A*, B	E1e
L8	+/–	+/–	50	**1**	1	**1**	1	**1**		**AP1**	E2
L9	–	–	57						1	**ATF, CREB (2)**	E2
L10	+	+	60				1				E2
L10a	+	+	55							**Box A, Nrf1**	E1
L11	+/–	+/–	64	**1**		**1**		**1**			E2
L12	+	+	73		1					**Box A**	E1
L13	–	+/–	55				1	**1**			E2
L13a	+/–	+/–	67		2	**2**		**1**		**ATF, CREB, AP1**	E1
L14	+/–	+/–	58			**2**	1			**Nrf1**	E1e
L15	–	–	66	**1**		**1**	1	**2**			E2
L17	–	–	69		1			**1**	1	**Box A**	E2
L18	–	–	51			**1**		**2**		**ATF, CREB, AP1**	E1e
L18a	–	–	67	**1**		**1**					E1
L19	+/–	+/–	65	**1**	1						E1
L21	–	–	62			**1**		**1**			E2
L22	–	–	61								E1
L23	+	+	64		1	**1**			1		E1
L23a	+/–	+/–	63					**1**		**Box A**	E1
L24	+/–	+/–	56		1			**1**			E1
L26	+/–	+/–	64	**1**	1			**2**		**Box A**	E2
L27	–	–	66	**3**		**1**		**1**			E2
L27a	–	–	56	**1**		**1**	1			**Box A**	E1e
L28	+	+/–	56	**2**	1		1	**1**			E2
L29	+/–	+/–	68	**2**			1		1		E1e//2
L30	–	–	60	2 *			1	1 *		RFX1* Gamma*	E2
L31	–	–	62	**1**		**1**					E2
L32	–	–	68	1 *				1 *	1 *	Gamma *	E2
L34	–	–	55	**1**		**1**	2	**1**			E2
L35	–	–	59			**2**	4	**1**			E1e
L35a	–	–	64			**1**	1	**1**			E2
L36	+/–	+	51				1		1		E2
L36a	+	+	62	**1**		**2**	2	**1**			E1e
L37	+	–	60	**2**	2	**1**	1	**2**			E1e
L37a	+/–	+/–	52	**1**						**Nrf1**	E1e
L38	+/–	–	62	**2**							E2
L39	–	+/–	61	**1**	1		1	**1**			E1e
L40	–	–	62	**2**			3			**ATF, CREB**	E2
L41	+	+	54	**1**	1	**1**				**ATF, CREB, AP1**	E2
LP0	+	+	59		1						E2
LP1	+/–	+/–	62	**1**							E1
LP2	+/–	–	55			**1**					E2

Bolded numbers indicate that the sites are aligned in orthologous human and mouse genes. An asterisk indicates that there is experimental evidence for the functionality of one or more of the sites.

‡ From –200 to +100 relative to the tsp.

† E1, within Exon 1; E1e, at the extreme 3' end of Exon 1; E2, within Exon 2. The locations are conserved in all rp genes except *rpL29 *.

Contrary to previous impressions based on an incomplete set of rp genes, the rp promoters cannot generally be classified as "TATA-less". Thirty-five percent of the promoters contain, in the -25 region, a TATA box that should theoretically bind TBP with high affinity. An additional 25 % have an A/T-rich tract in this region, which might bind TBP with lower affinity, and the remainder would not be predicted to bind TBP without help from other proteins. For the most part, the TATA box quality is well conserved between the two mammalian species: the assessed quality of human and mouse TATA motifs was the same in 82 % of the rp promoters.

The prevalence in rp promoters of evolutionarily conserved GABP, Sp1 and YY1 binding sites is readily apparent from these results. Conserved upstream GABP and Sp1 sites are present in 54 % and 48 % of the rp promoters, respectively. Conserved downstream YY1 sites are present in 52 % of the rp promoters. The ratio of aligned to unaligned sites in the human rp promoters is approximately 2:1 for GABP, 1:1 for Sp1 and 4:1 for YY1. The occurrence of unaligned sites in the mouse rp promoters is similar to that in the human rp promoters [see Additional file 3]. It is noteworthy that 76 % of the rp promoters contain at least one conserved upstream GABP and/or Sp1 site within 200 bp of the tsp. If unaligned sites are included, the proportion of human rp genes with an upstream GABP and/or Sp1 site is 92 %. Conserved consensus motifs for other ubiquitous transcription factors are present in a much lower proportion of the rp promoters, namely 12 % for the Box A-binding factor, 11 % for AP1, 10 % for ATF/CREB and 8 % for Nrf1.

### The rp gene initiator

**Figure 4:**
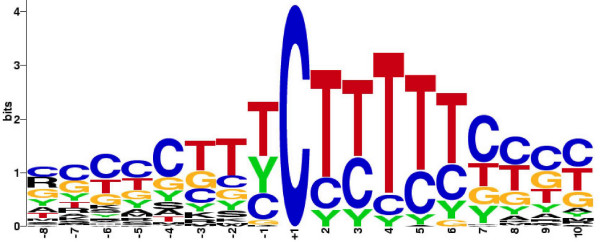
**The consensus initiator sequence of mammalian rp genes**. Seventy-nine pairs of orthologous human and mouse rp gene sequences were compared at positions -8 to +10 and the occurrence of each nucleotide or pair of nucleotides depicted by the height of the letters: A, G, C. T, Y = C/T, R = A/G, W = A/T, K = G/T, S = C/G, M = C/A. The tsp is the C at position +1.

The existence of a novel polypyrimidine initiator sequence in which the conventional A residue at the tsp is replaced by a C residue is well known. Moreover, the roles of this sequence in rp gene transcription and rp mRNA translation have been previously demonstrated experimentally for a few rp genes [7,40-42]. What is not known is whether there is a consensus initiator sequence that characterizes the entire rp gene set. To address this issue, I used the pairwise alignments of human and mouse orthologues to produce an occupancy matrix for positions -8 to +10 of the initiator [see Additional file 4]. With this matrix and the standard consensus rules, I determined that the rp consensus initiator sequence is (Y)_2_C^+1^TY(T)_2_(Y)_3_. A striking result is that T's are strongly preferred over C's at positions +2, +4 and +5. This preference, which is graphically illustrated in Figure 4, might be related to a transcriptional function of the initiator or a translational function of the TOP or to both functions.

### The extent of conservation of rp promoter features in non-mammalian vertebrates

**Figure 5:**
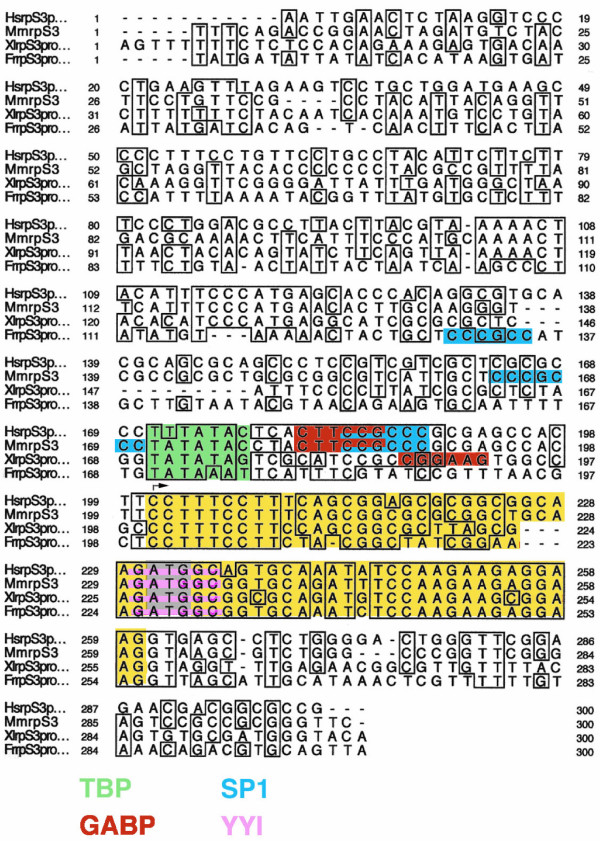
**Comparison of *rpS3 *promoter sequences in human (Hs), mouse (Mm), Xenopus laevis (Xl) and Fugu rubripes (Fr)**. Alignment by the ClustalW program with 100% and 75% identities enclosed in boxes. The first exon is highlighted in yellow with the ATG translation initiation codon in gray. Putative binding sites for TBP, GABP, Sp1 and YY1 are highlighted in green, red, blue and pink, respectively.

To determine the features of rp promoter architecture that are conserved over large evolutionary periods, I compared the promoter sequences of five chicken, six amphibian and five fish rp genes to their mammalian orthologues using the Clustal W multiple sequence alignment program. An example of such an alignment for *rpS3 *(Figure 5) shows strong conservation of both the coding and non-coding portions of the first exon, the TATA box motif, the initiator sequence, and a downstream YY1 site that spans the translation initiation codon. In contrast, the overlapping GABP and Sp1 sites, which are aligned in the human and mouse promoters, are not conserved in the amphibian and fish promoters. However, each of these promoters contains an unaligned consensus site for GABP or Sp1. A summary of the multi-sequence analysis for 11 rp genes (Table 2) indicates that the extent of conservation observed for *rpS3 *is fairly typical, although some small variations are evident. The conformance to the consensus initiator sequence is the same (90 %) for mammals and lower vertebrates. Yet, the particular C residue within the initiator that is used as the major tsp may differ slightly. Also the adjudged quality of the TATA box for TBP binding is not always the same. Nevertheless, except for the location of upstream transcription factor-binding sites, the general features of rp promoter architecture are usually well conserved over large evolutionary distances.

**Table 2:** Comparison of promoter features in mammals and lower vertebrates

**Gene**	**Species**	**Initiator**	**TATA**	**GABP**	**Sp1**	**YY1**	**Box A**	**AUG**	**Acc. No.**
*rpS3 *	Hs/Mm	TT**C**CTTTCCT	**+**	**1**	**1**	**1**		E1	AB061838, NM_012052
	Xl	GC-**C**------	**+**	**†**		**1**		E1	Z34530
	Fr	C---------	**+**		**†**	**1**		E1	X97794
*rpS6 *	Hs/Mm	SC**C**TCTTTTY	**–**	**3**			**1**	E1	X67309, D28348, Z54209
	Xl	**C**---**C**---C-	**–**					E1	AF020551
*rpS7 *	Hs/Mm	SY**C**TCTTYCT	**+/– +**				**1**	E2	Z25749, AB055774, NM_011300
	Xl	-**CC**-------	**–**				**1**	E2	X71081
	Fr	----------	**+**				**1**	E2	X94942
*rpS15 *	Hs/Mm	WY**C**TYTTCYG	**+/–**	**1**	**1**	**1**		E1e	M32405, AB055776, NM_001018
	Gg	**C**--C------	**+/–**	**1**	**†**	**1**		E1e	D10167
*rpS24 *	Hs/Mm	TY**C**TCTTTTC	**+**			**1**		E1e	AB062069, U12202, NM_011297
	Xl	----T**C**C--T	**+**			**1**		E1e	M33517
	Fr	-----C---T	**+/–**			**1**		E1e	AJ001398
*rpL5 *	Hs/Mm	GY**C**YTTTTCC	**+**			**1**		E1e	AB055762, NM_00969, mCG13589
	Gg	-G-**C**------	**–**			**1**		E1e	D10737
*rpL7a *	Hs/Mm	TT**C**TYTCTCC	**+**		**2**	**1**	**1**	E1e	X61923, X54067
	Gg	G**CCC**--T-TA	**+/–**			**1**	**1**	E1e	X62641
	Fr	-C------GC	**+/–**					E1e	Y15171
*rpL14 *	Hs/Mm	YY**C**TTCTCGC	**+/–**		**2**			E1e	AB061822, mCG22708
	Xl	--**C**--T--T-	**–**					E1e	X06552
*rpL18 *	Hs/Mm	ST**C**TTTCCGG	**–**		**1**	**2**		E1e	AB061825, mCG132477
	Xl	TC**C**-----TC	**+/–**		**†**	**1**		E1e	X05025
	Om	TC**C**---T-CC	**–**			**†**		E1e	AF240376
*rpL30 *	Hs/Mm	TT**C**CTTTCTC	**–**	**2**		**1**		E2	AB070559, K02928
	Gg	C--**C**-----G	**–**			**1**		E2	D14521
*rpL37a *	Hs/Mm	TC**C**TYTYYGG	**+/–**	**1**				E1e	NM_000998, NM_009084
	Gg	--**C**-----CT	**+/–**	**1**				E1e	D14167

Hs/Mm: Homo sapiens/Mus musculus; Gg: Gallus gallus; Xl: Xenopus laevis; Fr: Fugu rubripes; Om: Oreochromis mossambicus (tilapia). For the initiator sequences, **C** represents an alleged tsp based on various types of experimental evidence. For the transcription factor binding sites, only those sites that are conserved (aligned) between human and mouse are listed. A † symbol indicates that the promoter contains an unaligned site for the factor. Designation of AUG locations as in Table 1.

### Comparison of rp promoter features with those of non-rp TOP genes and other housekeeping genes

It is of interest to know which features of rp promoter architecture are specific and which might be common to other ubiquitously expressed genes. To this end, I examined the promoters of two additional sets of genes. One set consisted of non-rp genes that also produce translationally controlled mRNAs with 5'-terminal oligopyrimidine tracts (non-rp TOP genes). At the present, there are nine known genes in this category for which the tsp of at least one orthologue has been experimentally determined [see Additional file 5]. The non-rp TOP set contains genes encoding translation elongation factors (eEF1A1, eEF1B and eEF2), RNA-binding proteins (PABPc1 and hnRNPA1), a major nucleolar protein (nucleoplasmin/B25), a protein with tubulin-binding properties (TCTP/p23), and two genes that do not encode proteins, but rather have small nucleolar RNA-encoding sequences embedded within their introns (*gas5 *and *U17HG *). Annotated aligned sequences of the nine human and mouse non-rp TOP genes are presented on pages 1 through 9 of a supplementary file [see Additional file 6] and the results are summarized in Table 3.

**Table 3:** Non-rp top genes

**GENE**	**TATA Quality**		% H/M identity in 400 bp *	**GABP**		**SP1**		**YY1**	**Other**	**Initiator -2 to +7 †**	**AUG**
	**Hs**	**Mm**									
*eEF1A1 *	+	+	44			**1,**	2			TT**C**TTTTTC	E2
*eEF1B *	+	+	68	**2**		**1**				TC**C**TTTTTY	E1
*eEF2 *	+	+	55			**4**		**2**		KT**C**TCYYCC	E1e
*PABP cl *	+	+	66			**2,**	1			TT**C**CCCTTC	E1
*hnRNPA1 *	–	–	71			**2**				YT**C**CTTTCT	E1
*B23 *	+	+	73			**1,**	1		**BoxA**	TT**C**CYTGGC	E1
*Tpt1 *	+	+	67		1	**1**	1			GC**C**TTTTCC	E1
*gas5 *	+	+	55			**1,**	2			RK**C**YTTTCG	--
*U17HG *	+	+	43	**1**						ST**C**YYTYTW	--

* From -300 to +100 relative to the tsp.

† The bolded **C **is the tsp.

Other symbols are as in Table 1.

There are some notable differences in the promoter architecture of rp genes and non-rp TOP genes. First, GABP- and YY1-binding motifs, which are prevalent in the rp promoters, are rarely found in the non-rp TOP promoters (Table 4a). Second, eight of the nine non-rp TOP promoters have conserved TATA boxes that would be expected to bind TBP with high affinity, whereas only a third of the rp promoters have such TATA boxes (Table 4b). These differences were considered to be statistically significant when analyzed by Fisher's exact test (Table 4). The initiator sequences of the non-rp TOP genes resemble the rp consensus (89 % identical from -2 to +7) except for the roughly equal occurrence of C's and T's at +2.

**Table 4:** Comparison ofthe promoters ofrp genes, non-rp top genes, and other housekeeping genes

**(a) Occurrence of transcription factor binding sites: proportion of genes with at least one binding site.**					
**FACTOR**	**HUMAN RP GENES**		**HUMAN NON-RP TOP GENES**		**HOUSEKEEPING GENES**
	**Aligned**	**Total**	**Aligned**	**Total**	**Total**

GABP (5' of tsp)	54 %	68 %	22 % *	33 %	35%
SP1 (5' of tsp)	48 %	70 %	89 %	89 %	90 %
GABP or SP1	76 %	92 %	100 %	100 %	95 %
YY1 (3' of tsp)	52 %	59 %	11 % †	11 % †	10 % †


**(b) Potential for TBP binding to TATA boxes in the -25 region: proportion of genes in each category.**					
**TATA BOX QUALITY**	**RP GENES**		**NON-RP TOP GENES**		**HOUSEKEEPING GENES**

+	35 %		89 % ‡		30 %
+/–	25 %		–		20 %
–	39 %		11 % ‡		50 %

Based on analyses of 79 pairs of orthologous human and mouse rp genes, 9 pairs of orthologous non-rp TOP genes and 20 randomly selected human or mouse housekeeping genes. The symbols *, †, and ‡ indicate the percentages considered to be significantly different from the corresponding values for rp genes according to a Fisher's two sided exact test of the three sets of data. *: p = 0.086; †: p = 0.031 and 0.00075; ‡: p = 0.0029.

For the rp genes, the TATA box quality was scored as:

+ when both orthologues are + or when one is + and the other +/-,

+/– when both orthologues are +/– or when one is + and the other is –, and – when both orthologues are – or when one is - and the other is +/–.

For the non-rp TOP genes, both orthologues were either + or –.

The second set of ubiquitously expressed genes consisted of 20 housekeeping genes randomly selected from the eukaryotic promoter database [see Additional file 7]. The promoters of these genes also have an under-representation of YY1 sites compared to the rp promoters, and like both the rp and non-rp TOP genes, contain abundant motifs for Sp1 (Table 4a). The proportion of these genes that have TATA boxes with TBP-binding capability is similar to that observed for the rp genes (Table 4b).

## Discussion

The foregoing analysis of 79 mammalian ribosomal protein genes has revealed several features of rp promoter architecture, some of which are largely conserved over long periods of vertebrate evolution (about 450 million years) and others that are strongly conserved only in mammals. (about 90 million years). One highly conserved feature, present in over 3/4 of the rp genes, is the separation by an intron of the sequences involved in transcriptional and/or translational regulation from the sequences with protein-encoding function. In 47 % of the rp genes, the AUG translation initiation codon is in exon 2, and in 29 % of the genes, it is at the extreme 3' end of exon 1. It would seem that at an early stage of vertebrate evolution, these regulatory sequences were appended as discrete units to the loci containing the protein-encoding sequences.

The polypyrimidine tract that spans the tsp is present in all vertebrate rp genes. This tract can function as a transcriptional initiator [40,41] and also embraces the TOP sequence, which is essential for the translational control of rp mRNAs [7,42]. Based on the assignments of human and mouse tsp's used in the present study, the average lengths of the polypyrimidine tracts and TOP sequences are 12.2 bp and 8.2 bp, respectively. A compilation of conserved sequences in the -8 to +10 regions of orthologous human and mouse rp genes revealed the consensus sequence (Y)_2_C^+1^TY(T)_2_(Y)_3_. Thus, in addition to the C at the tsp, there is a clear preference for T over C at positions +2, +4, and +5. This preference may reflect a structural bias for transcription, e.g., ease of strand separation, or for translation, e.g., affinity of rp mRNAs for a putative repressor.

The presence of transposable element residues in the 5' flanks of the rp genes is noteworthy. Half of the rp genes contain an element (sines for humans, B1/B2 for mouse) within 500 bp of the tsp, and over 80 % of the genes contain an element within 900 bp. Some elements are moderately conserved between mouse and human, but most appear to be lineage-specific. These elements are unlikely to have any specific role in rp promoter function. They may be passively tolerated because the vast majority of conserved 5' sequence is confined to within 200 bp of the tsp. Within a segment from -200 to +100, the sequence identity between human and mouse rp orthologues ranges from 50 to 75 % with an average of 61 %, whereas the sequence match beyond -200 is of borderline significance.

The observation that 35 % of the rp promoters contain a TATA box motif at -25, which would be predicted to bind TBP with high affinity, and that an additional 25 % possess A/T-rich motifs, which might bind TBP with lower affinity, was unexpected. The assessed quality of these motifs for TBP binding, made according to rules established by a detailed structural study of TBP-DNA complexes [38], was largely conserved between human and mouse rp orthologues. Some of the promoters classified as poor (-) binders, e.g., *rpL32 *, might bind TBP weakly without help from an additional protein [43,44], so that the true proportion of rp promoters with TBP-binding capability might actually be greater than 60 %. Thus, contrary to earlier views based on an analysis of a small subset of rp genes, many of the rp promoters should not be classified in the "TATA-less" category.

When human and mouse rp promoter regions from -200 to +100 were scanned for conserved (aligned) transcription factor-binding sites with the rVISTA program [39], which is based on consensus sequences and matrix tables in the TRANSFAC database, three ubiquitously expressed factors that had previously been implicated in rp promoter activity predominated. Using high stringency criteria, I detected aligned GABP- and Sp1- binding sites upstream of the tsp and aligned YY1-binding sites downstream of the tsp in approximately half of the rp promoters. The occurrence of aligned motifs for other ubiquitously expressed factors is considerably less, i.e., only about 10 % for any single factor. Whereas Sp1 sites are also commonly found in the promoters of many housekeeping genes, including the non-rp TOP genes, the prevalence of GABP and YY1 sites appears to be a more prominent feature of the rp promoters.

GABP is a heteromeric protein consisting of an ETS family member, which has DNA-binding capability, and an ankyrin repeat-containing subunit, which greatly improves the stability of GABP-DNA interactions [45,46]. Two-thirds of the human rp promoters contain one or more potential GABP-binding sites upstream of the tsp, 79 % of which are perfectly aligned in the orthologous mouse rp promoters. Previous experimental studies have implicated GABP as a positive transcriptional regulator of the mouse *rpL30 *and *rpL32 *genes [10,14], the human *rpS14 *and *rpS6 *genes [20,22], and the *Xenopus rpL18 *gene [24]. The GABP sites that positively contribute to transcriptional activity are generally located upstream of the tsp, although in some cases, sites overlapping the initiator or downstream of the tsp may also be relevant. In the mouse *rpS16 *gene, GABP binding at the initiator decreases transcription activity both in vivo and in vitro [12]. For simplicity, only upstream, presumably positively acting, GABP sites were included in the tabulation.

YY1 is a zinc finger-containing protein with a variety of gene-specific functions, including transcriptional activation and repression, positioning of RNA polymerase II, and chromatin modification [47,48]. Fifty-nine percent of the human rp genes contain at least one YY1 site downstream of the tsp, 88 % of which are conserved in the mouse orthologues. Two functionally relevant binding sites for YY1 (originally termed "delta") were detected downstream of the tsp in mouse *rpL32 *[15]. In mouse *rpL30 *, a downstream YY1 interaction also contributed positively to transcriptional activity, but only when a strong upstream interaction with GABP was eliminated [11]. Interestingly, the vast majority of YY1 sites are located downstream of the tsp, and therefore, for simplicity, only downstream YY1 sites were included in the tabulation. The alignment of mammalian YY1 sites is frequently preserved in chickens, frogs and fish, in contrast to the GABP and Sp1 sites, which rarely have aligned counterparts in those species (Table 2). The aligned downstream site in *Xenopus rpL18 *was shown to interact with the frog homologue of YY1, but the functional significance of this interaction was not demonstrable with the reporter constructs that were used in these experiments [25]. The diverse activities of YY1 in different promoter contexts and its propensity for interactions with a wide variety of proteins [49], have led to the idea that it may have multiple mechanistic roles in transcriptional regulation. Which of these roles apply to the rp promoters remains to be established.

**Figure 6:**
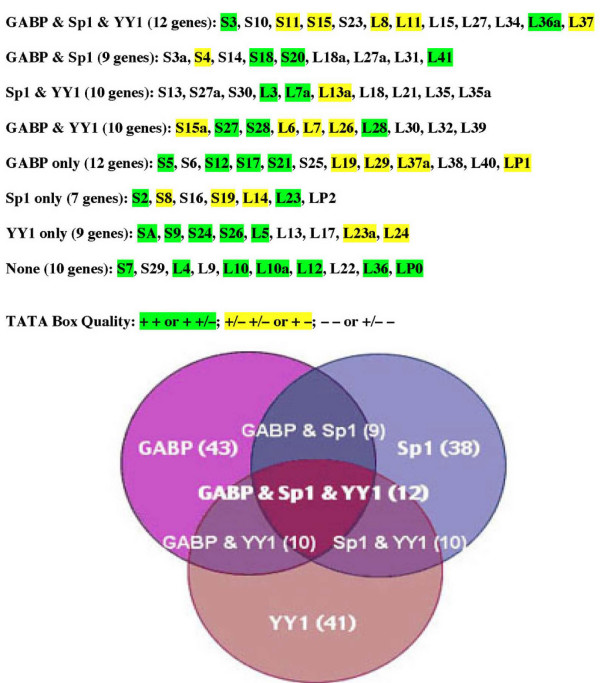
**Classification of rp gene promoters**. The data in Table 1 were used to classify the rp promoters into eight groups according to whether they contain aligned sites for GABP, Sp1, YY1, or various combinations of these factors. The promoters highlighted in green are those in which the TATA box quality of both orthologues was ranked as + or in which one ranked as + and the other as +/-. The promoters highlighted in yellow are those in which both orthologues ranked as +/- or in which one ranked as + and the other as -. Non highlighted promoters are those in which both orthologues ranked as – or in which one ranked as +/- and the other as -.

Based on the results of the analysis of rp promoter sequences, I have sorted the different rp promoters into eight classes according to whether they possess conserved binding sites for the three prevalent transcription factors, GABP, Sp1 and YY1 (Figure 6). Only 10 of the rp promoters do not appear to contain conserved binding sites for any of the three factors, and considering the high stringency used for the analysis, the true number may even be lower. Moreover, 8 of these 10 promoters contain non-conserved GABP, Sp1 or YY1 sites and/or conserved sites for other ubiquitously expressed factors. TATA boxes with predicted affinity for TBP are distributed among the promoters of all eight classes. This classification might prove useful for interpreting the results of experiments in which cell-specific or physiologically induced variations in the expression of different subsets of rp genes are observed. In addition, when more extensive data on the relative transcription rates of rp genes or the relative abundance of rp mRNAs become available, this classification might help account for any as yet undetected variability.

It is worth noting that some ribosomal proteins can also have extraribosomal functions [50,51]. Among the mammalian proteins that have been demonstrated or presumed to have additional functions are rpS3, rpS4, rpL5, rpL7, rpL10, rpL13a, rpLP0 and rpLP2. While, collectively, the promoters of the genes encoding these proteins do not fall into any particular class, most contain conserved binding sites for one or more of the three prevalent transcription factors and have TATA boxes with TBP-binding capability. Recently, the protein RACK1/Asc1p, which had previously been implicated in various signal transduction processes, was shown to have the properties of an authentic 40S ribosomal protein [52]. When I analyzed the promoter structures of the orthologous human (NM_006098) and mouse (NM_088143) genes that encode this protein, I observed several features in common with the rp promoters. The tsp of the mammalian *RACK1 *gene is embedded in a polypyrimidine tract that conforms perfectly to the rp initiator consensus sequence. Moreover, the promoter contains a TATA box of +/- quality, an aligned upstream binding motif for Sp1 and an aligned downstream motif for YY1, but no readily detectable motifs for other ubiquitous transcription factors. Interestingly, the features of this promoter resemble those of the *rpL13a *gene, which also encodes a protein with apparent pleiotropic function.

This analysis has highlighted features of rp promoter architecture that are shared by a high proportion of the rp genes. The evolutionary conservation of these features lends strong support to their functional relevance. Yet, superimposed on this general design are variations that confer certain idiosyncratic characteristics on each promoter. There does not seem to be a single master switch that co-regulates all rp genes at the transcriptional level. Rather, the rp promoters are tuned to respond to a combination of factors, including components of the general transcription machinery, a relatively small group of sequence-specific transcription factors, and modifiers of chromatin structure. The inherent functional redundancy and lack of dependence on any single factor are useful design features for genes that must be expressed in a broad spectrum of cell types and environmental situations.

## Conclusions

A sequence comparison of the promoter regions of all 79 orthologous human and mouse ribosomal protein genes has revealed several evolutionarily conserved features that are characteristic of a high proportion of the rp gene set. One such feature, which is also evident in the rp genes of lower vertebrates, is the separation by an intron of the sequences involved in transcriptional and translational regulation from the sequences with protein encoding function. Another conserved feature is the polypyrimidine initiator, which in mammals conforms to the consensus (Y)_2_C^+1^TY(T)_2_(Y)_3_. Contrary to previous impressions based on studies of a small subset of rp genes, the majority of rp promoters contain a TATA box or an A/T-rich motif at -25 that should theoretically have TBP-binding capability. Similarly, approximately half of the rp promoters contain conserved binding motifs for transcription factors previously implicated in rp gene expression, namely upstream GABP and Sp1 sites and downstream YY1 sites. Conserved motifs for other ubiquitous factors occurred much less frequently. Transposable element residues within 900 bp of 5'-flanking sequence were present in over 80 % of the rp genes; very little sequence conservation was evident more than 200 bp upstream of the tsp. Some of these architectural features were seen to be specific for rp promoters. From the results of this analysis, it was possible to sort the rp promoters into eight classes according to their possession of putative binding sites for GABP, Sp1 and YY1, and also to specify which promoters should have intrinsic affinity for TBP. This classification might prove useful for interpreting the results of experiments in which cell-specific or physiologically induced variations in the expression of different subsets of rp genes are observed.

## Methods

The rp gene sequences were extracted from three database sources [see Additional file 2]. The vast majority of sequences were obtained from the UCSC database [53], which conveniently uses uppercase and lowercase letters to distinguish exon and flanking/intron sequences, respectively. The remaining sequences were obtained from the ncbi [54], Celera [55] and the recently available RPG [56] databases. For alignment of human and mouse rp promoter sequences I used mVISTA [57], which is based on the AVID global alignment program [58]. The locations of repetitive insertion sequence elements were determined by the RepeatMasker program supplied with mVISTA and, in many cases, corroborated by ncbi annotations. Transcription factor-binding sites were detected with the rVISTA program and the FindPatterns tool of the GCG program. The analysis of non-rp TOP genes was made similarly to that of the rp genes. A graphic representation of the rp initiator consensus sequence was obtained with the Weblogo program [59]. For alignment and viewing of three or more orthologus rp genes, I used the ClustalW program [60] and SeqVu shareware l.0.1 (Garvan Institute, Sydney Australia).

## List of abbreviations

rRNA, ribosomal RNA; rp, ribosomal protein; tsp, transcriptional start point; TOP, terminal oligopyrimidine sequence; TBP, TATA-binding protein.

## Supplementary Material

Additional File 1 **ST1: Identification of the transcriptional start points (tsp's). **Comparison of experimental determinations of the tsp's of human and mouse rp genes. Click here for file

Additional File 2 **ST2: Promoter region sequences of rp genes. **Sources of rp sequences, amount of analyzed sequence 5' of tsp, and locations of insertion elements nearest the tsp. Click here for file

Additional File 3 **Aligned promoter sequences of rp genes. **Annotated sequence alignments of 79 orthologous human and mouse rp promoters and a key to the annotation. Click here for file

Additional File 4 **ST3: Characterization of the rp initiator. **Occupancy matrix for determination of the consensus sequence of the mammalian rp initiator. Click here for file

Additional File 5 **ST4: Promoter region sequences of non-rp TOP genes. **Sources of non-rp TOP gene sequences, the amount of analyzed sequence 5' of the tsp, and location of insertion elements nearest the tsp. Click here for file

Additional File 6 **Aligned promoter sequences of non-rp TOP genes**. Annotated sequence alignments of 9 orthologous human and mouse non-rp TOP promoters. Click here for file

Additional File 7 **ST5: Housekeeping gene promoters. **List of 20 housekeeping gene sequences extracted from the Eukaryotic Promoter Database, which were analyzed for GABP, Sp1 and YY1 binding sites and for TATA box quality. Click here for file
