# Supplementary material for: The architecture of mammalian ribosomal protein promoters
Source: BMC Evol Biol. 2005 Feb 13;5:15. doi: 10.1186/1471-2148-5-15 (PMC554972; doi:10.1186/1471-2148-5-15)
Supplement: Additional File 1 — ST1: Identification of the transcriptional start points (tsp's). Comparison of experimental determinations of the tsp's of human and mouse rp genes. [file 1471-2148-5-15-S1.pdf]

SUPPLEMENTARY TABLE 1

EXPERIMENTAL DETERMINATIONS OF THE TSP (CAP SITE)

OC-a: oligo-capping results reported in Yoshihama, et. al. (2002). OC-b: oligo-capping results in the DBTSS database. PE/S1: primer extension and/or nuclease protection experiments. Symbols show the relationships to the tsp's selected in this study: ★ or ✦, exact correspondence to designated tsp or the most frequent hit, respectively; ☆ or ✧, correspondence to an observed variant tsp; ●, exact correspondence to experimentally determined tsp; encircled numbers (①, □, etc) show the distance in nucleotides to the nearest reported tsp; x, substantially different from selected tsp; —, no data available. Hs, human; Mm, mouse; Rn, rat; Gg, chicken; Xl, amphibian; Om, fish.
*:the tsp was re-determined by Avni, et. al. (1994).

RP	Hs Ref Seq (NM_……)	OC-a Hs	OC-b Hs	PE/S1 Hs (Acc. No.)	Mm Ref Seq (NM_……)	OC-b Mm	PE/S1 Mm (Acc. No.)	PE/S1 Other (Acc. No.)
SA	002295	★	✦		011029	✧		
S2	002952	★	—		008503	①		
S3	001005	★	✦		012052	✧		Xl ① Z34530
S3a	001006	★	✧	● X87373	016959	①	● Z83368	
S4X	001007	★	✦	① L24369	009094	①	● L24371	
S5	001009	★	✦		009095	✧		
S6	001010	☆	✦	● M77232<br>① X67309	009096	①	● Z54209	Xl □ AF020551
S7	001011	★	✦	x Z25749	011300	①	x AF043285	Xl ● X71081
S8	001012	★	✦	● X67247	009098	①		
S9	001013	☆	□			—		
S10	001014	★	✦		025963	✧		
S11	001015	☆	✦		013725	—		
S12	001016	★	✧		011295	—		
S13	001017	★	✦		026533	✧		
S14	005617	★	□	● M13934	020600	①		
S15	001018	□	✦	x M32405	009091	①	● Rn D11388	Gg □ D90425
S15a	001019	★	①		170669	—		
S16	001020	★	✧		013647	—	● M11408	
S17	001021	★	✦	① M18000	009092	①		
S18	022551	★	✦		011296	①		
S19	001022	★	✦		023133	✧		
S20	001023	★	✦		026147	✧		
S21	001024	★	✦		025587	—		
S23	001025	★	✦		024175	①		
S24	033022	★	✦	□ U12202	011297	✧	x X71972	Xl ③ M33517
S25	001028	★	✧		024266	—		
S26	001029	☆	①		013765	①		
S27	001030	★	✦		027015	—		

S27a	002954	★	✧		024277	—		
S28	001031	★	✦		016844	—		
S29	001032	★	✧		009093	①		
S30	001997	★	—			—		
L3	000967	☆	✦		013762	①		
L4	000968	★	✦			—		
L5	000969	★	✦		016980	—		Gg ① D10737
L6	000970	★	✦		011290	✧		
L7	000971	★	④		011291	✧	● M29015	
L7a	000972	★	✦	● X52138<br>● X61923	013721	③	●④ X54067	Gg ● D14522<br>● X62641
L8	033301	★	✦		012053	✧		
L9	000661	★	✦		011292	③		
L10	006013	★	✦		052835	①		
L10a	007104	★	✦		011287	✧		
L11	000975	—	✦			—		
L12	000976	★	✦		009076	①		
L13	000977	—	✦		016738	✧		
L13a	012423	☆	✦		009438	✧	● X51528 *	
L14	003973	☆	✦			—		Xl ● X15678
L15	002948	★	✦		025586	—		
L17	000985	★	✦			—		
L18	000979	★	✦		009077	—		Xl ● X05025<br>Om ● AF240376
L18a	000980	★	✦			—		
L19	000981	★	✦	● X82201	009078	①	● Rn X82202	
L21	000982	★	✦		019647	□		
L22	000983	★	✧		009079	✧		
L23	000978	★	✦		022891	✧		
L23a	000984	★	✧			—		
L24	000986	★	✧		024218	①		
L26	000987	★	✦		009080	①		
L27	000988	★	⑦		011289	①		
L27a	000990	★	✧		011975	—		
L28	000991	★	✦		009081	①		
L29	000992	—	✧		009082	①	□ AF236069	
L30	000989	☆	✦		009083	①	● K02928	Gg ① D14521
L31	000993	★	✧		053257	✧		
L32	000994	☆	✦		172086	—	● K02060	
L34	000995	★	—			—		
L35	007209	☆	✦		025592	①		
L35a	000996	★	—		021338	③		
L36	033643	★	—		018730	✧		
L36a	021029	☆	✦			—		
L37	000997	★	✦		026069	①		

L37a	000998	—	✦		009084	✧		Gg ● D14167
L38	000999	—	✦		023372	①		
L39	001000	☆	✦		026055	①		
L40	003333	★	✦			—		
L41	021104	★	—		018860	①		
LP0	001002	☆	✦			—		
LP1	001003	☆	✦		018853	✧		
LP2	001004	☆	✦		026020	✧	● Rn X55153	

Avni, D., Shama, S., Loreni, F., and Meyuhas, O. 1994. Vertebrate mRNAs with a 5′-terminal pyrimidine tract are candidates for translational repression in quiescent cells: Characterization of the translational *cis*-regulatory element. *Mol. Cell. Biol.* **14:** 3822-3833.
