# Supplementary material for: The architecture of mammalian ribosomal protein promoters
Source: BMC Evol Biol. 2005 Feb 13;5:15. doi: 10.1186/1471-2148-5-15 (PMC554972; doi:10.1186/1471-2148-5-15)
Supplement: Additional File 2 — ST2: Promoter region sequences of rp genes. Sources of rp sequences, amount of analyzed sequence 5' of tsp, and locations of insertion elements nearest the tsp. [file 1471-2148-5-15-S2.pdf]

SUPPLEMENTARY TABLE 2

SEQUENCE ANALYSIS OF RIBOSOMAL PROTEIN PROMOTERS

h-s and m-s: database sources of human and mouse sequences, respectively (U, UCSC Genome; N, NCBI; C, Celera; R, RPDB). h-5'n or m-5'n: nucleotides of 5'-flanking sequence that were analyzed. h-5'is or m-5'is: nucleotides upstream of the tsp where nearest repetitive insertion sequences (sines or B1/B2 elements) are located.
Total rp genes analyzed: 32 small subunit and 47 large subunit = 79 rp genes

**RP**	**h-s**	**h-5'n**	**h-5'is**	**m-s**	**m-5'n**	**m-5'is**	**Other Orthologues**
SA	R	999	634	R	1000	372	
S2	U	802		U	1004	599	
S3	U	989	163	U	975	214	Xenopus and Fugu
S3a	U	1017	349	U	975	550	
S4X	U	1012	353	U	1049	880	
S5	U	975		U	979	721	
S6	U	1000	326	U	990	646	Xenopus
S7	U	1047	351	U	1045	389	Xenopus and Fugu
S8	U	1000	237	U	594	251	
S9	U	984	231	N	1038	379	
S10	U	1006	263	N	1036	255	
S11	U	1006	497	N	991	779	
S12	U	997	515	U	1044		
S13	U	1006	804	U	1001	747	
S14	U	1017	384	U	996	874	Chinese hamster
S15	U	1027	270	U	1003	176	Rat and Chicken
S15a	U	785	216	N	389	179	
S16	U	998		U	950	826	
S17	U	1001	464	U	1082		
S18	U	1002	729	U	978	726	
S19	U	1036	793	U	921	764	
S20	U	1004		U	555	411	
S21	U	1000	665	N	1050	600	
S23	U	1000	575	U	984	931	
S24	U	998	563	U	969	247	Xenopus and Fugu
S25	U	1000	328	N	1063	565	
S26	U	998	655	U	739	682	
S27	R	505	398	R	1000	440	
S27a	U	1002		U	881		
S28	U	1000	900	N	252		
S29	U	999	202	U	70		
S30	R	551		R	1000		
L3	U	999	701	U	979	654	
L4	U	1000		N	1030		
L5	U	987	229	C	1003	192	Chicken
L6	U	1053	124	U	1019	159	

L7	U	997	833	U	1032	199	
L7a	U	1001	293	U	1001	810	Chicken and Fugu
L8	U	954		U	985	341	
L9	U	1002	316	U	994	540	
L10	U	1000		U	879		
L10a	U	984		U	984		
L11	U	998	365	N	953	279	
L12	U	1000		U	988		
L13	U	1000	500	U	983	346	
L13a	U	999	435	U	995		
L14	U	1008	620	C	300	212	Xenopus
L15	R	1000		R	1000		
L17	R	1027	773	R	1001	160	
L18	U	1000	805	C	631	564	Xenopus and Tilapia
L18a	U	1000	818	C	659	385	
L19	U	1000	661	U	966	915	Rat
L21	N	962	486	U	913	270	
L22	U	999	432	U	1031	390	
L23	U	1000	519	U	990	324	
L23a	R	682	200	R	1019	197	
L24	U	997	258	U	842	410	
L26	U	998	840	U	1006	541	
L27	U	1009	430	U	1010	344	
L27a	U	999	730	N	1114	919	
L28	U	1000	280	U	1014	463	
L29	U	1043	262	U	976	197	
L30	U	996	144	U	1000	529	Chicken
L31	U	1000	638	U	1009	681	
L32	U	997	441	N	965	524	
L34	R	1014	335	R	967		
L35	U	1000	279	U	1000	422	
L35a	R	1009	548	R	998	473	
L36	U	1001	227	U	833	229	
L36a	R	729		R	1000		
L37	U	991	268	N	1050	539	
L37a	U	1000	300	U	983	428	Chicken
L38	U	956	130	U	1012	186	
L39	U	1000	743	N	1054	402	
L40	R	931	525	R	1000	477	
L41	U	1043	398	U	568	351	Carp
LP0	R	990		R	1000	345	
LP1	N	1040	468	U	999	732	
LP2	U	1002		U	994	580	
