# Supplementary material for: The architecture of mammalian ribosomal protein promoters
Source: BMC Evol Biol. 2005 Feb 13;5:15. doi: 10.1186/1471-2148-5-15 (PMC554972; doi:10.1186/1471-2148-5-15)
Supplement: Additional File 3 — Aligned promoter sequences of rp genes. Annotated sequence alignments of 79 orthologous human and mouse rp promoters and a key to the annotation. [file 1471-2148-5-15-S3.pdf]

ANNOTATION

Exons are highlighted in yellow; the transcriptiion start point is the first nucleotide in the exon sequence.

ATG translational initiation codons are highlighted in gray.

Sequences near -25 used for evaluation of TATA Box quality are highlighted in green.

Aligned transcription factor-binding sites are highlighted in pink.

Nonaligned transcription factor-binding sites are shown above or below the relevant sequence and enclosed in brackets.

Sequences of functional relevance as determined by transcription factor binding assays (gel shift ,DNAse I footprinting , methylation interference) and/or transient transfection experiments are underlined with asterisks (*****).

Sequences corresponding to transposable elements (sines for human and B1/B2 elements for mouse) are given in red letters.

```
seq1 = human rpSA
seq2 = mouse rpSA

57.6% identity.

      -200
seq1     agtcacaagcgccccagatccgaccaggatgccgctacccgtgacagcccagaagcccgc
          |      | ||  |   | |  ||  |  | |||       | |  |    |   ||||
seq2     tggggtctgtgcagcgactgcagccgcgcaggcgc------gggccactttaacttccgc
      -200                                                    <GABP>

                         <-Sp1>                  -100
seq1     tcct---gcgcgcaagcccgccttcctgaagaaaaagcccagtcccggcagcgttcttct
         |||    ||||   |||  |||  |     |||   || |     || ||| |   || |
seq2     tccgcgcgcgctacagcgggcccactcactgaatctgcgc--gtgcgccagaggcttttt
                                                                   <GABP

               <-Sp1>  ←--GABP-→             -50
seq1     ccggctccgcccttcttcc-----gctcgactttctttgccattggctgacaacggagta
         ||||  | |   |||| ||     |||   | | ||||||||||||||| |   ||  ||
seq2     ccggagcggaagttctccctttttgcttagcctgctttgccattggctggctgtgggtta
         -→    <GABP>

          -25 ATF/CREB                                        --YY1--
seq1     cataaggacgtcatttcctgctgcctgtCTTTTCCGTGCTACCTGCAGAGGGGTCCATAC
           |||||||||||||||| || ||| ||| ||| || |||||| |  || ||||||||||
seq2     tttaaggacgtcatttccggccgcccgtCCTTT-CGCGCTACCCGGGGACGGGTCCATAC
                      <GABP> <Sp1->                                +70


seq1     GGCGTTGTTCTGGgtgagttccgtgtagcgtccctggcgccttcc-agggctagaaaaat
         ||||||||||| |||||||  |  || | | ||  |||| || |  | | |  |  |
seq2     GGCGTTGTTCTTGgtgagtctcacgtggagccctcggcgtctccggacgccgcgggacgc


              +100
seq1     gagcttttc
         | ||||
seq2     gcgcttact
              +100
```

seq1 = human rpS2
seq2 = mouse rpS2

53.89% identity.

```
      -200
seq1     gct---------------------tttcgggtgacgtagattcccgggccgcggctgggg
         ||                      || ||| ||  |||        || |||||||  ||
seq2     gcgggcgcgagtatttatacgtccttccggctggtgta--------gggcgcggcttcgg
      -200                     <GABP>

                                                 -SP1--
seq1     ccggacgg-------gcaagagcactcagagcccggtgagggcggggcaccgcggagcca
         |  || ||       ||| | |||  |||| | ||| | ||||||| ||    || |||||
seq2     c--gagggaaagtccgcaggcgcagacagatcacggcgggggcgggacaagacgcagcca
                                                         -100
                                          <--SP1-->
seq1     atcacaggttgtcttataggagctcggcccgctcccgcttggggcggggctgcggagatc
         | ||||                    ||| ||     || || |    ||| |  ||||||
seq2     accaca--------------------gcctgcgggtgc-tgtgcgtaggcagtcgagatc

                              -25
seq1     tcgcggcgcttggcgtgctataaaagctgcttcccagtaggtctcg---ttCTTCTTTTC
         |||||   ||||| |  |||||||   | | | ||      | |||    |||||||| |
seq2     tcgcgcttcttggagcactataaattgtcccttccgcggcgcctccctcttCTTCTTCTT
                              -28       <GABP>

seq1     CGACAAAACACgtgagtcctgttggtcgctgcagcgggcctctgcg---tgggcggcggg
         ||  | ||||||||||||   |  || ||||   |  | || ||||   ||||| | |||
seq2     CGGGAGAACACgtgagtc---tatgttgctgggacctggctttgcggcctgggccgtggg


                                       +100
seq1     aatccgcggacatcgggtcgggtctaggctcccg
         |||||| ||||||| || | |    ||  |
seq2     aatccgtggacatccggacagacacagttttggc
                                       +100
```

```
seq1 = human rpS3
seq2 = mouse rpS3

59.88% identity.

      -200
seq1     aattgaactctaaggtcccctgaagtttagaagtcctgctggatgaagcccctttcctgt
           |   |           || ||| |                  || | |   |||||||
seq2     tttcaga-----------ccggaact-----------------agatgtctacttcctgt
      -200

seq1     tcctgcctacattcttctttccctgg-------------------------acgccttac
         ||| |||||||||     || |  ||                         ||||   ||
seq2     tcc-gcctacattacaggttgctaggttacaccccccctacgccgttttagacgcaaaac

          -100
seq1     ttacgta-------aaaactacatttcccatgagcacccacaggcgtgcacgcagcgcag
         ||   |        |||||| |||||||||||| |||   || | ||   ||| |||| |
seq2     ttcatttcccatgcaaaacttcatttcccatgaacacttgcaagggt---cgccgcgctg
              -100
                                                   -SP1--
                                  -25      -GABP-
seq1     ccctccgtcgtcgctcgcgccctttatactcacttccgcccgcgagccacttCCTTTCCT
         | |  |||| | |||| |||||| |||||  |||||||||||||||||||||||||||||
seq2     cgcggcgtcattgctcccgccctatataacctacttccgcccgcgagccacttCCTTTCCT
                        <-SP1->   -25

                       +25     -YY1--
seq1     TTCAGCGGAGCGCGGCGGCAAGATGGCAGTGCAAATATCCAAGAAGAGGAAGgtgagcct
         |||||||| ||||||| |||||||||| ||||| || ||||||||||||||||| ||| |
seq2     TTCAGCGGCGCGCGGCTGCAAGATGGCGGTGCAGATTTCCAAGAAGAGGAAGgtaagcgt
                       +25

                                            +100
seq1     ctggggactgggttcggagaacgacggcgccg-------
         |||||  |  |||||||     ||   |||||
seq2     ctggg--cccggttcgg-----gagtccgccgcgggttc
                                            +100
```

Note: TATA motif conserved in Xenopus and Fugu orthologs

seq1 = human rpS3a
seq2 = mouse rpS3a

75.49% identity.

```
       -200
seq1     -acacagcgcagcctccagtgagcccgcggcatgccgcgcggcctccccacacacgatgg
             |   |||    | ||  ||||||||||  || ||||| |||||| |||||||| ||
seq2     gctcccatgcacagcctagccagcccgcggcccgctgcgcgacctcccgacacacgaggg
      -200

                                                 -100
seq1     cgatgtcgcaaccacagtagaaagagtggcg-ctctctactgtcccggaattcttgaggc
         || |||||| | ||| |  | ||  | | || |   ||| |||| ||  |||||| | ||
seq2     cgctgtcgc-agcacggcgggaaacgagccgcc-agctagtgtcgcgagattcttaaagc
                                                 -100

         -GABP-              --AP1-              --SP1--          -25
seq1     cggaagaataacagggcaaaggtcacgtagacggcgcgccccgcccccgtacgcctaagt
         ||||||    | || |||| ||||||||||||||||| ||||||||   |     |||||
seq2     cggaagggcgaaagagcaagggtcacgtagacggcgctccccgcccggctcgcgttaagt

                         -GABP-
seq1     tctcgcgcgactcccacttccgCCCTTTTGGCTCTCTGACCAGCACCATGGCGGTTGGCA
         |||||||||| |   ||||||||||||||||||||| |  | ||||||||||||| ||||
seq2     tctcgcgcgagttgtacttccgCCCTTTTGGCTCTCCGGTCGGCACCATGGCGGTCGGCA


seq1     AGAACAAGCGCCTTACGAAAGGCGGCAAAAAGGGAGCCAAGAAGAAAGTgtaagtcgcga
         ||||||||||||| |||||||| ||||| |||||||| ||||||||||||| ||| |||
seq2     AGAACAAGCGCCTGACGAAAGGTGGCAAGAAGGGAGCTAAGAAGAAAGTgtgagtggcgg


       +100
seq1     ct
          |
seq2     at
       +100
```

```
seq1 = human rpS4
seq2 = mouse rpS4

69.5% identity.

      -200                                       <GABP>
seq1     aaggggagggaggaaatc--gcccttaggagacaccctctttccgcggcggttcctcaac
          |  |||  |||||||    |   || |||||     ||| || ||  | ||| | || |
seq2     gaaaggactgaggaaaaacagtttttgggaga----ttctctcagcctcagtttcccagc
      -200

                                                -100
seq1     tcgggacttctgacctcccaccagcagatggcaagaaagagtcccggcgcgccagcaaag
           ||||| |||||||||||||||||||||||||||| |||||||| || |||  | ||||
seq2     ctgggacctctgacctcccaccagcagatggcaagagagagtccccgcccgctgggaaag
                                                  -100

                  --SP1-                                          -25
seq1     ggctgagctgggcggagcagctgaaaatccg--gcgcgcgcagtctccagccccaatttc
               ||||||||||||| |  |   |||   |||||||||| ||||  ||| ||||||
seq2     tctgaggctgggcggagcaacccaggctccagcgcgcgcgcagcctccgaccctaatttc
                                                                  -25

                  -GABP-
seq1     tacgcgcaccggaagacggaggtcCTCTTTCCTTGCCTAACGCAGCCATGgtaagccgtt
         | ||  | |||||||| || |   |||||||| | |||| ||||||||||||||| |
seq2     ttcgttcgccggaagaaggcgagtCTCTTTCCGTTCCTAGCGCAGCCATGgtaaggcagg


seq1     tagttagcgagctcttaggacgtatccgcctccatcctcccccggccggggctgcccgag
              ||||||||||| ||  | ||| || |||||||||||    |    |||||||| |
seq2     ggtacagcgagctcttgggttgcatcggcttccatcctccctgaccgat-gctgcccggg


         +100
seq1     gttt-
          | |
seq2     ttatc
          +100
```

```
seq1 = human rpS5
seq2 = mouse rpS5

62.26% identity.
                       -200
seq1     -----------------tccgccaggc-acgcttctagggccgcttctgcaactgcgcat
                          ||||||||   |||    | | || | ||||  || |||||||
seq2     agctagtacttcagaggtccgccagaagacgggcgtggcgctgtttctaaaagtgcgcat
      -200


seq1     gcgcttgggctgcacccattccgtctagcccaaaaggttcctctacaaccttcctccgga
                 |||  ||||          || |||| ||  ||||| || |||  | |  ||
seq2     --------gctttaccc---------ggctcaaagggcgcctct-catcctagcgcttga


                                                       -50    -GABP-
seq1     ttggtctatagggacacgatggccttttacgcaagcgtcacccgtgctgacccggaagtt
         |||||   |  | || |  |   | | | |||  ||||| |     ||||||||||||||
seq2     ttggttggtgagcacgctgttttcatctgcgcgtgcgtctctacctctgacccggaagtt
                                                       -50

                    -25
seq1     ttcttcccagttaaaagtgttggcccgcggcgcgcggcCTCTTCCTGTCTGTACCAGGGC
         |||        |||||| | | | |||| |||||||||||||||||||||||| ||||||
seq2     ttcgctttgcataaaagcgctagtccgctgcgcgcggcCTCTTCCTGTCTGTATCAGGGC
                    -25


seq1     GGCGCGTGGTCTACGCCGAGTGACAGAGACGCTCAGGCTGTGTTCTCAGgtgagaccgcc
         ||||||||||| |||||||| ||| |||| || ||| ||| |  |||||||||| | ||
seq2     GGCGCGTGGTCCACGCCGAGCGACTGAGAAGCCCAGTCTGCGCCCTCAGgtgaggctgcg


                       +100
seq1     gcggggccggggatccta
         | ||| |  ||  | |
seq2     gagggacacggagttcag
                       +100
```

```
seq1 = human rpS6 (D28348)
seq2 = mouse rpS6 (Z54209)

56.47% identity.

      -200
seq1     aattacacgcgggtttgccttacc--------agactaccaccactggcac---------
         |       ||||      || |||        | || ||   | | | |||
seq2     a-------gcgg------ctaaccccggtgataaacgacagaccccgtcaccaggaaggt
      -200


seq1     -----aacctcaga------cccacacccaaccgactttacctcccaggccctgattaat
              || || | |      | | |  | ||||| |    |   |||    ||  |||||
seq2     ccaggaagctaaaatgacagctcgctgcaaaccggc----ctctccatagtctagttaat


      -108      -GABP-  <-SP1>           -GABP-                     --
                **************           ******                     **
seq1     ct-cgcccggaagtaccgcccacccatgctcacttccgctatcccgtacttctgctcatc
         || |||||||||||| |||||  | |   ||||||||||            | |||  ||
seq2     ctacgcccggaagtaacgcccctctaaagtcacttccgc------------cggctgttc
       -97

         BoxA--                 -25                -GABP-
         ******                                    ******
seq1     tcgcgagaactgaaagcgcctatgtgacctgcgctaagcggaagttggccCTCTTTTCCG
         ||||||||||| | |||| ||||||| |||| | ||||||||||| || |||||||| ||
seq2     tcgcgagaacttagagcg-ctatgtgccctgggttaagcggaagtcgggcCTCTTTTTCG
                                -25

                                                      <YY1->
seq1     TGGCGCCTCGGAGGCGTTCAGCTGCTTCAAGATGAAGgtaggtgatggtggcgagtgtta
         || ||||||  ||||| || ||||  |||||||||||||||| |     ||| ||
seq2     TGACGCCTCCCAGGCGCTCGGCTGTGTCAAGATGAAGgtaggcgtccccggccag-----


                                              +100
seq1     gactgggtttggg-----------gaacgtgaatcgagtc
         | | | ||  ||            || |||     | | |
seq2     ggccgagtgaggccgcccgagcccgagcgt-----gcggc
                                              +100
```

```
seq1 = human rpS7
seq2 = mouse rpS7

56.07% identity.

      -200     <GABP>                       <SP1/GABP>
seq1     aacgctcggaaacggcaccaaagtacgaatcctagggcggaaaagcgttaccaagacact
          |||||                    ||   |||  | | | |||||  |||   || ||
seq2     -acgct--------------------gacgactaccgggaacaagcgaaaccggcacgct
       -200

seq1     cgtcccca-------------------gagccgcttcctgggactctctagcctc-ctac
         | ||| |                    || |   || | || || |||||| |   | |
seq2     catcctcccgcattagttcccagtagggaactattttccggtacactctaggcggtccaa
                                            <GABP>

                           -80 <GABP/SP1>              <GABP>
seq1     cgcttctcagt-gatgttccggtttccgccctcctcctcgcgctgtttccgcctcttgcc
         | ||| ||||  ||  ||||||||||| ||| ||| ||  ||||||  || ||||||  |
seq2     ctcttttcagaagagcttccggtttccacccacctacttccgctgtccccacctcttttc
                        <GABP>  -75           <GABP>

                       -25       --BoxA--
seq1     ttcggacgccggattttgacgtgctctcgcgagatttgggtCTCTTCCTAAGCCGGCGCT
         ||| | | || |  | |||   | ||||||||||   |   ||||| ||||||||||| |
seq2     ttccgccacccggatatgagccgttctcgcgagagccgtccCTCTTTCTAAGCCGGCGAT
                       -25


seq1     CGGCAAGgtaggttggcggcctgctctccgacagaacttttcttcttgggttgaggaaaa
         | |  ||||||||| |||| | ||     | | |   ||||   |   | ||| || ||
seq2     CCGTCAGgtaggttcgcgggccgcaatgtggccgcggtttttcgcagagtttgggggaac


                          +100
seq1     cgccttttggagtcaggccct
          | || |||| |   | ||
seq2     agactcttggggggcgtccgg
                          +100
```

Note: BoxA is conserved in Xenopus and Fugu

```
seq1 = human rpS8
seq2 = mouse rpS8

59.5% identity.

      -200
seq1     cccc---------------------cagcccagggtc---ggaggcgagtcgccgggggt
         |  |                     | ||||| ||||   |||   ||| |    |||||
seq2     cagcttaaggagcccgccctccagaccgcccaaggtcaagggatttgaggc----ggggt
      -200           <-SP1->       <-SP1>                 <---SP1--->


seq1     cggcagaggtcagcagcctgcgagaccctcctaggctcgtg-gccctaggcgcggcagct
         | ||    ||  |||  | ||  ||||||||   |  |  | | || |||| ||
seq2     ccgcgtgagttcgcacgccgcaggaccctcccctgtgccagcgtcccaggctcg------


seq1     gacacgtaagtctcgtcgcccgacgttgtttc-ggcgctcagaaacaacgtaaagtaaag
                      ||  ||||   ||  ||  | |||   | |||   |||||||| |
seq2     ------------ccgcggccccaggtccttcccggcaaccggaagagacgtaaagaa---
                                                <GABP>

          --SP1--          -25
seq1     gggcggggcagcgttttacaaaccgaaccgtgaatctttgcggtttCTCTTTCCAGCCAG
          |||||||| | |||||| |||||| |||||||| ||||||| || ||||||||||||||
seq2     -ggcggggctgagttttagaaaccggaccgtgaagctttgcgcttcCTCTTTCCAGCCAG
                           -25


seq1     CGCCGAGCGATGGgtgagtgtcgctctgcattgaggcgggtgaagggaggttgagctcaa
         ||||||||||||||||||||| |||  |    | | ||||    ||| |   |||||| |
seq2     CGCCGAGCGATGGgtgagtgttgctgcgggccgggtcgggccgcgggggagcgagctcca


                               +100
seq1     tcaggccccatcctgcttcacagcct
         || | | ||||||||| || |  |
seq2     tccgtctccatcctgcctcccgacgc
                               +100
```

seq1 = human rpS9
seq2 = mouse rpS9

50.0% identity.

```
      -200                                                         <
seq1     ccctacttctcaggtttcc------------------------------actccctggcc
               ||||  | ||  |                              ||  |||  |
seq2     -----attcttggattaaccaaatgtcaccatagccctttaatcgcctaaccacctcact
           -200

         GABP>
seq1     ggaaaaaacctagtcctcccaggttagcacgccgctctagcccagcctcacgtctccact
         ||       | ||||  |||||   ||  |||||||||||||   |  | |||||| |
seq2     gg-------caagtcggcccagcgaag--cgccgctctagccggcctgctcgtctctatg


                                     -80                          ATF/
seq1     gcttctcagccagccaacgcctcttctgattggctctgacgtgcgtggtgcgtgaaaacg
         |||| | |             ||||||||||||   | || | | |||||||||  |||
seq2     gctttcctg-------------cttctgattggcgtagtcgcgagcggtgcgtgatgacg
                                -80

       CREB/AP1                   -25
seq1     tcacgagacgccggcgttactataagagcgcagccgtggcgcttgcgcgcctCTTTCTCA
         |||| | | | || ||||||||||||| | | | |    |||     |   ||| |||
seq2     tcactacaaggcgacgttactataagacctcggtccaaccgc-----cttgtCTCTCTTT
                             -25


seq1     GTGACCGGGTGGTTTGCTTAGgtgaggtgcggcggtgtgctttttctctaggg------t
         ||  | ||   |  | | |||||||         |||| ||  || |  ||        |
seq2     GTCTCGGGCCTGAGTTCGTAGgtga---------gtgttctgattgttgagttcgccttt


                 -YY1--                         +100
seq1     ttgggttggatggtggc------ccgggccttccgagtttcc
         | ||| | |||||||        | | ||| | ||| | |
seq2     taggggttgatggtgtaaggtaacggagccattcgaatct-t
                                                +100
```

seq1 = human rpS10
seq2 = mouse rpS10

63.52% identity.

```
      -200
seq1     gtactgatagagcacgcaagctcagaagttcccggcgg---cggtttcctagtgcgtgcg
                 | | | | |  |||   ||  ||| || ||   |||  |    | | | |||
seq2     -------cacaactcacgggctagcaatctccaggtgggggcggggtggaggagggggcg
             -200                             ←--SP1--→        ←-SP1-


seq1     tgag---------------tccctcaggcggtcaggctccgtggtcagatccgccagttc
          ||                ||  | || |||  |  || ||||           ||   ||
seq2     ggaagcgtagcgggatggctcgcacatgcgtcctagcaccgt----------ccccactc
         ->

                                                   <SP1>-50 --Sp1-
seq1     ttcaggcaccgccgaggccaccccaggctccccgcgggtcctggcgggggcgggtccac
          |||||||| ||||||| ||||||||||  | |||||||||||  ||| |||||  || |
seq2     ctcaggcactgccgagggcaccccaggcagcgcgcgggtcctgcagggaggcggagccgc
                                                       -50

                -GABP--23       --Nrf1-
seq1     gccagcccggaagagacgcagcaccgcgcatgctccttCCTTTCCAGCCCCGGTACCGGA
         | ||||||||||||||||| || ||||||| || ||||||||||||||| | |   ||||
seq2     g-cagcccggaagagacgctgcgccgcgcacgccccttCCTTTCCAGCCTCCGCCTCGGA
                    -25

                                              +50           --YY1--
seq1     CCCTGCAGCCGCAGAGgtgagtttctgtggcgttcgagttccatcggctcccatccgggc
         |||||||||||| | |||||||| ||  ||||| || | |||||| |   ||||||||||
seq2     CCCTGCAGCCGCCGCGgtgagttcctccggcgtccgggctccatctgtcgccatccgggc
                                              +50

                       +100
seq1     tatcctgccgccttagcg
               || ||| | | |
seq2     cgcagcgctgccgttgag
                       +100
```

```
seq1 = human rpS11
seq2 = mouse rpS11

53.13% identity.

      -200                     --BoxA----                --SP1--
seq1     cctaag--------------actctcgcgagacaccgtctagcacttcccgcccgtcagt
         |||                 | ||||||||||    |    |  |||||||||| | |||
seq2     cctccacatcgtgctcctgaaatctcgcgaga---tgataggatcttcccgccccttagt
      -200


seq1     ccttcacagtgcccgaatcgcccgctggtttaagtgcagcctgtcaatcagagca-----
         ||| | | ||    |   ||  ||                  | || ||  || |
seq2     cctcccccgtcatggcggcgtacg------------------gacagtcccaggaacgcg


                        ←-SP1→                           -ATF--GABP-
seq1     -gctctcg----aggccccgcccaaggccgtgctgcgtttctccttacgtcacttcctct
          |||||||    | |  || ||||   |||   || |  |  ||   |||||||||| |
seq2     ggctctcgccggaagtacctcccacctccg---tgaggataaccccgcgtcacttccgc-
                  <GABP>                                      -50

                              -25
seq1     ccagcccctgcgtaatcgataaggaaacccggacgctgctgcccctttCTTTTTTTCAGG
         || | ||  ||||  |  |||||||| || ||| || ||    ||| ||  ||| || ||
seq2     cccgacctcgcgtggtgaataaggaagccggga-gcggccctgcctctCCCTTTCTCCGG
                             -25

                   --YY1--
seq1     CGGCCGGGAAGATGGCGGACATTCAGgtgcggactcggggttggatgcca----------
         |||||||||||||||||||||||||||| ||  |     ||||  | ||
seq2     CGGCCGGGAAGATGGCGGACATTCAGgttcgagcgtttagttgctttcccccgacgcttc


                                         +100
seq1     -gggtgcggggtccgccttggccttcaggggcgcg
          | |||  | ||   ||||||| |         |
seq2     ggtgtggagcgtatcccttggcgt---------cc
                                        +100
```

```
seq1 = human rpS12
seq2 = mouse rpS12

62.33% identity.

      -200
seq1     gtagtgcctgttgcactcgccaaggaccggcctggcagagat-------agaaaactcgc
              |||     | || | ||| | | | ||  || | |||       ||||| |  ||
seq2     tccacgcc-----ccctgggcaaaggctgccc--gctgcgatgcctgctagaaatccagc
      -200

                                       -GABP-
seq1     agtacgtttgcctcagaaccgcctcaatccggaaacagaaactccccaggcccggcactg
          |     || || | ||  | ||| ||  |||||   ||||||||  |||| ||||||
seq2     cgcgttcttaccacggacgctcctaaaggcggaagtggaaactcctaaggcacggcacca


                                          --Nrf1--
seq1     cgggagccacctctttcgagggtgggagtgcacatgcgcacaggggacctgaaaaaccct
         | |||||||||||||| | ||| ||   |||||||||||||||  | |  |   | | ||
seq2     ccggagccacctctttagtgggaggagttgcacatgcgcacagaagtcgagcggagcgct


         -25
seq1     taaataccggcatgcgtgcggatgaggCCTCTTTCCCTGCCGCCGCCGAGTCGCGCGGAG
         ||||||||| ||||||  |      ||||| || ||||||||||||||| | ||||||||
seq2     taaataccgtcatgcggccaccgccggCCTTTTCCCCTGCCGCCGCCGACTTGCGCGGAG
         -25


seq1     GCGGAGGCTTGGGgtaagt-tgagcgaggcggcaggggggtgtattggttataatttgtg
         ||||| ||| ||||| ||| ||| |   ||    | |  |   || |  | |    || |
seq2     GCGGAAGCTCGGGgtgagtgtgatcccagctcgtgcgccggcgatagcgtgtctactgcg


             +100
seq1     gctcgttg
          || |
seq2     ccttg-gc
             +100
```

```
seq1 = human rpS13
seq2 = mouse rpS13

60.75% identity.

      -200
seq1     g----accaaagac------------cgatgcatgccgggatgaggctggattgacgatc
         |    || | ||||            ||  |||  || |    |  ||
seq2     gcaagactagagacaagagttcaaggcgtagcaacccagccatacact------------
      -200


seq1     tcttgcgacgctgaaatgcatttcgggagatgtagtcacacagacgggcggtggctgcgc
         |   ||  ||| |  |||| ||  ||||   |||||| | | | |   |||||||  | |
seq2     taccgcctcgccgccatgccttctgggacgcgtagtctcccgggc-tccggtggccacac


             -90  --SP1--                              -AP1--
seq1     agcgtagctccgcctcctgcgtgagaggaagcggggacgtgtgaggtcacttcctgcgtg
            ||||| ||||| || ||| ||||| || ||  |  | |||  ||||||||||  |||
seq2     tatgtagccccgcc-cccgcgcgagagcaaacgtcggtgagtggcgtcacttcct--gtg
           -90

                  -25                                             -YY1
seq1     ttgggtggcttcctgcggcgtttccactctcgctctCCTTTCGTTGCCTGATCGCCGCCA
           |  ||  |||||| | ||||||||   |||| ||||  || | |||| || | ||||||
seq2     cggcttgatttcctgtgccgtttcctacctcgttctCTCTTTGCTGCCAGACCTCCGCCA
                  -25

         --
seq1     TCATGGGTCGCATGCATGCTCCCGGgtgagctcggggcatcaagccggattgctgggcgg
         |||||||||||||||| |||||||||||||| || |||  | | |  | |||| |||| |
seq2     TCATGGGTCGCATGCACGCTCCCGGgtgagcccgcggcgccgaccttggttgcggggctg


                    +100
seq1     ggggtgggaggaagac
         |||   || |
seq2     gggcgcggggctgaga
                    +100
```

```
seq1 = human S14
seq2 = mouse S14

62.22% identity.

      -200
seq1      cc------------------gacccgctcgcagcgacaaccagccctctacctcttttcg
           |                  ||  || || |||| |||   ||| | |  | ||||||
seq2      tccgagagttctcataggcagattcggtcacagcaacacgtagcac-cggcttctttt--
      -200


seq1      ctctcccttaagtaataaaccgtctttccttatgacgagtcttaaactctttgggaggaa
                        |||  ||  ||| | |   | || || |||   ||  ||| |
seq2      --------------ataggccaacttccgtaccggcg-gttttagcttcgctggaaaccg
                                 <GABP>

                      -GABP-                    -AP1--     -50   -GABP-
seq1      taatgccggcgtcttccggaacccgacc--tcgccccgtgacctcagaggtatacttccg
          | |||  ||  |||||||||| ||  ||  || | | |||||||||||| || |||||||
seq2      tgatggaggtttcttccggaagcctgcccctctctcagtgacctcagagatacacttccg
                                                         -50

               -GABP-                                  -GABP-
               ******  -25         --SP1--             ******
seq1      ggacacggaagtgacccccgtcgctccgccccctcccactCTCTCTTTCCGGTGT-GGAG
          | | ||||||||||  ||||||   ||||||| |||  ||||||||||||||||  ||||
seq2      gaagacggaagtga--cccgtccgcccgccccttccgcctCTCTCTTTCCGGTGGAGGAG
                     -25


seq1      TCTGGAGACGACGTGCAGgtagg-aggcccgggcgc-gacaatcggggggcatcctgcgg
          |||||||||||||  |||||||| ||||  ||||   || ||||| | ||||||||  ||
seq2      TCTGGAGACGACGATCAGgtagggaggcttgggccttgagaatcgtgcggcatcctttgg


                             +100
seq1      cgaggggaccctgtggggcttgg
          | ||||    ||| |||
seq2      ccagggccgtctgcggg---atc
                             +100
```

```
seq1 = human rpS15
seq2 = mouse rpS15

51.54% identity.

      -200
seq1     t---------------------cccact--------ccgtggaggaagcggctctgcaga
                              | ||||        ||    ||| |||  | |
seq2     aaaccagggacagagagacagacacactaacaaaaaccccaaagggagccacgc-----c
      -200


seq1     cctcaggcgg----cggctgctctaatccctgcagcctgtgttttgccctccagacggca
         |||| || ||    ||||                ||  | || | || ||||| ||| ||
seq2     cctccggagggtagcggc----------------gcttttgctctggcctccggacagcg


                                                  -GABP-
seq1     gagggc------gcgtgttcgccgccgggaaagcggaagcgcgtgtgcgcgcacggccga
         | ||||      | || | |||       | |||||||||   || |||         |
seq2     gggggcaatggcgagtatccgc-------agagcggaagctgttgagcg---------gt


           --SP1--                              -25--Nrf1--
seq1     gggggcgggacatgccggcagt-ctcgcgataactgcgcaggcgcggaccaaagcgatCT
         || |||||| | | |||||||  ||||||||||||||||| |||| | ||  | |   ||
seq2     ggaggcgggccctcccggcagagctcgcgataactgcgcaagcgcagcccgta-cttcCT
                                               -25

                                   --YY1-
seq1     CTTCTGAGGATCCGGCAAGATGgtgagtgttgcgatttggcgcgtctctgc---cgggcc
          ||| ||| | ||| ||||||||||||| ||       |||| |||| |||   ||||
seq2     TTTCCGAGTAACCGCCAAGATGgtgagtattcgtgcacggcg-gtctgtgctcccggg--


                                                     +100
seq1     tatccggctccatccaacctctgaccgtctcgcggggcc----gca--------
         |||||| | |||||              ||| ||||| ||    |||
seq2     tatccgtcgccatc--------------ctcccggggccctttcgcaagactctg
                                                             +100
```

NOTE: GABP & YY1 sites conserved in chicken

seq1 = human rpS15a
seq2 = mouse rpS15a

59.2% identity.

```
      -200
seq1     acgaacggc------tcaccttgcctctcccaggccccaccgggggaggtcgattccatc
           | || ||      |  |   ||  ||  |||  ||||||           |||   ||
seq2     --ggactgccgcgagtttcagagcgactagcag--cccacc-----------att---tc
        -200


seq1     gactctcaactgccaacccctgaggaggctccgcctttccctcaccctcgtccttctcct
          | | ||| |||||   ||| |||||||| |||     ||||| |  | ||  | ||
seq2     tagtttcatctgcccctcccagaggaggccccg-----ccctcgcattggttgtcctata
                                      ←---SP1----→

                                                              --BoxA--
seq1     aggc--------------ggtccttgtttttgccgggcctttga-----aagtctcgcga
         ||                |||  || |||||   | |  | |||     |||||||||||
seq2     agaaatcacagggttttgggttttttttttttaagtg--tgtgaccatgaagtctcgcga


                    -GABP- -25                               --YY1--
seq1     tagctctcccgcttcccataactccgtgcgctctcccctcccgtcCTCTTTCCGCCATCT
         |||| || ||||||||||||| |||||||| |  |||  |||   ||||||| |||||||
seq2     tagcgcttccgcttcccataattccgtgcgtttgcccggcccactCTCTTTCTGCCATCT
              <GABP>       -25                               +10

                                                           <-YY1>   --
seq1     TTCCGCGCCGgtgagtagcactctctgagagctccaatttcatccgtctgccatcggcgc
         | || |||||||||||     | | ||| | ||||   ||||||||| ||  ||| | ||
seq2     TCCCTCGCCGgtgagtgagtgtttgtga-aactccgtgttcatccgtgtggtatctgtgc


         YY1-                 +100
seq1     catcctgcaatctaaggttcggtct-
         |||||    | |    ||||| | |
seq2     catccctttaccccgtgttcgctttc
                               +100
```

```
seq1 = human rpS16
seq2 = mouse rpS16

73.85% identity.

      -200                         --SP1--                  ←SP1-→
seq1     cccgccccttccg---cccacagagc-ccgccacttccgcctc-acagggcccgcccgct
          || | || ||||   ||| || ||| ||||| ||||| |  | |||  |||| | | ||
seq2     tccccaccctccgggtcccgcacagctccgccccttcccctccgacaccgccccctccct
      -200                     -175*********             ←-SP1-→

                           --SP1--             ←-SP1-→
seq1     ctgagcgcctaagggccttcccgccgctgaccagacgccccgcccgttagccgcaacaga
               |||| | ||| ||||||||||    || ||| ||| | |    ||  | ||
seq2     -----tgcctgaaggcgttcccgccgcaattcacacgtccc-ctctcaggcttcgacgtc
                           -125

                            ?NucFactor              --Nrf1-- ?NucFactor
seq1     agccgtaaagctctctcccgtcgcgacgcagtgctcaaggcgcctgcgcagaccctgaaa
         ||||||||||| | || | |||| |||| ||  ||||| |||||||||||||||||||||
seq2     agccgtaaagcccgcttctgtcgtgacgtagcactcaatgcgcctgcgcagaccctgaaa
                             *********      -50              *********

       -25
seq1     ag-cggccagggtggcccctagctttCCTTTTCCGGTTGCGGCGCCGCGCGGTGAGGTTG
         |  ||||  || |||||||  |||| ||||||||||| ||||||| |||||||| ||
seq2     aatcggctgggttggccccgcgcttcCCTTTTCCGGTCGCGGCGCTGCGCGGTGTGGAGC
         *******                          **********


seq1     TCTAGTCCACGCTCGGAGCCATGCCGTCCAAGGGCCCGCTGCAGTCTGTGCAGGTCTTCG
         ||  |     ||||||||| |||||||||||||| ||||||||||| |||||||||||||
seq2     TCGTGCTTGTGCTCGGAGCTATGCCGTCCAAGGGTCCGCTGCAGTCCGTGCAGGTCTTCG


           +100
seq1     GACGCA
         ||||||
seq2     GACGCA
           +100
```

seq1 = human rpS17
seq2 = mouse rpS17

60.06% identity.

```
      -200                             <GABP>                    <-SP1
seq1     gg----------gagcgg--------ggagcggaaagggag------gagccgtcggggg
          |          |  |||        |||| |||||   ||      |||| | ||| |
seq2     cgcccaagagaagcacggaactatttggagtggaaaccaagacggccgagctgccggcgt
      -200

         -->                                             ←-SP1-→
seq1     cgggacagaca--------ccctgcccaccggaggggcgcgccaccgccgcccccgctcc
           | ||    ||        ||| |||||| |             | | |  || | ||||
seq2     gcgcacgcgcagactttcgccccgcccacag------------ccaggcagcctcactcc
                            ←-SP1-→

                                                             -60   -GABP
seq1     actccgccagggggcgctgtgtggctcccgagtaggctcagagcgaccgtggccgggcgg
         ||||||| ||||| ||||||| | ||| || |                   ||| |||||
seq2     actccgctaggggccgctgtgggactctcgcg------------------agccaggcgg

         ---                      -25
seq1     aagcggctttctggcctaagctttaacaggcttcgcctgtgcttcctgtttcCTCTTTTA
         ||||||||||||||||   ||||| | |||| || | |||| ||||||||||||||||||
seq2     aagcggctttctggcc-gcgcttt-ataggcctctcttgtgtttcctgtttcCTCTTTTA
       -50                        -25

                                                   ←YY1→
seq1     CCAAGGACCCGCCAACATGgtaggtgttttggctcgaggccgccatcctccacaatcgtc
         || | |||||||||||||||||||||||| |   | ||| | |  ||||   ||||| ||
seq2     CCGA-GACCCGCCAACATGGTAGGTGTTTGGCTCCCAGGGCTCTGTCCTTTTCAATCCTC

                                       +100
seq1     ccccatctgcagcttcccgcggtcctcag-ccc
          ||||||  | |||   ||||| |   || ||
seq2     gcccatcctccgctcgtcgcggcctcaagtccg
                                       +100
```

seq1 = human rpS18
seq2 = mouse rpS18

64.08% identity.

```
      -200
seq1     agccccggaaccttcgctgttctcttacctatgaaccttacgaactgtaaagaaaggcgc
          || ||                    ||  | ||||  |||| ||   | |||| | ||
seq2     cgcgcc-------------------cacacaagaacggtacg-acgacagagaaggccga
      -200

      -140 -GABP-
seq1     accggaagttgtggtacc--------------------caagcca--tactctcataaat
         |||||||||| | |  ||                    |||  ||  | ||| | ||
seq2     accggaagttatagctccggtcccttccaaaactgtctcaatacaactcctcccttaggg
      -160

                                    -GABP-         -60       --SP1--
seq1     ccagccaggtcgcgctgaaacagtttccggaagcacttctcctag-atcgcaccgcctct
           || | ||  |    ||| || ||||||||||     ||||  | || || ||||||||
seq2     ggagacggggag---agaagcaatttccggaagtggacctccggggatagccccgcctct
                                                                    <-

                         -25         -ATF--GABP-
seq1     tcctcctggaagctatataatgatatcgcgtcacttccgctctCTCTTCCACAGGAGGCC
         ||| || |||| ||||||||   |    ||||||||||||||||||||||||||||||||
seq2     tccgccaggaatctatataagacttatacgtcacttccgctctCTCTTCCACAGGAGGCC
        GABP>            -25

seq1     TACACGCCGCCGCTTGTGCTGCAGCCATGgtaagactggaatccgtgccgtgatccagcg
         ||||||||||||  ||||| || ||||||||||||  |||||||| |||| ||||| |||
seq2     TACACGCCGCCGTCTGTGCCGCCGCCATGgtaagagcggaatccgcgccgggatcctgcg

                            +100
seq1     gcatcgcagctcgggcaaggaaa
          ||||| |||  ||||| ||| |
seq2     acatcggagccggggcagggaga
                            +100
```

```
seq1 = human rpS19
seq2 = mouse rpS19

71.7% identity.

      -200
seq1     aaacccagg--cctccacgcgcgaccccttggccctcccctttacctctccacccctcac
         | | ||     |||||         |||  | |||| | |||  | |||||||||||  |
seq2     agatcccttatcctcc--------tcccgagtcccttctcttggcttctccaccccttcc
      -200

                     ←-SP1-→                       -100 --SP1--
seq1     t---agacaccctcccctctaggcggg-gacgaact----ttcgccctgagagaggcgga
         |   | | |||| ||| | | ||  || || |||      |||||||| ||| |||||||
seq2     tcttacagacccgcccattttgggtggagatgaagccatattcgccctcagaaaggcgga
                  ←-SP1→

                    ---BoxA--     -55   ---BoxA--
seq1     gcctcagcgtctaccctcgctctcgcgagctttcggaactctcgcgagaccctacgcccg
         |||     || || |  |||||||||||||  || ||| ||||||||||| | |||||||
seq2     gccctctggtttagc--cgctctcgcgagcactccgaaatctcgcgagactcgacgcccg
                                          -50

            -25
seq1     acttgtgcgcccgggaaaccccgtcgttccCTTTCCCCTGGCTGGCAGCGCGGAGGCCGC
         ||||||||||||||||||||||||||||||||||||||||||||||||||||||||||||
seq2     acttgtgcgcccgggaaaccccgtcgttccCTTTCCCCTGGCTGGCAGCGCGGAGGCCGC
            -25


seq1     ACGgtaagcgggggctccgagctg-gaccgggcgcgaggtggcagggccggacgccgaag
         ||||| || | |||||| | |  | | | | ||||| ||   | ||||||| |      |
seq2     ACGgtgagtgagggctctgggtcgcggctgtgcgcggggccacggggccgggctttctgg


                +100
seq1     cctcagagcgc
         || || || ||
seq2     cccca-agagc
                +100
```

```
seq1 = human rpS20
seq2 = mouse rpS20

63.25% identity.

      -200
seq1     cggggaccccaccctgcgggacttcggctcctccgtgcggacagggggcgcccgcag--c
          |     ||  |    | ||||   | |||| ||| | || ||| |||||| |||    |
seq2     tg----tcctccgagtcaggacagggactccgccgagaggccagagggcgcgcgcgcgtc
      -200

         -140     --SP1--
seq1     cgtgcctttcccgccaactccactagtgctc----cagctcgcccttgtgcgaccgcctc
         |  ||||||||||||||||   |  |  | |    | ||   |||    |||||| | ||
seq2     ctcgcctttcccgccaactaggcggggacgcagcccggcctcccccacggcgacctcttc
         -140                ←SP1-→

                             -GABP-       -50
seq1     tagggcccgggatatttcctgttccggggcgtgtgggacccggatgcaagcgtgctatat
           |||  ||||    ||||||||||||| |     | ||||||||||   |||||| |||
seq2     gcggg--cggggcggttcctgttccgggtcacaaagcacccggatgcgcacgtgctttat
           ←---SP1----→                            -50

        -25
seq1     aagcgttgctcaagtcccacccctttCTTTTTGAGGAAGACGCGGTCGTAAGGGCTGAGG
         |||||     |     ||  ||| | |||| ||    || | |||  ||  | |||  ||
seq2     aagcgcccggcgccgtccggcccctcCTTTCTG----AGCCCCGGCGGTGCGCGCTACGG
        -25

seq1     ATTTTTGGTCCGCACGCTCCTGCTCCTGACTCACCGCTGTTCGCTCTCGCCGAGGAACAA
         |   | || | ||  |||||   |||||||||||||||||||||||  || |||||||||
seq2     A-GCTCGGCCAGCCTGCTCC--GTCCTGACTCACCGCTGTTCGCTCCTGCTGAGGAACAA

                                                              +140
seq1     GTCGGTCAGGAAGCCCGCGCGCAACAGCCATGgtgaggcgg----------cagtcggcg
         ||||||||||||||   |||||  |||||||||||||| ||          ||  ||||
seq2     GTCGGTCAGGAAGC---CGCGCCTCAGCCATGgtgaggtggggggcgtcgccacgcggcc
                                                              +140
```

```
seq1 = human rpS21
seq2 = mouse rpS21

52.26% identity.

      -200
seq1     cgacaacgacccccacgacgccccgcgccccagggccagcaggctggggtccg-------
           |   |    |    | ||      |   ||||| | |  | ||| | |  |
seq2     gcatgtcttggctggaggcg------gagacagggaccgatgcctgtgctttgtgcggaa
      -200                                                         <GABP

                              -GABP--GABP-        -110
seq1     agaacc------tccttgc--cggaagcggaagtgcgtcacggcgacggccggcaagcac
         ||| ||      || ||||  |||||||||||||           |   |   ||| ||
seq2     agagccaggctttctttgcaccggaagcggaagt----------taaaacacacaaacag
         >                                          -110

                                                    -GABP-    -50
seq1     gcaccgcgcagactcgctgtggtgg----------cgtgacccggaagcgctcgccgagg
         ||  |         || | ||||||          | |||||||||||| ||  ||||||
seq2     gcctc---------cggtatggtggatacgagtaccctgacccggaagcacttaccgagg
                                                              -50

                   <SP1->  -25
seq1     cccctccttgcccgccgatatctctgccgggtgactagctgcttcCTTTCT---CTCTCG
          ||| || | |   || |||   |||| | |  || |||| ||||||||||   ||||||
seq2     acccgccctcctgcccaatagtgctgcggaggcacgagctacttcCTTTCTGCGCTCTCG
          <-SP1->          -25


seq1     CGCGCGGTGTGGTGGCAGCAGgtgtggcgcgcggcgcgggcttcgggctcagggctgggg
         |  |    |||     |||||||  | | ||  ||| |   |  | |  | ||||  |||
seq2     CTGGACAAGTG-----AGCAGgtaggcctcggtgcgggctgtgtgcgagcggggc-cggg
           220            230       240       250       260

                                     +100
seq1     ctggggcgggagtgggggctt---ggacgcg
         ||||| ||||   || |   |   ||| |
seq2     ctgggccgggctgggcgcagttggggaggtc
                                     +100
```

```
seq1 = human rpS23
seq2 = mouse rpS23

65.06% identity.

    -200
seq1     a-------accatgcaaattagatatctctgaatttcttgcaaattaaataagacgcaga
         |       ||   | |||| | ||  || || ||||  | || |||||| || || |
seq2     aggaaataacagcgtaaatgaaattgctttggatttgatacatattaaacaaaactc---
    -200


seq1     ttctggctcaggaaagtgatgcaaacgcgtcgttt--tcaaaggagagaccccagcctcg
          | || ||||  ||||||  |  |  |  ||||||  || |   | | ||     || ||
seq2     -tgtgtctcacaaaagtggaggcactgaatcgtttcctccacccacacac----tccccg


                                            -50  --SP1---
seq1     ggtcaggcgcggcgcagacagcggcgcggggtccttggctgggcggggcttgctcgcggt
         |  ||||||| ||   |||||| || ||||| | | ||||||||||||||| | |||  |
seq2     gtacaggcgccgcttggacagccgcacggggccttcggctgggcggggctt-cccgcaat
                                            -50

          -25       -GABP-                                          -
seq1     ggcttgtggctccttcctgcggtgcttctCTCTTTCGCTCAGGCCCGTGGCGCCGACAGG
         | ||||||| ||||||||||    | |||||||||| ||  |||||||||||||| || |
seq2     gccttgtgggtccttcctgcacctcctctCTCTTTCTCTTGGGCCCGTGGCGCCGGCAAG

         -YY1--
seq1     ATGGgtgagctgtt-gtggccggtttaaggg--cgctgcaagcgggacttggggtcttgg
         |||||||||||||  | ||| ||  | ||||  ||| |||   |||   | | |    ||
seq2     ATGGgtgagctgtgcggggctgggctgaggggccgccgca---gggctctagagctcggg


                +100
seq1     ggacgggcgggc
           |||| |   |
seq2     atacggcctatc
                +100
```

```
seq1 = human rpS24
seq2 = mouse rpS24

58.67% identity.

      -200
seq1     tag-----------tcggctcgagttgttctggttctggattggtcagtctagagttc--
         |             ||   | |||| | ||   ||| ||      |   | ||||  |
seq2     tgtgtaaccatacatcaatttgagtggatccaattcagg------ccagccagagcccag
      -200

                                     <-SP1->
seq1     --caggggtgtggccaaaagtctgtgaggcggggccgtaggcagcctaaatcaggcatcg
           | |||| ||||| ||  |   | |||||| |||   | | |   |   |||||||| |
seq2     gtctggggcgtggctaagggctagcgaggcgtggcttcaagtactttcggtcaggcattg


                                                                 <GABP
seq1     gcgcggtcagcctcgtggcgcgc-ccacgcccccacgccggctcttcccggggtccttcc
           |  |||  || |  |||     |||||||||| ||          | |||||||||||
seq2     ctgtagtctaccccaaggccttagccacgcccccgcg----------ctggggtccttcc


         >        -25
seq1     gtgcgcgttgatatgattggccggcgaatcgtggttCTCTTTTCCTCCTTGGCTGTCTGA
          ||||||||||||||||||| ||||     | ||| |||||||||||||   | | ||
seq2     ttgcgcgttgatatgattggtcggcagtgagcggtcCTCTTTTCCTCCTCTCCAG-CTCC
                  -25

             +30 --YY1--
seq1     AGATAGATCGCCATCATGgtgagtctccctgggcccgtgcagtcatctgccgcgtatccg
          |     | |||||||||||||||||||  |||| |        ||| | ||| ||||||
seq2     GGCGCCGTAGCCATCATGgtgagtctcctcgggctctcagcactatccgtcgcttatccg
            +30

          --YY1-      +100
seq1     agccatccgtgg-tccc
          ||||||| ||  |
seq2     cgccatccctgcctgtt
                      +100
```

Note: A/T block conserved in Xenopus
YY1 site conserved in Xenopus and Fugu

```
seq1 = human rpS25
seq2 = mouse rpS25

55.65% identity.

      -200
seq1     cgctatagtatgcgtcacaactagc---------gcagactccggcagtatttaggcgg-
          || | || ||  ||  |   | |          ||| | | | ||| | ||  | |
seq2     agc-agagaattagtggctgttgggtgacccgcagcaaagtgcagcaattttacgaccag
      -200

                                                   <GABP>
seq1     ----------tgcggcttgggaactagaatccacttcctgtcttccgcctcaggctagag
                   ||| | |||       |||  |||  ||  | | ||  | ||  | ||||
seq2     tctcagagtttgcaggttg-------gaagtcacgccccctatcccatccca--caagag


                                               -60          -GABP-
seq1     ggcgagcgcttcgccgtgggacttcttctgcctggctccgccttttgccccggaagtact
         |  ||  | || |||  |            | ||||||| ||  ||||||||||||||||
seq2     gcgga--gtttagccaag-----------acgtggctccacccattgccccggaagtact
                                                 - 60

                      -25
seq1     cacagcggacggtggttttttgggcccgtttctgagcagcgCTTCCTTTTTGTCCGACATC
         | |    | | | ||||| ||||   |||      | | ||||||||||| |||||||||
seq2     ctctcgaggcagaggtttctgggattgttgggccaccgggCTTCCTTTTTTTCCGACATC
                      -25


seq1     TTGACGAGGCTGCGGTGTCTGCTGCTATTCTCCGAGCTTCGCAATGgtaagcttc---ag
         ||  ||||||||| |||       | | |||| ||||||||| ||||||||   |   ||
seq2     TTAGCGAGGCTGCTGTGGTCTACACGACTCTCTGAGCTTCGCCATGgtaaggcccgcgag


                                 +100
seq1     gggtgtgaagtcgccggcgttc----t
         || || | |||    || ||||
seq2     ggttgagcagt----ggggttcagagg
                                 +100
```

seq1 = human rpS26
seq2 = mouse rpS26

53.01% identity.

```
      -200
seq1     aaatagtcccatgtgcactttgttccatggataaataaacactaggaacgcatttccacc
                ||  ||     |||  ||     ||| |  |   ||||| ||  ||  | |||
seq2     -------ccactgatatttttcgtc-----atagaccatttctagggacttatcacaacc
             -200

seq1     ctagatttcagcagaaatgctgaatgtaaaggaatatttgag------------------
         ||| ||||||   ||||  || | |    ||| | ||| |||
seq2     ctacatttcacaggaaactctaagtaattaggtaaattcgagcataatagctgctaggga


seq1     -taaagtgagttgccgttcttgaagcccgtctcctaaggattctcc----cggtgtccgc
          ||||  || || | | ||              || ||| || |      | | ||  ||
seq2     gtaaatagaattacaggtc-------------gctgagggttttttttttcagcgtgagc


                           -25
seq1     gtagggatctcatgctatataggagggccctgccaggcaccgtctCCTCTCTCCGGTCCG
         ||||| ||||| | ||||||| | ||| |  || || | |||  |||||| ||  || |
seq2     gtaggaatctcgtactatataagcgggtcgggcaagccgccggatCCTCTTTCGAGT-CT
                           -25

                  --YY1-
seq1     TGCCTCCAAGATGgtgagtcttcttgcgtggtgagggtgggggttcgggtgcagactctg
         || |||||||||||||||| |    |||||||||| | | |||   ||||       |||
seq2     TGGCTCCAAGATGgtgagtgtcgccgcgtggtgagcgcgcgggcctgggt------actg


                                     +100
seq1     ggattgtgg---------ggaagtgagagcct
         || | |  |         ||| |  || | |
seq2     gggtagaagtagatctatggacggtagggacc
                                     +100
```

seq1 = human rpS27
seq2 = mouse rpS27

70.81% identity.

```
       -200
seq1     -caaattagcaaggtaaccctctcaacatggtgacgcatatgtgtcacaactaacagtta
          |||| ||| ||| |||| |||||| |||||  ||||    | | | | |  | |  |||
seq2     tcaaaatag-aagctaactctctcaccatggctacgcgcccgcgacgcgaggagccttta
      -200

              <GABP>
seq1     aagaccttccgaaaatggcagctcccttgtctccccactgcggcggatcaaagta--aga
           |||||||   ||||||||| ||    | |   ||  |||    || || ||||  |||
seq2     cggaccttctacaaatggcag-tcaacgggcattccgttgcattcgaacagagtagaaga


                                            -GABP-              -25
seq1     cgttccaaacatggtggcacaagatccttgcgtcatttcctgtagtgtgctctatataag
         | |||| || |||| |   ||| | |    ||||| |||||||    |     |||||||
seq2     ccttccgaagatggcgaagcaaca-cggctcgtcacttcctgtcccatatcagatataag
          <GABP>                                -50

                 -GABP-                                        --YY1--
seq1     gggcaggatttccgctttcgctcCTTTCCGGCGGTGACGACCTACGCACACGAGAACATG
         |  ||||||||||||||||||||||||||||||||||||||||   |  |||||||||||
seq2     gaacaggatttccgctttcgctcCTTTCCGGCGGTGACGACCT--CCCTACGAGAACATG
                                                              +70


seq1     CCTgtgagtgctttggtccaggtttcggcggagatctcgctgttctgtccgaactctccc
         ||||||||| | ||||||||||||||| | |||| |||||| ||  ||||| ||  |||
seq2     CCTgtgagttccttggtccaggtttcgacagagaactcgctcttgggtccgcacggtcct


          +100
seq1     ct--c
         ||  |
seq2     cttgc
          +100
```

```
seq1 = human rpS27a
seq2 = mouse rpS27a

56.88% identity.

      -200
seq1     aaa-------------------cctacagtttcgaaagcattccgaaggctaaagtgaga
           |                   |  |   ||||||   ||||| ||  | ||||  ||||
seq2     tgaggcattttttttagttgagcacatcatttcgaggccattctga--ggtaaaccgaga
      -200


seq1     aataagcccaggctagggagaggagaaacgaagttcacgtcctagtctggcaccgggttg
         ||            || | |   ||||||  ||   |||  | | |||||||| | |||
seq2     aa------------agagcgtaaagaaaccgagcgaacgagcaaatctggcactgcgtta


                                                   -60      --SP1---
seq1     gattgtcgctgggacg-----gcagtcaggcatttggtgtggtcgcctaaggggtgggtc
         ||  | |||     ||     ||   ||||||  | |||||| ||||||||||| |||||
seq2     gacagccgcgattccgctgcagcgcgcaggcacgt-gtgtggccgcctaaggggcgggtc


             <-SP1>       -25                                        -
seq1     cttcggcgggagctccgggaaaccccgtgggcctgcgcggcgttCTTCCTTTTCGATCCG
         ||||||        || ||| ||||||| ||||  |||   | ||||||||| |||||||
seq2     cttcgg--------ccaggagaccccgtcggcc-acgctcggatCTTCCTTTCCGATCCG


         -YY1-
seq1     CCATCTGCGgtgggtgtctgcacttcggctgctctcgggttag---caccctatggtgcc
         |||||   |||| |||| ||| ||  ||| || ||| || |||   | |     |  ||
seq2     CCATCGTGGgtgagtgtatgctctgtggccgcgctctggctagtggcgctacgcgtcgct


                                 +100
seq1     ttctcttgtgatccctgacctaacctg
          || |  |||    | || ||||
seq2     ctcacgggtgtcgtcggatctaa---t
                                 +100
```

```
seq1 = human rpS28
seq2 = mouse rpS28

67.08% identity.

      -200                          -GABP-
seq1     c-------cgcgcaccccggacgcccagttccgcagccgctggatgagacgggtggcgga
                 | |||||||| ||||||||||||||||||   ||||| |  || || |||||
seq2     gaacagcacacgcaccccagacgcccagttccgcagcttttggataacgcgagtagcgga
      -200

               <GABP>
seq1     cgccatcttccgtccgcgatactgaagggcgcagccggtgacgtctccaccgctggggct
         |||||| |||| |||||    | ||||| |   || ||||||| |||   | |  ||   |
seq2     cgccatattccttccgc---tccgaaggac---gctggtgacgactctgtcccgaggatt
                   <GABP>

                                                   <-SP1>
seq1     gctgggaaaaacgcggcgtg--gtatcgcgaga-cgggagtgggcggtgct-agaacgtc
          ||||| |     | |||||  || |||||||| ||  |||| |  ||| | ||| |
seq2     cctgggta-----ccgcgtgcggtctcgcgagagcgaaagtgaggcgtggtcagagccga


             -25                  -AP1--                             -YY1
seq1     ctataaaggctctccccgaagcacgtgactcCTCTCCGCCAGACCGCCGCCGCGCCGCCA
         ||||||| |   |||||    ||||||||||||||||||||||         ||||||||
seq2     ctataaaaggcatcccctgctcacgtgactcCTCTCCGCCAGA--------TCGCCGCCA
             -25

         --
seq1     TCATGGACACCAGCCGTGTGCAGCCTATCAAGCTGGCCAGGgtgaggtgggggcccgaat
         |||||||||| || || |||||||| ||||||||||| ||||||||| |   ||  |  |
seq2     TCATGGACACGAGTCGCGTGCAGCCCATCAAGCTGGCTAGGgtgaggcgactgcgggtgt


                        +100
seq1     ttgggggc--------agg
         || |   |        ||
seq2     ttagaaacctgaataaaga
                        +100
```

```
seq1 = human rpS29
seq2 = mouse rpS29

42.81% identity.

      -200
seq1     tcggataccggcgcagaatagcactagaagctgtggtatggtgacgtcatcaactgggcc
         |
seq2     t-----------------------------------------------------------


seq1     agcccacaacgcctctaagatttcattttactcacccagcgaaacaacctgaccacactg

seq2     ------------------------------------------------------------

                                                                  -27
seq1     cgcacgcgtttcctttgagcactgcattctgggtaaactgt------ctcaaaaatttga
                          |||  || ||| ||||||   ||       |  |||| |||||
seq2     -----------------agcgttgtattgtgggtagcgtggtagctccaaaaaactttga
                                                                  -27

seq1     agagcgcatgcgtgggccagcttcttCCTTTTACCTCGTTGCACTGCTGAGAGCAAGATG
         ||||||| |||| | |||  ||  |||||||  ||||||||  |  ||||  ||||||||
seq2     agagcgcctgcgcgagccgactcgttCCTTTCTCCTCGTTGGGCGTCTGAAGGCAAGATG


seq1     GGTCACCAGCAGCTGTACTGGAGCCACCCGCGAAAATTCGGCCAGGGTTCTCGCTCTTGg
         |||||||||||||| |||||||| ||||| || || |||||||||||||| |||||||||
seq2     GGTCACCAGCAGCTCTACTGGAGTCACCCACGGAAGTTCGGCCAGGGTTCCCGCTCTTGg


           +100
seq1     tgagaa
         |||| |
seq2     tgagga
           +100
```

seq1 = human rpS30
seq2 = mouse rpS30

67.84% identity.

```
      -200                                --Sp1---
seq1     cgccagccttctgtgcgcgcaactgt-ctggtcccgccccgtcctgcgcgagctgcctgc
          |   | | | || ||||||| | |  || | |||||| |   ||||||| || | |
seq2     tgagtgtcatatgcgcgcgcagccgggctcgccccgcctccctctgcgcgcgcaggc-ca
      -200

                                          --Sp1-- -100          --Sp1-
seq1     ccaggcaggttcgccggtgcg---agcgtaaaggggcggagctaggactgccttgggcgg
         | |||   |  ||||  ||||   ||||| ||||||||| ||| |||| ||  |||||||
seq2     caaggaccgaacgcc--tgcgtacagcgt-aaggggcgg-gctgggacggcggtgggcgg


                                            ATF/CREB
seq1     tacaaat-agcagggaaccgcgcggtcgctcagcagtgacgtgacacgcagcccacggtc
         ||||||  | ||| || ||||||||||||| ||||||||||| |||||||||   ||||
seq2     tacaaaccaccagcgatccgcgcggtcgcgctgcagtgacgtcacacgcagcaggcggtg

       -25     --Sp1--                      --YY1--
seq1     tgtactgacgcgccctcgcttcttcCTCTTTCTCGACTCCATCTTCGCGGTAGCTGG---
          | ||   ||||||| | ||||||||||||||| |||||||||||||||||||| |
seq2     aggacgtgcgcgcccccacttcttcCTCTTTCTTGACTCCATCTTCGCGGTAGCAGCAGC
       -25                                          +20


seq1     ---GACCGCCGTTCAGgtaagaatggggccttggctggatccgaagggcttgtagcaggt
            | |||| |   |||| |||   |||      |||||  | | || |||       |||
seq2     GGCGTCCGCGGGCTAGgtgagacacgggggactgctgggccaggagcgct------cggt


                +100
seq1     tggctgcgggg
          | | ||||
seq2     agccagcggtc
                +100
```

```
seq1 = human rpL3
seq2 = mouse rpL3

56.15% identity.

      -200
seq1     acccctcgccgtagagtgtg--taattt----------ctgtctccgaagctgacgtcaa
              ||| ||  | || |   |||  |          |  |||||| ||        |
seq2     ----gtcgacgaggcgtttcgctaagcttgaacccaatcattctccgtag--------ac
          -200

                      ←GABP-→
seq1     cttctgaacgaaagctaggagc--ggaagagcgtgcgtggaaatggcgctcggtacgtgc
         | | |     ||  ||||  |   |||| |  ||| ||  ||||| |  ||| | || ||
seq2     cctatttgtcaatcctagtgggcgggaaaaatgtgtgt-aaaatgacattcgatgcgcgc
                            <-SP1->

                                           -60    <GABP>-SP1--
seq1     ccc-cgacctgtcgtctgccgcgggggcgcgctcgcacgccggaaggggcggggccag-a
         ||  ||||| | || ||   |||    | || ||||||| |  | ||||||| ||||  |
seq2     ccttcgacccgacgcct-atgcgtcaacacgttcgcacg-ctcaggggcggagccatca
                                                    -50

                   -25                                         <-YY1>
seq1     tttggctttatatagcggacccgtaaggccgaccggcCTCTACCGGCGGGATTTGATGGC
          |||||||||||||||   | |      | |  ||  ||||| | ||||  | ||  |||
seq2     cttggctttatatagcctgcactggctcctggtcgctCTCTATCTGCGGCGTGTGGCGGC
                   -25

           --YY1-
seq1     GTGATGgtatgtgttgtaatggcggccgtttcattgtcaccggcgtcggcacggct-gaa
         | ||||||| || | |  || || ||| | | | |    |    || |||  |||| | |
seq2     GAGATGgtacgtatggccat-gctgccttctaacttggtctcctgtgggcctggcttgta


                      +100
seq1     tccgcgacgcatcgccg
         | | |  | ||||| |
seq2     ttcacctcccatcggc-
                     +100
```

```
seq1 = human rpL4
seq2 = mouse rpL4

67.65% identity.

         -200
seq1     ---tccctattttctaagaattattattacgtttggaagaaaatagataccgtaatttcc
            |||||  ||||||| | | || |||  ||| || |||||  ||||   | |||
seq2     gagtccctcctttctaatcactcttttaactttt-aaaaaaaatgaatacataattttaa
      -200


seq1     cacaaataaagaaataacactctcgtttttacttttgtagtgtggtagagagatattttt
         ||||  | | | ||||| | |  | |||  | ||||    ||| | ||| |||||  |
seq2     cacacgtcacgcaataatattacctttttgaaatttttacatgtagcagaaagatagatcg


                             <-SP1->      ←SP1->                -25
seq1     tgtatccgtgttggttcttaggcggggcaaggtggggcggtcaaggggaaggtgacatat
         ||| ||    ||| || | |   |||||| | || ||  | | || |||||  || ||||
seq2     tgtctctaatttgattttgataaggggcaggatgcggaagacgagtggaag--gatatat
                                          <GABP>                -25


seq1     acagcgggagtggtaagcacttcCTTTTCCTGTGGCAGCAGCCGGGCTGAGAGGAGCGTG
         | ||    ||||  |||   ||||||||||||||| ||||||||||  ||||||||||||
seq2     agagtacaagtgacaagtctttcCTTTTCCTGTGGGAGCAGCCGGGTAGAGAGGAGCGTG


seq1     GCTGTCTCCTCTCTCCGCCATGgtgagtggaagttctcttggctggccttaccccgtatc
         ||  ||||||||| ||| |||||||||||||| ||  |||   |||| ||| ||||  ||
seq2     GCCTTCTCCTCTCCCCGTCATGgtgagtggaggtcttctacactgggcttcccccctggtc


        +100
seq1     tcg
         | |
seq2     tgg
        +100
```

```
seq1 = human rpL5
seq2 = mouse rpL5

61.91% identity.

      -200
seq1     taa------tcctccc-------------tgctcctcctcaccgcacggatcctgtgaga
          ||      ||||| |             | | | | |||| ||| |  |||||
seq2     aaaaaaaagtcctcgcaaaaagcctctatttccctttctcatcgccc--atcct------
      -200

                                                        -110      ←--
seq1     taccccgagcattggaacctggagagcttgggcaggcattcaccctccttccagcagggg
           ||  |  | |||  |   |||        |  | |||||| |||||| || |||||||
seq2     --ccatgctcgttgagaggagga-------agatgacattcatcctcctaccggcagggg
                                                               -100

         SP1>
seq1     gcggaacgtaaaacgccttgtggccgacgccctctctctttcacacgtcactggcgtgac
         |||  |       |  |||| |||| ||  |  | ||||||| |||||| || ||||| |
seq2     gcgatagcccgcgcatcttgaggcctacagc--cactctttctcacgtcgcttgcgtg-c


                     -25
seq1     cgtccgcgctacatactgcgcctgcgcaaggg-ctgtggcCCTTTTCCCACCCCCTAGCG
          |   | |||||||||||||| |||| | | | ||  || | |||||   |||| |||||
seq2     tggtggtgctacatactgcgcatgcgtacgtgactccggtCTTTTTC---CCCCTTAGCG


                                                                --YY1-
seq1     CCGCTGGGCCTGCAGGTCTCTGTCGAGCAGCGGACGCCGGTCTCTGTTCCGCAGGATGgt
         | || |   ||||||||||  || |||| ||||  || ||||||| ||||||||||||||
seq2     CGGCCGACTCTGCAGGTCTGCGTGGAGCCGCGGTTGCGGGTCTCTATTCCGCAGGATGgt


                            +100
seq1     gagtggatgcctcggtctc---g
         ||||||    || ||||||
seq2     gagtgggcatctgggtctcaggc
                            +100
```

Note: Sequence blocks at -100 and -20 conserved in chicken

```
seq1 = human rpL6
seq2 = mouse rpL6

62.11% identity.

      -200
seq1     ctgccaacctcagaggatcctcctgcctcggcctcccaaagggctgggattaca------
           |  ||    || |  |   | |||   | | || ||    ||||   ||||
seq2     aggagaa----agcgcttttacttgctcagccatctca----gctgcccttacctattag
      -200


seq1     ----ggcgtgagccaccgcacccggcctagga-----tttactacttttttcttaacggg
             || |||             ||||| ||      ||  |||  |   |    ||||
seq2     ggtgggggtg------------gggcctggggggacgttccctaggtgcattgccacgga


                                                      <GABP>
seq1     g-----aaatactacattccccatgatgccccagatccggaaggaagtgagactcg-cac
         |     ||| |||||||| |||||||||||||| | | ||||||||||||   | | |||
seq2     gtaaagaaaaactacatttcccatgatgccccata-caggaaggaagtgatcttggtcac


                       -25     -GABP-
seq1     ttcccatgattgcttatagaccggaagccgggaccttaattCTCTTTCCCATCTTGCAAG
          |||| | ||||||||||||||||||||||   ||||| ||||||||||| |||||||||
seq2     ctcccgtcattgcttatagaccggaagccgctgccttagttCTCTTTCCCGTCTTGCAAG
                       -25

                                                           +70 --YY1--
seq1     gtaagaatcgcgggcctgtctgcaagacttgagttcaggactcccaatccttcgccatcc
         ||| |||| |||||| | | ||    |   |  ||| |||| |||||||||   ||||||
seq2     gtaggaatggcgggcgtctgtgtggcagcggctttcgggac-cccaatcctcgtccatcc
                                                           +70

                           +100
seq1     gaacctggagggcatccagt-c
         |||  ||| || ||||| |
seq2     gaaattgggggacatccgggga
                           +100
```

```
seq1 = human rpL7
seq2 = mouse rpL7

68.7% identity.

      -200
seq1     taaaaagtctaatcaaatgcatatattttcttcaaacaagttttacccgaaagtttgttg
             | ||   ||  | |||||   || | |||  |  |||||||||  |||||| | ||
seq2     ----atgt---attcagtgcat---ttgttttccgatgagttttaccataaagttggctg
          -200


seq1     aaagaggctaaatcagggagttc--tttgcgtctgcaaggcggct---ttttagttctgg
            | | ||||  || |  ||||  |||||| | |||| | |      | | | |||| |
seq2     gccgtgactaagccaagctgttcattttgcgccagcaaagtgcaaaaatctcaattctag


              -GABP-
seq1     attcttttccggcttattctaagacacctatgagtcgtggggcttctctcgcgaagtc-t
         ||| |||||||||||||   | |||| |||| || ||||||||||| | | |  || | |
seq2     attattttccggcttatctcaggacagctataaggcgtggggcttcgcgccctgagacct
                -74

        -25               --Nrf1--
seq1     ttaagtggacagtacgcatgcgccaa----CTTCCTCTTTTTCCGGCTGGAACCATGGAG
         |||||  |||||| |||||||||||     ||  ||  ||||||||||||||||||||||
seq2     ttaaggagacagtgcgcatgcgccacctttCTCTCTTCTTTTCCGGCTGGAACCATGGAG
          -27

                                                  --YY1--
seq1     GGTGTAGAgtgagttgcttctggtcctcttgagaatccttctccatctccagagccagat
         | |||   || ||| | ||||  ||  |   ||||||||||||||||  || | |  ||| 
seq2     GCTGTTCCgtaagtcgattcttctctcccccagaatccttctccatcccccaaactagag


               +100
seq1     actttttgtg
          | ||||||
seq2     gcattttgtc
               +100
```

```
seq1 = human rpL7a
seq2 = mouse rpL7a

51.29% identity.

      -200                               --SP1-                 <-SP1->
seq1     cgg------------------cctggccctcccgcc-----gcagtctctcttccccgcc
           |                  |  ||||| ||||||     | |            |||
seq2     tcggagcctgcggtccggtgccagggcccgcccgcctggacgaa------------cgcg
     -200

                                                     <-SP1->-110
seq1     gcgccgcggtccgaaaacctagtcagccgccgcagcctctcagccccgcctcgattttta
           ||||||| |||| |              |||  || ||||| | |  | |   | | |
seq2     cagccgcggcccgaca--------------cgcccccgctcaggctccgcccctctgtca
                                                        <-SP1->

                                           -70                  BoxAfactor
seq1     gctttataggaatgc---tgttgctttaaatccgaaatcccgtgccggtatcaactctcg
         |  |   | |   |    ||| |||||||||||||||||||| |  ||   |  ||||||
seq2     gagtccgaagtgggtccttgtcgctttaaatccgaaatcccgcgagggcgcctgctctcg
                                           -70                  ******

         ---                 -25        BoxBfactor
seq1     cgatctccgaggccgcatacatattacccacaattccctttcctttCTCTCTCCTCCCGC
         |||| || | |||| | ||| |||  ||||||||||||| | |||||| |||||  | ||
seq2     cgatatctgcggccacttacttatcccccacaattccctcttctttCTTTCTCCAGCAGC
         ***                 -25

                --YY1-
seq1     CGCCCAAGATGgtgagtgagctgtagttccgtggcactatagccaggttccggctgtatc
         ||  ||||||||||||||                     |  |  ||  || ||  ||||
seq2     CGAGCAAGATGgtgagtg---------------------tctctgggcccccgcgctatc


             <-YY1>                                  +100
seq1     cgctgccatcctcctccaggcgc---------------------ggc
         |||  |||||| || || | |||                     | |
seq2     cgcctccatccgccgcccgtcgcccgtcagcggcgttctccgccgtc
                                                     +100
```

Note: BoxA is conserved in chicken, but not in Fugu

```
seq1 = human rpL8
seq2 = mouse rpL8

50.14% identity.

      -200
seq1     cacaccaggacaccttcagccgcgtctcctg-gtgcgcctgcgcgggggccctggaggag
           |   | ||| | |    | |  ||| | | ||  ||||         | |   ||||
seq2     --ccgtaagactcttgagtcggtttcttcagagtctgcct---------ctccccaggac
        -200

                                                 <-SP1->
seq1     gagaagt-------------------------------aggcggggccggcggccgctgg
         || ||||                               || |      | |  | ||  |
seq2     gacaagtttcagcaattgccttggttcagcaatttggtagacacacaggacacctgcatg


         --AP1--                                          <GABP>
seq1     tgactacaagccccggcgtgcaccgcgctaggctggccgtggcgcgcccggaaggtgtcg
         |||||||||| |||| |  |||  ||||| |                  |||||| |  |
seq2     tgactacaagacccgacaggcattgcgctgg------------------ggaaggggcgg
                                                               ←-SP1→

                   ---SP1---GABP- -25
seq1     cggccctgggccccgccccggaagataaggccgctcgctgacgccg-tgtttcCTCTTTC
          |      ||||||||||||||||||||||| |  || |||| |   | |||||||||||
seq2     ag--ttatggccccgccccggaagataaggctg--cgttgacccgtctatttcCTCTTTC
                                 -25

                                                            +50
seq1     GGCCGCGCTGGTGAACAGgtaggtcatccttgcggccttgcggcatgggcagt-atccgc
         ||||  ||| |    |||||| ||   | ||     |  |||||  |  | | |||| |
seq2     GGCCTTGCTTGCCGGCAGgtaagttgtgctctgtccgctgcgggttttgaaattatccac
                                                            +50

         --YY1--                               +100
seq1     cgccatcctcttccgtga-------ggcgcgctgagacccg
         |||||||| |  |||| |       ||||  ||
seq2     cgccatccgccgccgttaactacttggcggtct--------
                                      +100
```

```
seq1 = human rpL9
seq2 = mouse rpL9

57.01% identity.

      -200
seq1     cctctttaggactagcgcagtgcaggggttgagggatcgctaactcccgggaaaggacag
                                ||||  | |||||| |    || ||||| |  ||||
seq2     -----------------------agggatcgagggaac-agcacccccggcaccggacgc
                             -200


seq1     gtcgaa-attacgtcgctccatttatgacaagcagc----------------------ca
         ||| || |   |||||||  | | |||||  |   |                      |
seq2     gtccaacaactcgtcgcttgagtcatgacgcgatacgtcgtcgccgaccggcgcgactcg


                                                 -60   ATF/CREB
seq1     agctgctccacgattggctgctttagccttacgtgcgtcgcgcac-tcatgacgcaatac
         |   ||||  | || |  |  ||  ||||||    |||||| |   || |||||| | ||
seq2     actagctcggcaatcgagtagttccgccttaaagccgtcgctcgggtcgtgacgcgacac
                                                -60

                  -25         ATF/CREB
seq1     gcaggccccttacgcgatacaagtacgtaatgacgacagacgttCTTTCTTTGCTGCGTC
         ||||| |||| |||   |||||| |||| ||||||| ||||||||| |||||||  | ||
seq2     gcagggccctgacggattacaagaacgtgatgacgaaagacgttCTCTCTTTGCCCCATC
                  -25

                                                           <YY1→
seq1     TACTGCGAGgtaaggatgtttctgtgctcgtgggggtactgggaatctgaaaccatctgc
         ||||||||||| ||||  ||   |  |    ||| |  | ||||||||||| |||||  |
seq2     TACTGCGAGgtgaggagcttagggacc----gggtgccccgggaatctgaacccatcctc


                             +100
seq1     tct--gggaaaggctgcggcgcgg--ct
         | |  ||| |   | |||||  ||  |
seq2     tgtttggggaccccggcggcatggagcc
                             +100
```

```
seq1 = human rpL10
seq2 = mouse rpL10

60.38% identity.

      -200    ←SP1→
seq1     gctggccgcccgcggccctggtacccggtcacctctctgatctgcgcatgtgctgggcta
             || |  | |    | |||| || ||||||  |  | |||| || |  | | ||
seq2     ----gcagttccctattccggtatccagtcaccaataagctctgtgc-ttcgttcggtcc
          -200                                                      <-


seq1     cgcccgggcgcaaggccaagagcggc--tgcgtctatggtcatgacgtctgacagagcgt
         |||| ||     | ||  ||||| ||  || |||||| || |  ||||||| |||||||
seq2     cgcctggtacacatgcgcagagcagcgctgcatctatgatcttagcgtctgatagagcgt
         SP1>


seq1     ccacccgtcttcgacagg-----actctatggttcttacgcgcgcagacagaccgcctat
         | || |  |  |  | ||     ||||||||||| ||||    | ||| || ||||||||
seq2     cgacac--caccccctggctgtaactctatggttgttacagtggaagataggccgcctat


         -25
seq1     ataagccatgcgcaggcggaggagcgcCTCTTTCCCTTCGGTGTGgtgagtaagcgcagt
         ||||| | || |||||||  |||||| |||||| |||  || | ||||||| ||  | ||
seq2     ataaggcgtgagcaggcgtgggagcgtCTCTTTTCCTCTGGCGCGgtgagtgagttcggt
         -25

seq1     tgtcgtctcttgcggtgccgttgctggttctcacaccttttaggtctgttct----cgtc
             || | || || ||   | |    || ||      ||| ||  | || |    | |
seq2     cacggttttttacgatggtctcgtaatttttc------tttgggattttttttacactta
                      <-YY1>

                  +100
seq1     ttccgttccg--a
         ||| ||  |   |
seq2     ttctgtgtctgta
                  +100
```

```
seq1 = human rpL10a
seq2 = mouse rpL10a

54.92% identity.

      -200
seq1     ggaccgcggg----gcgct----------caggactgcgacaactctgtgggttaccgcg
             | |||     || ||          |||||||||       |||  | |    | |
seq2     ----cccggcttcagcccttgtcccaatgcaggactgc-------ctgctgctcctagag
          -200


seq1     ccggcctagcggcccagcgacg---------ccccccagcggccagagaaggccgcgctt
         | |  ||  |||  ||||  ||         |||||||||||||  | |  || |||| |
seq2     cggttctcacggtgcagccgcgtggtggcgcccccccagcggccgcaaactgcagcgcct


                                                              --BoxA-
seq1     ctcggggttggcccacccaaccgctctgcgggtaggaccgaggagcaaagcattatcgcg
         ||| || |||||  | |             || | |  |  | | |     | | |||||
seq2     ctccggcttggctgagc-----------gcggaacgtgcccgtaacgggcaactctcgcg


         --               -25    --Nrf1--
seq1     agatcagggccactatttgagcgcatgcgcaagacatgctagtCTCTTTTCCGGTTAGCG
         ||| |  || | ||||||||||||||||||||  |   || | ||  |||||||||  ||
seq2     agaacgcgggctctatttgagcgcatgcgcaaagcgctct-gcCTTCTTTCCGGTTTCCG
                       -25


seq1     CGGCGTGAGAAGCCATGAGgtgagttggt--cctgaaagccctatccgcgttcatccg--
         ||||    | |||||||||||| ||||| |  | ||   || ||||||||   ||||||
seq2     CGGCAGCCGCAGCCATGAGgtaagttgttatcgtg---gcgctatccgccgccatccgtg
                                                          ←YY1>

                                       +100
seq1     --------cgccttcaggtgccccgccgagggttc
                 ||||  | |  ||||||         |
seq2     cacccacacgcccgcggacgccccg---------c
                                       +100
```

```
seq1 = human rpL11
seq2 = mouse rpL11

63.66% identity.

      -200
seq1     tcaa---------aaactatttttggaaaacccagatcccggtcttcagccagcgtcgcc
         |  |         | || |   | ||| ||||||   ||  |||||  |||   |  | |
seq2     tggactcaagaacataccagcatcggacaacccacgacctagtcttttgcccaagatgac
      -200


seq1     tgcagtggctagcactacccacaagggagaagccgacgctgcagggctttgccaaatgcg
         ||||||  | || ||   |||| |          || |  |||| ||   | ||  |  |
seq2     tgcagtttcgaggacagtccacta----------gaggtggcagcgccacgacagctatg


                                              -50      --SP1--
seq1     cctctcttcctcgtcttcatctctgtggttagcctgggaaaagagcccgcctcctggccc
         |||||||| | |    ||  |||  | ||  |||| | | || ||||||||||||| |||
seq2     cctctctttccccctctcccctcgtttgtctgcctagaagaaaagcccgcctcctgaccc
                                              -50

         -27            -GABP-
seq1     ataaggcc-ctcggccggaagctccgctttCTCTTCCTGCTCTCCATCATGGCGgtgagt
         |||||||| |  |||||||||   | || |||||||||| |||||| |||||||||||||
seq2     ataaggccacggggccggaagtctccctctCTCTTCCTGTTCTCCACCATGGCGgtgagt
          -27      <--SP1->

                                      +60     --YY1--
seq1     agctgggacctggatttgctttccttt-atccgtcgccatccatggcaggccgagcctgc
         ||  | ||  ||| ||||| |  |||| ||||||| ||||||   |  ||| |||||
seq2     aggcgagagttggctttgcctcgctttcatccgtctccatccgcagtcggcggagcccag
                                     +60

                +100
seq1     gggggctactt
         || ||||  |
seq2     ggcggct-gtc
                +100
```

seq1 = human rpL12
seq2 = mouse rpL12

72.81% identity.

```
      -200
seq1     ggagcgaa----ctacccgaacc----ccctttcggaatctctagcttcagcgcaccgcg
           ||  ||    ||  ||||||     ||| ||| ||||| | ||||      ||||
seq2     ccaggcaaggtgct--ccgaacatttaccccttccgaatcccgagct------cacccac
      -200                                <GABP>


seq1     gtgccctgcttgccagccgtcagcctcaggcgcctgcccacaacaaacatggcgacaagg
            ||  |||    ||| | || ||| ||| |||||||||| || ||||| |||||| |||
seq2     agaccaagctctgcagtcctctgccgcagacgcctgcccataagaaacagggcgaccagg


                                          --BoxA--
seq1     gcgtctctggaaggccgggcagtgacgacagttctcgcgatagccgcgttttcctgccta
         | ||| |    || | |||  | | |  | |||||||||| ||| | | ||| | |||||
seq2     gtgtcgcccagagactgggtggggccagcggttctcgcgagagctgagcttttccgcccta
                                                            <GABP>

         -25                  <GABP>
seq1     tatctggcttgtccgc-gcgatttccggcCTCTCGGCTTTCGGCTCGGAGGAGGCCAAGG
         |||| ||| ||  ||  ||    |||| | | ||||||||||||||||||||||| | ||
seq2     tatccggcgtgggcggagcaggatccgtCTTTTCGGCTTTCGGCTCGGAGGAGGCAACGG
         -26       ←SP1>


seq1     TGCAACTTCCTTCGGTCGTCCCGAATCCGGGTTCATCCGACACCAGCCGCCTCCACCATG
         |||||||| |||||||||||||||||||||||||||||||||||||||||||||||||||
seq2     TGCAACTTTCTTCGGTCGTCCCGAATCCGGGTTCATCCGACACCAGCCGCCTCCACCATG
                        +50

              +100
seq1     CCGCCGAAG
         ||||| ||
seq2     CCGCCCAA-
             +100
```

seq1 = human rpL13
seq2 = mouse rpL13

54.51% identity.

```
        -200                                ←--SP1--→
seq1     --gtcggttactcagggcccaggaatcacggccgccccgccgcattct----cagcgg--
           ||  |   || ||  | | |||   ||  || | | ||    ||      |||||
seq2     aggtgcgcccctgagcacgccggagatac--ccacactgcgaagttaggaaccagcgaga
      -200


seq1     -----caacatgtctgcga------cggcggccctagggctgccgggagagccgcggcga
              ||||  | | || |      ||  || ||   ||| | ||||   ||    | ||
seq2     ataaccaacccgacggctagaaaggcgatggtccgccggcgggcgggttcgc----gtga
                                                  ←--SP1--→


seq1     cgtcagttcctcctttcggggctctgattggtcagagcgcccggcgcttctggttggccg
         ||||| ||||   |  |  |  | |||||             |||| ||  | |||||||
seq2     cgtcacttccctttcgcctgattttgatt-------------ggcgtttgagattggccg


                  -28
seq1     gccctgctatcatcccagagtgcattgcggggccgcttcCTTTCCGCTCGGCTGTTTTCC
         | |   |||  | ||||| |||| |||||| |||   ||||||||||||||| ||| |||
seq2     gactccctacaagcccagggtgctttgcggtgcccggtcCTTTCCGCTCGGCCGTTCTCC
                  -28

                                                              --YY1-
seq1     TGCGCAGGAGgtgagggagactgggtcctgg--cctttgggcatcatccagcgccatcgg
         ||   ||||||||||        |   | ||  || | | |   ||||  || ||||||
seq2     TGTATAGGAGgtgagtctctgccgccgccggtcccatcgtggtccatcgggctccatcgc


                          +100
seq1     cctggcgcttcagccaacgcg
         |||    |  | | ||   ||
seq2     cctccatccgctgtca--tcg
                          +100
```

```
seq1 = human rpL13a
seq2 = mouse rpL13a

66.77% identity.

      -200                                     <GABP>
seq1     acagcgc------------tgaccgcggaggtccaaccggaagaatgtccggattggaca
         |   |||            || ||||  ||  |||||       |  | | |||||||||
seq2     atcccgccccgcacagtcatggccgccaagagccaactcaggcgacattctgattggaca
      -200

           <GABP>    --SP1-
seq1     ttcggaagagggcccgccttccctggggaatctctgcgcacgcgcagaacgcttcgacca
              || | ||||||||            ||  |   | ||| |  | |||||||||||
seq2     aaataaaaatggcccgcc------------tcgatagacgcgcactcaccgcttcgacca


                                       -60  -ATF/AP1-    --SP1--
seq1     atgaaaacacaggaagccgtccgcgcaaccgcgttgcgtcacttctgccgcccctgtttc
         || | || || | ||  | || |  | | |    |||||||||||  |||||||||   |
seq2     attagaagacggaaactcttcggttcgagccgtctgcgtcacttcctccgcccctgcagc
                  <GABP>               -60

                                                                -YY1--
seq1     aagggataagaaaccctgcgacaaaacctcctcCTTTTCCAAGCGGCTGCCGAAGATGGC
         |||||||||||||||||||||||| |||||||||||| ||| ||||||||||||||||||
seq2     aagggataagaaaccctgcgacaagacctcctcCTTTCCCAGGCGGCTGCCGAAGATGGC
               -25


seq1     GGAGGTGCAGgtatgggctccgcgcgggccggggcggcaaggggccgggtgggatccagg
         ||||| ||||||  || ||| ||||| ||| ||||||   |||| ||||||||  |||||
seq2     GGAGGGGCAGgttcggactctgcgcgcgcc-gggcggtgtggggtcgggtgggtgccagg


                 +100
seq1     ccggaatggggt-
         | |||  |  ||
seq2     cgggagcgcagtg
                  +100
```

```
seq1 = human rpL14
seq2 = mouse rpL14

57.68% identity.

      -200                                    <-SP1>
seq1     atcaccaaaaa-----------------caatctcgccgcccaactttgccaaaaagcaa
         |  |   | ||                 ||| ||| | || |   | ||   | |||||
seq2     acaaaatagaaaggagaaattacaacgccaagctcactgcacgtgtctgaggagaagcag
      -200


seq1     cttaacgccagccgactccgccatgtttt--cccagtgagcttgcgagcgcgtgcgtacg
            |  |  |||||   |||||||||  |  |||||||||| |||        |   | |
seq2     tccatagagagccggaaccgccatgtcataacccagtgagcatgc--------gtaaaag

                                                                   ---
seq1     cacgcacgcacgcacgcaccaaccaatcggtgctcacgtcggtgactcgttaccgcaggg
         ||||||||    | ||   | |||||||||||  ||||  ||         ||  | |||
seq2     cacgcacgtttccccg--tcgaccaatcggtgggcacgctgg---------acgtcgggg

         -SP1--        -30   --Nrf1--   --SP1--
seq1     gcggtgcgttcttctacacatgcgcagggttgggcgggtcttcttcCTTCTCGCCTAACG
         |||| || |    | ||||||||||| ||  ||||||| | |||  ||||||||  | ||
seq2     gcggggcttctgccgacacatgcgcacggcagggcgggccctctctCTTCTCGCTGAGCG


seq1     CCGCCAACATGgtgagtcttactgttgcgggctccggggccgtcgaccatgccgctcgac
         ||||||||||||||||| |   ||  | | |||||| |  || |   |  | ||| || |
seq2     CCGCCAACATGgtgagtgtgtgtgccgagagctccgagtgcggccgtcgcgacgcccgtc


                        +100
seq1     ctccacctccgctgggaag
          ||  || | |  |||
seq2     ttctgccccgggcggggga
                        +100
```

seq1 = human rpL15
seq2 = mouse rpL15

66.24% identity.

```
        -200                                     ←-Sp1-->
seq1     --ggccgctcagg-------ctcgaatcttgcg-gagcagggggcgggacaatagcggcc
           ||| |||||||       ||   | |||| | |||||   ||         ||||| |
seq2     ttggcagctcaggagcactgctgagagcttgggagagcatccgg--------aagcgg-c
      -200                                        ←---GABP---->   <Sp1

                               --Sp1--                     -100
seq1     gcggcgccccactcggcagaactccgcc-accaggcgcgatgccggaactacatgtccca
         | |  | ||||    ||   |||||||| ||||||   |||| ||| |||||| | ||||
seq2     gggctgtccca----gcctgactccgcccaccaggttggatgtcgggactacaagcccca
         -→

                                      -GABP-    -50
seq1     tgacgctctgggaggccgcagctttccaccggaaagagggtggctgaggtggg-ggagga
          || || | | | |||  ||     ||||||||| |||||| || | |||||| ||||||
seq2     agaggcacagtgcggctacaatgccccaccggaaggagggtcgccggggtgggaggagga

               -25                                          --YY1--
seq1     gcc-caaaaggcattgtgggagtacagctctttCCTTTCCGTCTGGCGGCAGCCATCAGg
         ||| ||||||| |||||||||| |   ||  ||||||||| |||||||||||||||||||
seq2     gccacaaaagggattgtgggagaaagccttcttCCTTTCC-TCTGGCGGCAGCCATCAGg
                                                             +20

                                                                 -YY1-
seq1     taggctgcgttgaggatctttgctcttccatccgcctttgatcgtcttcctcttcagcca
         ||||  |    |  | | ||||    || ||||| |    ||||   |  ||||||||||
seq2     tagggcggcggggtggtgtttgtgtgtctatccgtcgcgaatcgcgctagtcttcagcca
                                                             +80

         --        +100
seq1     tccaggtccgg-gg
         |||    |||| |
seq2     tccgaccccggcga
         --        +100
```

```
seq1 = human rpL17
seq2 = mouse rpL17

69.25% identity.

      -200
seq1     cctgaattttatttctgtat-----acaaaaaagagaactttgagacagtccctaagta
           |  ||||| ||||| | ||     | |||||| | ||     ||| ||| |||||  |
seq2     -atacattttaatttcggaattaaaaataaaaaataaaa----aagaaagttcctaaaga
       -200
                                                      -100    <GABP>
seq1     ggca---attcgttttcacacgttcttaaaacatattccacactcccttcgctttccgcc
          |||   | || |||||| || |||| ||| |  |||||||||| ||||| |||  || |
seq2     cgcacgcactccttttcaaactttctaaaagc--attccacacttccttc-cttcgcggc
                                                                     <

                                               --Box A--
seq1     ttcctgcctcctcagatctcgtttcttcggctacgaatctcgcgagaagtcaagttctca
            |   |||||||||||||||| ||| ||||   | |||||||||| ||| || |||||
seq2     ccgcccactcctcagatctcgttccttgggctggaagtctcgcgagaggtcgaggtctca
         -Sp1->

          -25
seq1     tgagttctcccaaaatccaccgctcttcCTCTTTCCCTAAGCAGCCTGAGGTGAgtgttt
         ||| ||||||||  | ||  ||| ||||||||| |  ||||| ||| |||||||||| |
seq2     tgaattctcccagtagccttcgcgcttcCTCTTCCTGTAAGCGGCCAGAGGTGAgtgcta


                                    +60    --YY1--  <-YY1>
seq1     ctcctgcgttgctc-cgagggcccaatcctcctgccatcgccgccatcctggcttcgggg
            | |||  |  | |  ||| | |||||||| |||||||  |   || ||||| ||||
seq2     actcggcgccgggctctcggggcgaatcctcc-gccatcgttgttgtcgtggctccggga


              +100
seq1     gcgccggcc
         |||| ||
seq2     gcgctgggg
              +100
```

```
seq1 = human rpL18
seq2 = mouse rpL18

51.27% identity.

      -200
seq1     cccgacgtgctggcccttccctcttggacgttgcgcttgttcctgcgctctatgctctct
                        |||   |  |||| |   || ||
seq2     ---------------ctttggttctggaagcctcggtt----------------------
                     -200


seq1     gccgttatcgcccggctagtcagtcgtccaactcaccacagagaagtccggatcgtggta
                         || || ||| ||| ||  ||||  |||||  ||  || ||||||
seq2     ----------------tactcggtcctcccacataccatcgagaaacccaaattgtggta


                           ATF/CREB/AP1  -45
seq1     gagcgccgcgtcgcacccatgtgacgtcacggcggcgccac-------------------
         |||| ||||||||| | |||||||||||||    || ||||
seq2     gagctccgcgtcgcgctcatgtgacgtcacccgtgcaccacagagacgccagttccggat
                                      -100

                                                         ---SP1--
seq1     -------------------------------tcgcttgaggct--ttccccgcccacccc
                                        ||||  |  |||  |||||||||||| ||
seq2     agagtgtcccgcattgcgtcacgagtgcgtctcgcaggccgctctttccccgcccactcc
                                                        -25

                                                 --YY1--
seq1     agcccg-ttctctCTTTCCGGACCTGGCCGAGCAGGAGGCGCCATCATGgtgagctcgct
          || || |||  ||||||||||||||||||||||||||||||||||||||| ||||||||
seq2     ggcgcggttccgtCTTTCCGGACCTGGCCGAGCAGGAGGCGCCATCATGgtaagctcgct
                                                  +30

                                       --YY1--             +100
seq1     ttgcggcgttcggggctggataatccgctgccatccgccgtggttttgggcta
           ||||  | |  |  |||   |||||||||||||| | |    |   |
seq2     gagcggtctccccgagtggcccatccgctgccatcctcagcaagtcccgctcg
                                        +80                +100
```

Note: SP1 site in Xenopus ortholog, but not aligned

```
seq1 = human rpL18a
seq2 = mouse rpL18a

66.66% identity.

      -200
seq1     gga-----gcttgcctcgttgccttctgggaaacgaagtctcgctgaagagaggcggaac
                 ||  | | || |||  ||||||||  | ||||||  ||||||||||||| ||
seq2     ttgcaaaggcccgtcgcgctgctctctgggaactgtagtctccgtgaagagaggcgggac
      -200                                                    <-SP1>


seq1     tttga-gtagcaggtggatccatagccaatgaagacgcgggtcgctttgagtgcggggca
         ||  | |||||     ||||  || ||||| || | ||      ||| ||  ||
seq2     ttatacgtagc----tgatcggtacccaataaaaaagccaagaacttagaccgctactag


                                       --SP1-- -GABP-
seq1     gagaagccaatggacgtgcggcattgagagggcgggtcttccggtagtgaaggcctggtg
           | | |||||||| |  | ||||   ||||||||| ||||||  || ||   | |||||
seq2     ctgtacccaatggaagcaccgcatcaggagggcgggacttccgtcagaga--ccatggtg
                                        -50

        -25
seq1     aacggctgcgcgacagaggacacttcCTTTTGCGGGTGGCGGCGAACGCGGAGAGCAC--
         |  | |||||||| |||  ||||||||||||| |  |||||| ||||   ||| || |
seq2     agagcctgcgcgaaagacaacacttcCTTTTGTGACTGGCGGTGAAC---GAGTGCGCAG


seq1     -GCCATGAAGGCCTCGGGCACGgtaaggcgggcgcggcgcggcagggggccaagggatgg
          ||||||||||||||||||||||||||| || ||||||| ||||   ||    | | |||
seq2     TGCCATGAAGGCCTCGGGCACGgtaaggaggccgcggcggggcaaaaggtgcggcgttgg


              +100
seq1     ggcacgggc
          |   ||||
seq2     cggcagggc
              +100
```

seq1 = human rpL19
seq2 = mouse rpL19

64.86% identity.

```
      -300                -GABP-
seq1     ctgcgcaatccagctgacttccggttac--agataaccacgtgattgggaacccttgctg
         |     | |     |||||||||| |||  |||  |||||||||       ||||  |||
seq2     c----gacttagcgtgacttccggctacccagaggaccacgtgaccaaacgccctcactg
      -300

                                            -205  <GABP>
seq1     cgcatgtctagtaggaagtcggactataccacttt-ccctacggaaggggtactt-tttt
         |||||||       | |||||||||  |||| ||| ||| ||| ||||||||  | ||||
seq2     cgcatgtgcgccgcggagtcggactgaaccattttgcccgacgaaaggggtaggtctttt
                                                  -200
                                        -150
seq1     atgtttttaagtttaaaaccgatttctgatatttgacttttatcatttcaggcctatatg
          ||||||  |||||||||| ||||   ||| |      | ||  ||  |     | || |
seq2     gtgttttacagtttaaaactgattctcgatctccagtatctaggatcacgattttttagg
                                        -150


seq1     gaggctatgagtgagtttagtgtggcagaagatgaaagaaccggacaggaatacggacga
          ||   |  | | ||| |    ||  | ||| ||||| | |    |   ||||| ||| |
seq2     aagaaaacaaattagtgtgagatgaaataaggtgaaatagcgtagcgtaaatacagacaa


                                               -25
seq1     aattggagcagggtttgggctctccccttcgcagataatgggaggagccgggcccgagcg
            |||||  ||| |||||||||||||||||||||||||||| |||||| |   |   |
seq2     tcatggagtggggcttgggctctccccttcgcagataatgggcggagcctgcggcccaca
                                              -26<-SP1>


seq1     agctCTTTCCTTTCGCTGCTGCGGCCGCAGCCATGAGgtgagggcgagctggtctccatc
         |||||||||||||||||||||||||||||||||||||||||| | |  | | ||||  |
seq2     agctCTTTCCTTTCGCTGCTGCGGCCGCAGCCATGAGgtgagcgggcccggctctcggtt


                                                     +100
seq1     aggcgctgacgcgtgtcgacaagggactgtcggtcttggga---ccg
             || || | | ||||||  ||| | | |||    || |   |
seq2     cttggccgatgag-gtcgac--ggggccgacggggccggcaaggctt
                                                     +100
```

seq1 = human rpL21
seq2 = mouse rpL21

61.68% identity.

```
      -200
seq1     tga--------cacgatgcaagtgtcagcttgtgttggccggttgcctttctttcgcaag
          |         || |||||  | || || || |||||||| | |||||||  |  ||
seq2     gggatgtctagcaggatgcctgagtgagtttctgttggcctgctgcctttaatctgc-gc
      -200


seq1     aggggaaaatatcctcacagactttctgtgttctcagagacgatcttcttccctagaaag
          |||||   | ||||||| |  | ||   ||| || | ||||||    ||| || |  |
seq2     cggggagcgtgtcctcacggcgtctccaggttatc-gggacgatgaggttctctcg--ac


seq1     aagtcgcttacggttggcgtttccct-caggcagcgccgtgggcagcccacaggcccttg
          || | |    |   |||||||| || ||||||||  || ||||||||| | || |||||
seq2     cagcctcgcgggcccggcgtttctcttcaggcagcttcgcgggcagccctc-gggccttg

       -29               --SP1--                    --YY1--      +30
seq1     taag-gcgcgcgccgcggccccgcctctttCCTTTCGGCCGGAACCGCCATCTTCCAGgt
         |||| |||     ||| ||||||||    ||||||||||||   | ||||||||||||||
seq2     taagagcg-----cgcagccccgccctcctCCTTTCGGCCGCCGCAGCCATCTTCCAGgt
       -25                                                         +30

seq1     agggcctacgcgtggctcccgggcgctacgtgttcgacagacttgaaccgcgaccttgtg
          ||  |  ||||||||||     | |  | |||||  || ||  |   ||      ||||
seq2     tggtac-gcgcgtggctc----tctcgtcttgttctccacacacgctgcg------tgtg

                          +100
seq1     gc--------ctcagag---g
         ||        | |||||   |
seq2     gcgcccacgccgcagagactg
                          +100
```

seq1 = human rpL22
seq2 = mouse rpL22

61.23% identity.

```
      -200
seq1     gg----------------------gcctccttccttccccagaaaccctccaggcctggg
                                 | ||||| |||| |||   | ||  |   |||    
seq2     cattcgcagtgggacagcccgtctgactcctccctttcccgacagccgacttcgcc----
      -200


seq1     agaggacgcgttttgcattcaggaaaaaccaatcgctcccctatcgt-acataaccaaga
                            | || |||| |||| |||  || ||||| || ||      |
seq2     -------------------ctgggaaaagcaatagctggccgatcgttacttaggtggca


seq1     gaaaaggtggcggacgaggggcgtgg-caaagtgattagtgcggcctggaccct-gcgtg
         |  |||  ||||  ||||  ||  || |  |  ||||   |  ||||||  ||| |||||
seq2     gggaag--ggcgatcgagaagcaagggctgatcgattgaagttgcctggctccttgcgtg


                                    -25
seq1     ccttgcgcgctgcgggctctttgcgtctgcgtagttcgctcacctCCCTTTCTAACTCCG
         || || |||||||||||||||  ||| ||||||||| ||| | |||||||||||  |||| 
seq2     ccctgtgcgctgcgggctcttagcgcctgcgtagtgcgcccccctCCCTTTCTGCCTCCT
                                    -25


seq1     CTGCCGCCATGGCTCCTGTGgttagtatggctccgcgtgaggcctcggctccaggggagg
         ||||||||||||| ||||||||||||||||||||||||| |||| ||| |||  | | ||
seq2     CTGCCGCCATGGCGCCTGTGgttagtatggctccgcgtgcggccgcggttccgtgagcgg


                                     +100
seq1     cacgtgggcctcgccgagcccggca
         ||||||||||| |  |       | 
seq2     cacgtgggcctggtgggctggctcg
                                     +100
```

```
seq1 = human rpL23
seq2 = mouse rpL23

63.69% identity.

      -200
seq1     gctcgtagagaag----atacttcctatctcttgcgcttct--acctcaact--cccact
           | |  | | ||    | | || ||  | | | | | |||  | | || ||  ||||
seq2     cgttg-ggtgtagccccaaaattactcgcccctacacatcttaagcgcagctttccca--
      -200


seq1     tcgcagagagggctaaaagcgacgtcgtaaacttattacgtaataaggcagcgcccagag
          ||||||  || |     |||  | |  |  | | |||||| ||   || ||| || | |
seq2     -cgcagaacggtc---ccgcggaggcccaggccttttacgtcatctagctgcgacccggg


          <GABP>                               -50    --SP1---
seq1     gcggaagaggccggttttt---gctccggccacgtgaggagggtgggcggggcgttaaag
         | ||  ||||| || |      | ||||| |||||| |  | |||||||||||  |   |
seq2     gagg--gaggcgggctggacgaggtccggtcacgtg-gctgagtgggcggggc-ctcccg
                 <-SP1>                        -50

             -25
seq1     ttcatatcccagtgtcctttgaatcgacttcCTTTTTTCTTTTTTCCGG-CGTTCAAGAT
         |||||||||||||||||  || |   ||||||||| |     ||||||| ||||||||||
seq2     ttcatatcccagtgtcccctgtactaacttcCTTTCTGACCCTTTCCGGGCGTTCAAGAT
             -25

                                                   <-YY1>
seq1     GTCGAAGCGAGgtgagg--ttttgtttctggaggatcctccaccatctgtcgtgcagtgg
         |||||||||||||||||  |||||  | ||| ||||||     |||||| || ||   ||
seq2     GTCGAAGCGAGgtgaggaattttgagtttgg-ggatccgttggcatctgccgcgc--ggg
                                                                     <

                   +100
seq1     ctggcggattcgtc
          ||||     | ||
seq2     atggcccgcccatc
         YY1->     +100
```

```
seq1 = human rpL23a
seq2 = mouse rpL23a

63.14% identity.

      -200
seq1     a-------ctacaacccccatgaagctttagtgcctttgaggggaggagtggcgcatgtt
         |       | |||   |||| |||||       | ||  | ||||   |||||||||| |
seq2     aaaaaacgccacatttcccaagaagc------cctttcaaagggaaaggtggcgcatgct
      -200


seq1     tgttcacgcacgaaaaagaaattaaacctaactaccgttcccagagggcgccgctctgca
         || || | |||    | | |||||||   ||||||  ||||||    ||  |    | ||
seq2     tggtctcccac-tctagggaattaaatggaactacaattcccaagaagcctcatattaca


                                       --BoxA--
seq1     aattacccaatcagctctaagtacaaagcatcgcgagtctttagtgctctttggcgctat
           | | ||||||||  |||  || ||   |||||||| ||||  ||  | |     ||||
seq2     gttcatccaatcagtgctatatataagctatcgcgagacttttatgagcctccattctat


         -25                                      --YY1--         +30
seq1     aagcccgtgggaacgagcattggagacCCTTTTCA--CAAGATGGCGCCGAAAGCGAAGA
         |||| | ||||||   |  | ||  | ||||||||  |||||||||||||||||||||||
seq2     aagctcatgggaattgggctgggccatCCTTTTCAGCCAAGATGGCGCCGAAAGCGAAGA


seq1     AGGAAGgtgtgtgttggtgatgggccgcag-ctggtttaccggggattccgcgccgcaga
         |||||||| |||   ||| |||||   | | | |||    |  |||| |    |||||||
seq2     AGGAAGgtatgttgcggt-atgggaatctgtcaggtcggtcttggatac----ccgcaga


                 +100
seq1     gcgaac--gaat
         ||||||  |
seq2     gcgaactcgcgg
                 +100
```

```
seq1 = human rpL24
seq2 = mouse rpL24

55.89% identity.

      -200
seq1     aggccct-------------ctgggcccgagaac-----cctccagcgc-ctctggatga
            |||              || || ||   |||     |||| | | | |||      |
seq2     gaacccaagccaagaaccaacttggaccttaaacgaacacctcaaacccactccccgcca
      -200                                                      ←SP1-→


seq1     attgcgccagaggaagtcagctgt--cagggaaaacgagatctttcgcctcgcgatgatt
         ||   ||   ||||||  ||||||  |  |||||  ||      | | | | ||    |
seq2     at---gctgtaggaagaaagctgttgctaggaaacaga-----ctgggcgctcggaagtg
                                                              <GABP>

                                                         <GABP>
seq1     acgtcatcaaggagggccaggagcatcgaaggatattg-gggaggagatttccggccccc
         || |||||   | | ||| ||  ||  |||||  |||  ||||||||||
seq2     acatcatctctgggcgccggggtcaaggaagggaatttagggaggagat-----------
              120       130       140       150       160

                        -25                                     -YY1--
seq1     atccccgcagcgaccttataaggtctcttcccatgattctctCTTTCTTTTCGCCATCTT
          || |  |  |  || |||||||  |  |||||||||||| ||||||| ||||||||||
seq2     -tctctccctctgccctataagg--ttctcccatgattctttCTTTCTCTTCGCCATCT-
                      -25


seq1     TTGTCTTTCCGTGG---------AGCTGTCGCCATGAAgtaagcacgacccgtggttccc
              || ||  ||         | || || |||||||||||||  |  ||  |  ||||
seq2     -----TTACCCCGGCAGGCCGACATCTATCACCATGAAgtaagcgggcgcccggtgtccc


                                    +100
seq1     aatccgcttacatcctcgggcagaactcctc
         ||||||| | ||||||  |||  ||    ||
seq2     aatccgcctccatcctgaggccga---gcta
                                    +100
```

seq1 = human rpL26
seq2 = mouse rpL26

63.63% identity.

```
      -200GABP>
seq1     cggaaggaga--------------cactaggacct-----ggaggaggggagttttgcaa
         |       |||             |||    ||||     ||| || |  ||       |
seq2     ccttccccagaggactaagcctaccacgcacacctccgagggatgacgtcag------ga
      -200


seq1     tccctcgcagttcccctcctagccactaggtgacactagccataaatttatttcccagtt
          |||||||||| ||||||| |||| |||| || | |||     || |    |||| ||||
seq2     cccctcgcagtccccctccgagccgctagatgtcgcta-----aactcccattcctagtt


                                                           --BoxA--
seq1     tactcccctcgctcaaactccgcgcccctctcgctccgagagacataggtctcgcgagat
         |  ||   || | ||| |||| | ||||| |||||||||||        |||||||||||
seq2     tcttcttttctcccaacctcctctcccctttcgctccgaga-------ctctcgcgagat


                    -25      -GABP-                        ---YY1---
seq1     ctttggtaaacttacagaaccggaagcagcgtgtagttCTCTTCCCTTTTGCGGCCATCA
         ||   |  ||||||||||||||||||  ||||||||||||||| |||||||||||||||
seq2     ctcgcggcaacttacagaaccggaagtcgcgtgtagttCTCTTTCCTTTTGCGGCCATCG
                    -25                                          +20

                                                         --YY1---
seq1     CCGAAGCGGGAGCGGgtaaggattcggcgggcaagcgggtgtaatcagcagccatccgtt
           | | | | ||| ||| ||   ||||||||    ||   |||||| |||||||||||
seq2     GTGGATC-GCAGCCGgtgagttctcggcgggttgtcgctggtaatccgcagccatccgcg
                                                             +75

                        +100
seq1     cttgggcatggtggctt-c
             |||  || |||||
seq2     gcgaggcgaggaggctttt
                        +100
```

```
seq1 = human rpL27
seq2 = mouse rpL27

65.82% identity.

      -200
seq1     c-------------tcttgtctactgctggagctgctaataaagtttcattcgaatactt
         |             || ||||| ||||| | ||||||||||||| ||  ||| || ||
seq2     ccggaacgctctgttcctgtctgctgctcgtgctgctaataaagcttggttcaaaaac--
      -200

                                            -GABP-
seq1     gagctgccgcgccctgcgtttccctcatgagcttcttccgcccgcaacacgagactcagc
              || | |||| |    ||||| | ||||| ||||||||  ||||       | | |
seq2     -----gcagtgccc-gaccctccctgacgagctccttccgccaacaac-----tttgacc


                                       --SP1--  -GABP-       -40
seq1     accacacaatcagcgtggcaaagcctgaagggcgggacaggaagtgaggtcactcctcac
         ||||  |     || ||||   |||||   ||||||||||||||||| | |||  |||
seq2     accaaccgtggggcatggctgggcctgggaggcgggacaggaagtgaagccacctctccg
                                        -60                  -40

               -25        -GABP-
seq1     cggggtgaaaggttagcggaagtgtccttctttCCTTTTTGCTGGTAGGGCCGGGTGGTT
            ||||||||||||||||||||| ||| ||||||||| ||||||  | ||||| ||  |
seq2     taaggtgaaaggttagcggaagtgccctcctttCCTTTCTGCTGG--GTGCCGGCTGCCT
               -25

                                       +60          --YY1--
seq1     GCTGCCGgtaagtagaagcttgggttgaatctttcaatccgctgccatccgcggcttggg
         |||| |||| |||| | || |||   |  |||| |||||||| ||||| |||||    ||
seq2     GCTGTCGgtgagtaaaggcctggagagggtcttgcaatccgccgccat-cgcggtcccgg
                                          +60

                  +100
seq1     ggtcgaaggtccg---
         |||||     | |
seq2     ggtcgggttgctgggc
                     +100
```

seq1 = human rpL27a
seq2 = mouse rpL27a

55.62% identity.

```
      -200
seq1     cgagcctcacc-----------------gaaaccgtacac---cgccaccaggacactcc
          | ||   ||                  |   |||    |   ||||||| |  ||| ||
seq2     ggcgctcgactagggaggagctaccggggcttccgggggcgggcgccaccggttcacccc
      -200                             <GABP><-SP1-->             <SP1


seq1     gtgatgggggatcaccaccctcagaaagaggaagcgactagcaggcgcgcaatcccgcga
         |             ||| || ||   | |||||| |    || |||||      ||| ||
seq2     g------------cccatccacacggaaaggaagag----gcgggcgccgggagccgtga
         -            -→        <GABP>       <   -SP1>

                   ←-SP1-→                     -GABP-     -50     -SP1
seq1     gaccaggaggccccgcccgaagcccggcctctgtgaccggaagtgaggcgttttgccccg
            ||||| ||||  ||||||| ||||||||  || |||||| ||| ||  || | ||||
seq2     cgtcagga-gcccttcccgaag-ccggcctc-ctggccggaaatgaagc-attggtcccg
                                                        -50

         -----        -25---BoxA--
seq1     cccccgtggccgatacctcgcgagacttggcgaaggccttCCTTTTTCGTCTGGGCTGCC
         |||||| | | | ||||||||||||||| | ||   ||| |||||| | | |  ||  ||
seq2     cccccgcgcctgctacctcgcgagacttcgtgagctcctcCCTTTTCCCTTTCTGCCACC
                    -27


seq1     AACATGgtaggtgtttcgtttcttgcctcctcttccttgccggcggagacccctaagctg
           |||||||||| ||    |  | |||||| || | | || |  ||      ||  ||
seq2     GCCATGgtaggtctt---ctcttggcctccgctccatcgcggagggtcggggctctgc--


                                   +100
seq1     tattcccattgcc---------cctagtc
           ||||||  |||         | |  |
seq2     --ttccca--gcctttcagctgcttgcta
                                   +100
```

seq1 = human rpL28
seq2 = mouse rpL28

55.92% identity.

```
      -200
seq1     acg--------------------gttcccggaatacgctctcggccctctaaccgaagag
         ||                     |  ||  ||  || | |  || |||  |  |||||||
seq2     accccggcgcctcatcacggtacgcaccgcgagaactcgccgggaccttcagacgaagag
      -200

                                            <GABP>
seq1     ttccgaatattcccgagcgttgagcgaattttcggcggaagcagccgaacatcatcgagc
          ||||||  ||||||||     |  || |    ||||||| |             | ||
seq2     ctccgaaggttcccgag-tcaaaaagactcggaggcggaacc-------------caagg

                                     -70      <-SP1->     -GABP--GABP-
seq1     cgcctcggaggtgggga--ccgacaactcctgtcgtgcccgcccggagaggaagcggaaa
         | |   ||| |  |||   ||||         ||  ||  ||||  | |||||||||||||
seq2     cccagaggaagctgggtagccga-------gttcaggcgcgccacgggaggaagcggaaa
                                     -70                 -50

                        -25
seq1     tgcgtgtacggtattaaaggcgtcgcctcgcccctcgccttcCTCTTTCCGTCTCAGGTC
         ||||| | |  | ||||||| |||| | |||| ||||  |  ||||||||||||| || |
seq2     tgcgt-tgccattttaaaggtgtcgtcccgcctctcgtgtctCTCTTTCCGTCTCCGGCC
                        -25        <-SP1>

                                                           -YY1--
seq1     GCCGCTGCGAAGGGAGgtgagcgttcgtcttc-------ctcgcgtggtcgccatctccg
         |||   | ||  ||||||||| ||  | | ||       | ||||||| |||||||||
seq2     GCCATAGGGAGAGGAGgtgagtgtgaggcctctgtccgtcccgcgtggccgccatctc--


                                  +100
seq1     gcccgccgcgcactccctctcagtcctct
               | | |||  ||||    ||||
seq2     -----gctcccacggcctcggtctcctgc
                                  +100
```

```
seq1 = human rpL29
seq2 = mouse rpL29

67.73% identity.

      -200                                                     ←SP1-→
seq1     agacttaagagacaggtcctggagaaacagagaagggaggaggcagcctgaagggggcgg
         | |     | ||  |||       || || |||| | |    |   |   ||| |||||
seq2     aaatagcggggaggggt------taagcaaagaaagaaatctgtgacgctaagagggcgt
      -200

                                                    -GABP--
seq1     aggcaagctcgtagcaccgcctccacgtct--ggcttgcgttcttccgggcttcggaact
          | || ||||     | | ||||| | |    |||||    |||| | ||| | |||||
seq2     ggccaggctc----taacccctcccctttctaggcttcaactctttccggccttggaacc


                                     -60                 -GABP-
seq1     tttcttcgcggagcctg------gggaagcttttccagtagtactttccggcgttgttga
         ||| ||| |   ||| |      ||||||||||||| || | ||||||||||| ||| |
seq2     ttttttctc--cgccagcagtgagggaagcttttcc-gttggactttccggcggtgtcgg
                                   -65   <GABP >

           -25
seq1     ccctatttcccgtgctgcaccgc-agc----ccCTTTCTCTTCCGGTTCTAGGCGCTTCG
         |||||||||||||||||||| ||  ||    | ||||||||||||||    |||||| ||
seq2     ccctatttcccgtgctgcactgcgggcctttctCTTTCTCTTCCGGTCTCCGGCGCTGCG
               -25

                                                     <-YY1>
seq1     GGAGCCGCGGCTTATGgtgagtgttggccgcgcggcatccgatgccatccgcgacttacg
         |||||||||| |||  |||||||||||||   |||||||||||| |||||| ||||   |
seq2     GGAGCCGCGGGTTACCgtgagtgttggccttacggcatccgatgacatccgtgactacag


                  +100
seq1     ggtggcggcgggg
         || |||||||||
seq2     ggcggcggcgggt
                  +100
```

seq1 = human rpL30
seq2 = mouse rpL30

60.12% identity.

```
      -200
seq1     tcccgcctcaacctccga------aagtgctgggattaaaggtatgagcagccgcgtacg
                 ||| | || |      |||  | | |   | | |    |||||
seq2     --------caaacgcccagaaccgaaggccggagcccagacgccgcagcag---------
              -200

                                         ---RFX1--
seq1     gcccccgcagccattctagctagcggtaccaatagcaaccggcagctgccctccgctttt
           ||| ||   | || || |   |||| || |||||||||  |  | ||   |  || |
seq2     --cccagcctgcgttgtaac--ccggtccccatagcaaccaactaccgcagactactctc
                                     -130*********

            <-SP1->                             -GABP--GABP-    -45
seq1     gctccgccccttctgcttgcgatctgt-----------ttccgcttccggtcccgcagtt
         ||     |  ||||||||| || | |            |||||||||||||||| || |
seq2     gcaagcgctgttctgcttgtgacccggaccggcttctcttccgcttccggtcccacaatc
                                                ***********-50

                       -25 Gamma Factor                            YY1
seq1     ccggctctgccgtgaagagctttgcattgtgggaagtctttCCTTTCTCGTTCCCCGGCC
         |    || |||  |||||||||||||||||||||  || ||||||||||| |||||||||
seq2     ctctgtcggcctagaagagctttgcattgtgggagctccttCCTTTCTCGCTCCCCGGCC
                       -25 ***********                             ***

         ---     +30
seq1     ATCTTAGCGGCTGCTGTTGgtgagtgggctcctaccgaccgaggtttaggcagcgcgggg
         ||||| ||||||| |||||||||||| ||||     |    | | |||||| || |||||
seq2     ATCTTGGCGGCTGGTGTTGgtgagtgagctctgcggggtaaacgattaggcggctcgggg
         ***     +30

                          +100
seq1     agct----ttgcgggttgcca
         ||||    | || |||
seq2     agctccgctagctggtgtttg
                          +100
```

```
seq1 = human rpL31
seq2 = mouse rpL31

62.06% identity.

      -200                                                     --SP1--
seq1     gaatccgtcgagaagcgggacc---tcaagctcgc---ggcccaaaagcgcggccccgcc
                 | |||||   |||    || | | |||   || ||      | | || | ||
seq2     ------tgcaagaagactgacaagatcgaccccgccctgggcccgccctgggtccgcccc
            -200                       ←-SP1-→     <-SP1->


seq1     acgacgcctgctagctggccccacttcggcgcgcgccgcttacatcatagctcgcctctc
         | | |||  |||      | || ||   ||||||     ||||  |  |||    |||
seq2     atgccgctcgct-----ccgcccctgacgcgcgctgtatttacgccccagcctaactcct
                          ←SP1-→

                                                     -GABP-    -40
seq1     cccttccctagccacacccc------cttgcctggc---gacccggaagttgtacttgca
           ||||||| |||||||| |      || ||||  |   ||||||||||||||||  ||
seq2     aacttccctcgccacacctcttcctcctcgcctccccgtgacccggaagttgtac-ggct
                                                           -40

                 -25
seq1     actgcggctttccttctcccacaatccttcgcgctCTTCCTTTCCAACTTGGACGCTGCA
         || ||| ||||||  ||||||||| || |||||| |||||||||| |||||| | | |||
seq2     ac-gcgactttcc--ctcccacaaaccctcgcgccCTTCCTTTCCTACTTGGGCCCGGCA
               -25


seq1     GAgtgagtatgggtggcggagtctgggctcctggaatccagccccatcctcctttgg---
         |||||||||||| | |||| |||  |||| ||| ||||| || |||||| |    ||
seq2     GAgtgagtatggct-gcggggtcgcggct-ctgaaatccggcgccatccgcgcccggcgc
                                                            <-YY1>

                        +100
seq1     -gattgtgctaactgggac
          |   | ||   || ||
seq2     gggacgggcaggctcgg--
                      +100
```

```
seq1 = human rpL32
seq2 = mouse rpL32

67.84% identity.

      -200
seq1     aaggataggtagaatacatttctcagtcctattcctagttattattgtctattaaa----
         |||     | | |||| |||  | ||| | | ||  || | ||| |||||| || |
seq2     aag----agaaaaataaatt--ttagttc-actctaagatgttaatgtctactacagaac
      -200


seq1     acatgtatactcagaattttttgcggcattatttttt-gacgtgtctttattttatttaaa
         ||  ||  |||  |  ||||| | | || |||| |  | |   ||||  |||| |||| |
seq2     actagtccactagggtttttttcagtatcatttctcaggcacatcttagttttctttaga


           -80      -GABP-
seq1     agagccggagccggaagtgcttgccttttccctgctaggacccaggggttacgacccat
          || || ||||||||||||||| || ||||| |||| |||||| |  |  | || |||||
seq2     ggacccagagccggaagtgctt-cccttttctctgccaggaccta-agactccgtcccat
             -80    ******

         -25 Gamma Factor
seq1     cagcccttgcgcgccaccgtcccttct--C-TCTTCCTCGGCGCTGCCTACGGAGGTGGC
         ||  ||||||||||| ||| | | |||  | |||||||||||||||||||| ||||||||
seq2     catacctttgcgcgccgccgcctcttcCTTCTTCCTCGGCGCTGCCTAC-GAGGTGGC
           -25**********

         --YY1--          +50
seq1     AGCCATCTCCTTCTCGgtaagtgttaatccgtggcaatccgcattcctgcgggattcatc
          |||||||  ||  |||| ||| |  ||| | ||| ||||||   || ||||  |  |
seq2     TGCCATCTGTTTTACGgtgagtctgtatcggcggccatccgc-cgcccgcggcttcgacg
          ******          +50             <-YY1>
                                          ******

             +100
seq1     tggccccg---
          | |||||
seq2     ggaccccgtct
                +100
```

seq1 = human rpL34
seq2 = mouse rpL34

54.85% identity.

```
      -200                      ---Sp1--      <-Sp1->  -150
seq1     ttgacacagacagctagtgtgaatccccgcccacagaggggcggcacgttggtt--gccg
          |  |       |||||        |||||| || ||   || ||||      |  ||||
seq2     ctacc----ggggctagaaaacgcacccgcctacgga--agccgcactacacatccgccg
      -200

                                   <-Sp1>                    -GABP-
seq1     tacaacgtggtggatccctgcatctccgcccacgtcggagaggtgcgtcggcttccgt-c
           ||||| ||             | ||  ||||   ||      ||||| |||||||| |
seq2     ctcaacgcgg-------------cgccatccacacaggttccccgcgtccgcttccgtac


         -80
seq1     aacacggatacgctctctctgacgca----------------acttcctgtcctgcgcaa
         |||| ||   ||| ||| |||||||                 ||||||||||| ||||
seq2     aacatggcggcgcgctcgctgacgcgccgccttccctgacgtacttcctgtccggcgccg


                    -25
seq1     ttctatttgacctttgaactggcaaaggcttttttcttcCTCTTCCGGGGACGTTGTCTG
         |   | ||||||  ||||||  |     | ||  | ||||||||||||||||| ||
seq2     tcacacttgacccctgaactcacccgccccttccttttcCTCTTCCGGGGACGGTGCTCA


                                                               --YY1--
seq1     CAGgtatggatgttgttctcttttccctgtctttatttccttaccaatcggctgccatcc
          ||||||||  |   |  |||  ||  ||| ||| |  | ||  | ||| |  |||||||
seq2     GAGgtatggtggccttcttctgctctgtgtttttttaccgtttacgatccgtagccatcc


                        +100
seq1     gaggagctgaggaagccta
         |||| |    |   ||
seq2     gaggcgtcaggcgggcggg
                        +100
```

seq1 = human rpL35
seq2 = mouse rpL35

58.89% identity.

```
                        -200
seq1     ------------------acgccgacctcgctatcaggggtgccgtgaatgaggcgagca
                           || | |   || | | | |||  ||   ||     || | |
seq2     ggtgttgctcatcgcgagactcag-tttccccagctgggtcgcttagagaccagccaacc
      -200

                <-SP1->                  ←--SP1--→
seq1     ctac-aaggcggggctccggccctaccaggcggg--gcgggcagtaccacggcgttcctc
          ||  ||||| || || |            ||||  ||||   | ||||||||
seq2     ttaggaaggcaggacttc------------cgggaagcggagggcaccacggc-------

         ←--SP1---→      --SP1--                                    <-
seq1     gggggcgggggagatgaggcggagccggggctcccccgctgcggcgccattggccgcgcc
             ||||  | |  | ||||||| |||| |||||   | ||| ||| |||||||| |||
seq2     ---cgcggctgcgcaggggcggag-cgggcctcccaaaccgcgacgctattggccgtgcc
                  -110

         SP1>              -25            --SP1--
seq1     cgccgccacccggcagtagttgcggaggtcagccccgcctacttcCTCTTTCCCTCGGAG
         || ||||||||||||||||||| ||| ||| ||||||||   |||||||||| |||| |
seq2     cggcgccacccggcagtagttgaggaagtcggccccgcc-ctttcCTCTTTCTCTCGAAC
                                          -10

                                                                 -YY1-
seq1     CGGGCGGCGGCGTTGGCGGCTTGTGCAGCAATGgtgagagctgcgg-aatcgggcgccat
         | ||| ||  |    | |      || | ||||||||| ||||||| ||||||| |||||
seq2     CAGGCTGCTTCAGCCGTGAACAACGCCGAAATGgtgag-gctgcgggaatcgggtgccat
                                                               +70

         -                     +100
seq1     ccgagcgctgtgaggcgcggg----g
         | |    | | ||  || |||    |
seq2     cggtcgccggggacacgtggggacag
                               +100
```

seq1 = human L35a
seq2 = mouse L35a

63.89% identity.

```
        -200
seq1     --gttcccaagtagtacattttgcagcctaccaagatgaaaggactt-aggaaaataatt
           ||||| || || ||| | |||| || ||| |||| |||||| |   ||| ||| ||||
seq2     ttgttcctaaataatacgtgttgccgcttacaaagacgaaagggcaggagggaaagaatt
      -200

                                                            <GABP>
seq1     gtaagagtgctattgaatgtggcttatttcagtgcgaagc-------cgatttccgagga
           ||    | | ||||  | |||||   | ||| |||  |       |  | || ||
seq2     taaa----gttcttgagcgcggcttggctgagt-cgagcctctctctctgtctctgacct


                    -80                        <-Sp1>
seq1     ttgtttttctggcgtttggagaagatgcctaaattgagggcggagtcgaaagatgtttaa
         |||  |||||||||    | | ||  |||||     || ||||||  |   |  |  |
seq2     ttg-ctttctggcgcagtgtg-agcggccta-----agcgcggagaggggcggggcgtgc


                -25       --Sp1--                   --YY1--       +25
seq1     -tcccacaaggccacgcggcggggcctcgtccttCTCTTACCGCCATCTTGGCTCCTGTG
          |||||||||||| |||||||||| || | | | ||||| ||||||||||||| ||||||
seq2     gtcccacaaggccgcgcggcgggg-cttgccttcCTCTTTCCGCCATCTTGGCGCCTGTG


seq1     GAGgtgagtgaagggtctgctgctgaaatttgggggcaaataaccggagtaggtttgttc
         |||||||| |  |   ||||||||||  ||||||| |  ||  | || ||  ||||  |
seq2     GAGgtgaggggggttcctgctgctgagctttggggcccgattgctggggtgcgtttagtt


                  +100
seq1     ctgtgccttg--g
            | |||    |
seq2     tcatccctcaccg
                  +100
```

```
seq1 = human rpL36
seq2 = mouse rpL36

51.36% identity.

      -200
seq1     catagcca--------cctccactggttccgac---------------tccagctcccgg
            ||  |        | || || | |  | ||               |||| ||||
seq2     gtcagagattaaattacttcaacagctaacaacagtgtgaggcagcattccaactcctaa
      -200

                                                                   ←-
seq1     gctcgcaggagtaaacgcgcatgctcagggagctcggtcgcgcatgctcagcgcaacggc
          ||   | | |   |  | || ||  |||   | |||  ||                  |
seq2     cct---aagggatgattcccaagccgaggtctcacgggtgc------------------c


         SP1>
seq1     ggggaagactccagccgccagggggagcgcgtgccgttcttgcctctctggcctgcgcct
         |  |||  |||| ||| |||| ||  || ||   ||  | || |||||| ||| |
seq2     gtagaa-cctccggccaccagaggtcgcccgcagcgcgcctgtctctctagccggaaagg
                                                              <GABP>

                        -25
seq1     cctgagccgagtagatatccggagttccgcgcggcgccagcCTTTCCGCCACGGCCGTC
         ||||      | | ||||||| |  ||| |||||| |||   ||| | | | |||||| |
seq2     cctgcaagtggcatatatcccaggattctgcgcggtgccgttCTTCCTGTCCCGGCCGCC
                        -25                                        ←-

                                                             <-YY1>
seq1     TCTGGAGAGCAGCAGgtaagtggtttcccgcactgccggt----atccgccgccatccgg
            |||||||| |||||||||||      ||   | |||     ||| ||| ||||| ||
seq2     ATCGGAGAGCATCAGgtaagtgg------gcctcgtcggggctcatctgcccccatcggg
         YY1>

                                   +100
seq1     actcccgggtcctctgtgcaggt--tgga
           |||| || ||  || | ||    ||
seq2     catccc-ggccccatggggagtccctgcg
                                   +100
```

```
seq1 = human rpL36a
seq2 = mouse rpL36a

61.7% identity.

      -200--Sp1--                              --Sp1--
seq1     ggggcgggaggaagtcggggtgagtggaccaccctttaggcggagtccttccttaagctc
             ||||||||||| | |  ||  |   ||  |  ||||||||| |    || || |||
seq2     -gggcgggaggaagtggagccgatggctgcagaccctaggcggagcctcggctcaaactc
                                                  -160

         ←Sp1→                          <-Sp1>
seq1     ccgcccgcggctccgaactgagtcctctcagccgcccgagggcgctgcgctgagc--ctt
          || | ||   |   ||  | | || || ||| |   || |||||||| |||||||  | |
seq2     gcgtctgcaacgaaga--taaatcatc-caggcatacgggggcgctgtgctgagctccct
         -140


seq1     aca-----ctctatgattgctcctaccgactcccatgaggaagtgcgatcgggaacctcc
         |||     ||||||| | ||   | || ||  |  |||||||||||   | || ||| ||
seq2     acagacagctctatggtcgccgtttcccacggctctgaggaagtgcacccaggcaccgcc


            -25-GABP-
seq1     tatatacttccgtttgcctcgcggtt------tctttCTTTCCGCGCCGATAGCGCTCAC
         |||| |||||||||      |||| |      ||| |||||||| || | ||||||||
seq2     tataaacttccgtt------gcggctctagcctctctCTTTCCGAGCTGCTAGCGCTCCT


            --YY1--
seq1     GCAAGCATGgtaggacttgctggtgggggccgagtaacatccagcttaatcttt---ccc
         || | ||||||| |  | |   ||||||      || ||||||| |||||| ||   |||
seq2     GCGAACATGgtacg-gtcgtctgtggggctttgatagcatccagtttaatccttgtaccc
             +30

                     +100
seq1     ccccttcgtcgcccgt
           | |  ||    |
seq2     agcgt--gtacagcac
                     +100
```

seq1 = human rpL37
seq2 = mouse rpL37

60.19% identity.

```
      -200                ←-SP1-          ---><GABP>
seq1     ggtcgccagaaaactgggagtcccgcc----------cgcctttccggcactgaaacgcg
         || |||  |  |    |||| | |  |          | || | ||||   || | | |
seq2     ggccgctggggagggcggagccacagcgccaggcacgccccctaccggtgttgcagccca
      -200           <-SP1>


seq1     atcggc--cctgcctggtaccgcatcctcctcttgaccccac--ctacactacgacgacg
         ||||||  |||| ||              |||| | ||      ||||||  |  |  |
seq2     atcggctccctggct--------------ctctcgcccgggagactacacctccgcaccc


                              -60                     -SP1/GABP
seq1     gacgctcgagaacgctgcaccgcgccccgcaggaagtgcttccctgggcggaagcttctg
         || ||  || |  ||||||||  | ||||| ||||||  || ||||||||||||||| |
seq2     gaggcctgatagggctgcaccaagacccgccggaagtagtttcctgggcggaagcttatc
                                  <-SP1/GABP>             -44

                -25  <GABP>            -GABP-
seq1     agcgtgatatagcggaagtgccttctcttccggtCTTTCTGGTCTCGGCCGCAGAAGCGA
           |  ||  | ||||  |||| |||||||||||||| | ||| |||| | | |||||| |
seq2     cacaggaagtggcgggggtgctttctcttccggtCTCTTTGGCCTCGCCGGTAGAAGCAA
                   ←-SP1-→

         -YY1--                                +70   --YY1--
seq1     GATGgtgagttgtgactgtggtgtttgtgaatcgcgttccatcctcgtcctttgtgcctc
         ||||||||||   |  | |||  | ||| |||| |  ||||||||  | | | | ||
seq2     GATGgtgagtgtggtttctgggctctgtaaatctctctccatccttcttcctcgggctgt

                  +100
seq1     tctgtttgctgtgc
          |||  || ||  |
seq2     cctgcctgttgccc
                  +100
```

```
seq1 = human rpL37a
seq2 = mouse rpL37a

51.75% identity.

      -200
seq1     c---cggacatgcttctgc-----tatggttgtttgtcgccatag---------------
             || |  ||| |||||     | ||||| | |||   |  ||
seq2     aaatcgcatctgc-tctgcagaactttggttatgtgtaagctaagaaggatgtgcttcca
      -200


seq1     --------------tttgcgaatctgggtaaacctggatgagagagacgccttacaattg
                       | |||| ||||
seq2     gctcgtgaaccacttctgcgcatct-----------------------------------


seq1     caaacatttctcag--gaggccgcctgaaaacgcccaaaccatagagctcagttcctagc
          |  ||||||||||  ||| | ||    ||||  ||| | ||  ||| ||| |||| ||
seq2     -atgcatttctcagtggagacagcgaataaaccgccagaacaccgagttcacttccgaga
                                                             <GABP>

               -55        -GABP-              -25    --Nrf1--
seq1     taggcctctaccagccacttccgctcgtccgcctaataccgcgcctgcgcaccgcgtctc
          |||      |  | |||||||||||| | | |||| ||||||| ||||||  ||||| |
seq2     gagg--aggtcgcgtcacttccgctcgccggtctaaaaccgcgcatgcgcattgcgtccc
               -55                            -25

seq1     ttcCTTTCTGGGCTCGGACCTAGGTCGCGGCGACATGgtgagtgtgggtctctgtgcggc
         ||||| |  || || || | | |||  ||||||||||||||||||     ||    | ||
seq2     ttcCTCTTCGGACTTGGGCTTGGGTTCCGGCGACATGgtgagtgt---cttcgtaaccgc

                                                +100
seq1     ctagaa---ctctatctgcctgcatctgtccttgttttcttc
          | | |   | |||||   |  |||||| | | || | |  |
seq2     tttggaagtcgctatccatcgtcatctgccttagtctccggc
                                                +100
```

Note: GABP and Nrf1 sites conserved in chicken

```
seq1 = human rpL38
seq2 = mouse rpL38

62.07% identity.

      -200
seq1     agagg-------agggaggcggagg-gagggcggcagccaggatggtccattccattctt
           | |       ||||||| |  || || |  |||||||          |  |||||  |
seq2     gaaaggataagaagggaggtgagggtgatgctggcagcc--------taacgccattgct
      -200

                                            -115
seq1     ----ggagctgcactgttgacttcgaccacaagggggcagggctgctgaaattggaagtg
             | |||||||||  | |  |  |||| ||| |||||||||||||||||||| ||
seq2     ataagaagctgcactactaatctgaaccagaagagggcagggctgctgaaattgaaatga
                                            -111


seq1     cgaacgcctacatgagtcttgatcctcggcaggcaccgtacccagtttcctccgttggga
          |||||    |||   |  || ||||  |||| |||||||| |||||||||| ||||||
seq2     tgaacg----catcctttctggtcctttgcagacaccgtactcagtttcctctgttgggc
                                                                  <Sp1

              -25     -GABP--GABP-
seq1     gggtttaactgcccggaaacggaagtctcgttCTTTTTCGTCCTTTTCCCCGGTTGCTGC
         ||| ||||| | |||||| |||||| |||||||  ||||  | || ||  ||||| ||
seq2     ggggttaaccgtccggaagcggaagcctcgttC--TTTCCCCGTTCTCTTCGGTT-CTCA
         --→ -25


seq1     TTGCTgtgagtgtctctagggtgatacgtgggtgagaaaggt-----aatctggggcaat
         | |||||||||         ||| |  |  |||| | ||||      |||| ||||| ||
seq2     TCGCTgtgagt---------gtgctgggcaggtggggaagggcgttcaatccggggcgat


                                 +100
seq1     ccactgccaggt----------gaaat
         || | ||| ||           ||
seq2     ccgcagccggggagggcgcaggga---
                              +100
```

```
seq1 = human rpL39
seq2 = mouse rpL39

60.74% identity.

      -200
seq1     gcccctagcacactgccgggcccctggggaacgcacttagggaagcgaagcaaagctaa
          |||  |||||| ||  |  | ||||  ||||  ||   | ||| ||| ||       ||
seq2     -ccctcagcacaatgatgatcacctgcaggaa--caggcatggacgcggaggcctcggaa
      .-200


seq1     ctgcatttggcagaaaccagcgcgctttcccacggcgccacgggagcgcaccgctagaca
          | | ||    | ||||| |   |  |||||||||| ||| |||||||||  | | ||
seq2     ttacgttctctataaaccggtttgggttcccacggccccatgggagcgcaggggtggagc


                                                                  <SP1
seq1     gagaaggcactagaagaggcggtcggtgcatagcgtca-------cctcctaaactccgc
          ||              || || || |||   | ||||       || ||||| ||||||
seq2     tag--------------ggaggacgctgc---gagtcatccatccccccctaagctccgc


         ->  <GABP>        -25      -GABP-
seq1     ccatcggaagcgccttctaagctcgttcttccg---ccagcttccctcCTCTTCCTTTCT
         |  | |  | |  ||||||| | ||||||||||   || || |  ||| ||| | || ||
seq2     ctctagcga-cttcttctaatcgcgttcttccggtcccggcctttctCTTCTCCATTCCT
                           -27

           -YY1--
seq1     CCGCCATCGTGGTG--TGTTCTTGACTCCGCTGCTCGCCATGgtaagtccatcggccccc
         |||||||||||     | ||||||| || ||| |||||||||||||| | ||||||| ||
seq2     CCGCCATCGTGCGCGCTATTCTTGATTCGGCTTCTCGCCATGgtaagactatcggccgcc
                                                                  ←-

                          +100
seq1     atcggccccgg---------g
         ||| | |||
seq2     atccgtccctcagcggctttc
         YY1>             +100
```

Note: alternative tsp's for human rpL39

seq1 = human rpL40
seq2 = mouse rpL40

61.72% identity.

```
      -200                    <Sp1--Sp1->        -GABP-          -150
seq1     ctcaag------tgactcggcgggcggggcccacaaccggaagcgggtgggcgaccttca
         |   ||      | ||||    |    |  ||||  ||||||||||         || ||
seq2     caggagctgctatcactcatgtgttaagctccac--ccggaagcgg---------ctcca


                                                    -100←-Sp1->
seq1     cccacgtgcgctgcggcttcgttcgccagcatccaagatggcggcagggcggggcccaag
           ||         || ||    ||||||| | |||| ||||| ||  ||||  ||||  |
seq2     gaca------gaacgactattctcgccaggacccaacatggc-gcccggcgaagcccggg


                               ATF/CREB
seq1     gcg----------cggcgcgaattgtgacgcaggcgtccggcgtgctccgt-cgcaagcg
         |||          ||||||| ||||||||| |  ||  | |||||||| || |||| |||
seq2     gcgggacgacatgcggcgcggattgtgacgtaaacgcgcagcgtgctcagtgcgcaggcg


                   -25                -GABP-
seq1     ctttcggcggcgattaggtggtttccggttccgctatCTTCTTTTTCTTCAGCGAGGCGG
         || || | || || |||| |||||||||||||||| || ||||| |||||| ||||||||
seq2     ctctcagaggagactagggggtttccggttccgctgtCCTCTTTCTCTTCAACGAGGCGG


seq1     CCGAGCTGGTTGgtggcggcggtcgtgcgg-------gttcgcgccgggccgagagcggg
         |||||| |||||||| || | |    ||||       ||| ||| | || |  | |||||
seq2     CCGAGCGGGTTGgtgccgtccgcgtcgcggctgggctgttggcggctgggcctgggcggg


                             +100
seq1     ttgggggctgcgggaggctgcagg
            | ||| | ||  |||
seq2     gacgcggcgggggtcggc------
                       +100
```

```
seq1 = human rpL41
seq2 = mouse rpL41

53.64% identity

      -200                                       <GABP>
seq1     ctgggggggaactggtaactcgaaaaccaaatactcggtcttccgaaagaactaactcaa
                   |||||         || ||  | || ||| ||      ||| |||  |||
seq2     ----------actgg---------aaacaggtgct-ggtgtt------gaaataatacaa
                -200


seq1     cctacccttctacaagagggt--------------------------------------cc
           || ||||  |  ||| ||                                      ||
seq2     --tatccttaaaatagacggaaaggtggggcttgataacacgcactctagtaaggcagcc
                          <GABP>                                     <

                                   -90---SP1--
seq1     gaaaaccactgttacgcccattgggtagccccgcccttggggggggcaaagggcgtgaaa
         | ||  | ||||        | | | |||||||||||  |||  | || | || |
seq2     ggaagtcgctgt-------ctcgagcagccccgccctaagggacgacacacgg-gccggc
         GABP>                   -90

          -GABP--ATF-   -40             -25
seq1     gcggaagtgacgacacccggcgctccattaaatagccgtagacggaacttcgcctttCTC
          ||||||||||||||||  || | |||||||||||| |||| |    | || ||   |||
seq2     tcggaagtgacgacaccgagcacgccattaaatagcagtaggc-ctccctctccgccCTC
            -50                        -25


seq1     TCGGCCTTAGCGCCATTTTTTTGGgtgagt------gttttttggttcctgcgttgggat
         |||| ||||||||||| ||  | ||| |||      ||||| ||      ||||| || |
seq2     TCGGTCTTAGCGCCATCTTCCTTGgttagtcgcggggttttctg------gcgttcgggt
                    <-YY1>

                                                 +100
seq1     tccgtgtacaatccatagacatctgacctcggcacttagcatc
         | |  || |||||| |  ||||| ||||||  || | |  |||
seq2     ttccagtgcaatccgttaacatccgacctccacattcactatc
                                                 +100
```

```
seq1 = human rpLP0
seq2 = mouse rpLP0

58.95% identity.

      -200                                      <GABP>
seq1     gcagcgccttccttcgcgaccctacttaaaggcggctttccgttg-------gctggagt
         || ||||| |||| ||      | ||       | |||| | ||        ||||||||
seq2     gc-gcgcc-tcctgcggccgggtgct-------gtctttactttcccgacttgctggagt
      -200

                                                     -100
seq1     ggcagcgatattatccaatggttgcctgtattcgttcagctttgtctgacgggcgatggc
         ||||||| | | | ||||| |  ||   | ||  || ||    |   ||| | ||||||
seq2     ggcagcggtctcagccaatagacgcgcatgtttattgagtgaggcaggacagtcgatgga


seq1     gc-agccaatagacaggagcgctatccgcggtttctgatt-----ggctactttgttcgc
          | ||||||||| || |  ||    |  ||  ||| | ||     | |  | | |||| |
seq2     accagccaataggcatggacg---acgtcgtcttcgggtttcgaggaccgcctggttctc

               -25
seq1     attataaaaggcacgcgcgggcgcgaggcccttCTCTCGCCAGGCGTCCTCGTGGAAGgt
           |||||||||||| |||||||  | ||||| ||||||||||||||||||||| | ||||
seq2     -ctataaaaggcacacgcgggcatgcggcccgtCTCTCGCCAGGCGTCCTCGTTGGAGgt
               -25

seq1     tcgtgtgctagttagatgggcgccaggggtcgccggcgggaagcatggaggggtctttgg
         | | | |  |  || | || |           |  ||||| ||| |  | ||| | |||
seq2     tggggggttgttttgttgtg----------ggagggcggcaag-agcgcggggacgttgc

                             +100
seq1     gggcct-----------ttgggaa
         |||              | |||||
seq2     gggtgggagcgatgggctcgggaa
                             +100
```

```
seq1 = human rpLP1
seq2 = mouse rpLP1

61.85% identity.

           -200
seq1     -----ttaacgttctc--------aggtcctataatttacccaaataaagtggaccgta
              ||||   ||||        ||  | | |||| | | |||         | ||  |
seq2     gcctgttaa---tctctactgttgagcacttgtaatattcgcaa---------gccccaa
      -200


seq1     tttgcaggcgctgagaaactttctcaacagtaagaaccaatcaggcgcag-----tgcgc
             ||  |||   | || |         | | |  | |||| ||  | |     |||
seq2     aggccaatcgcaccggaagt--------cgaacgcgcaaatccggaactgcgttctgcag
                      <GABP>

                                        -GABP-                    -50
seq1     aggcgccttcagtgggtcctgggtccgcctcttccgcccggtg-cgggcgccgattggtc
         | |||||| |  | |||  |||  | ||| |||||| |  | | |||||  |||||||||
seq2     acgcgcctcctctcggttttggccctgccccttccgtctcgagtcgggcatcgattggtc
                                                                   -50

                      -25
seq1     cgcgtggacacataagaggctgcgtataggcgagagccCCTTTCCTCAGCTGCCGCCA
          ||| || | ||||||| ||||||   |||||     |  | ||||||||||||||||||
seq2     ggcgcggtcccataagaagctgcgcgcaggcgtatatgatCTTTTCCTCAGCTGCCGCCA
                      -25

                +30
seq1     AGGTGCTCGGTCCTTCCGAGGAAGCTAAGGCTGCGTTGGGGTGAGGCCCTCACTTCATCC
         ||||||||||||||||||||||||||||||| ||||||||||||||||||||||||||||
seq2     AGGTGCTCGGTCCTTCCGAGGAAGCTAAGGCCGCGTTGGGGTGAGGCCCTCACTTCATCC
                          +40


seq1     GGCGACTAGCACCGCGTCCGGCAGCGCCAGCCCTACACTCGCCCGCGCCATGGCCTCTGT
         |||||||||||||| | |||||||        |||||           |||||| || ||
seq2     GGCGACTAGCACCGTG-CCGGCAG-------TCTACA-----------GCATGGCTTCCGT


              +149
seq1     CTCCGAGCT-------------------
         |||||||||
seq2     CTCCGAGCTCGCTTGCATCTACTCCGCC
                                 +150
```

seq1 = human rpLP2
seq2 = mouse rpLP2

55.42% identity.

```
      -200
seq1     ct---------------------------gcccttcgcctttgccgcgaaaagtgcctga
          |                           |||| | ||  ||     ||||| | |||
seq2     ttcgggagactctcgtccatgttgcagaggcccctggcactt-----gaaaaatccct--
      -200


seq1     cgg----gggctgggnaangggggagggggaaggagtcaaggacttccctccccgttggt
         ||     |   |||| ||     ||  ||||       || | ||||| ||
seq2     cgtttttgttgtggggaagacatgacaggga-------aatgtcttccttc---------
                                                        <GABP


seq1     cattggctgagcgcctcgtgacgtcactctgaggcg-cctcatgcggcgccgcgcacggg
               | | || || | |  ||||| |  |||||| || | |||| |||  || ||| |
seq2     ------cggtgcacc-cttttcgtcaatgggaggcgtccccgtgcgccgc--cgtacgcg
               ->

                                  -26--SP1--
seq1     tcccagagccttctgggtagcggtttaaccccgcctcttgcgtcggcgccttcCTTTTCC
         |||| | | |||||||||||||||||| |||||||   |||||| ||| ||||||||  |
seq2     tcccgggggcttctgggtagcggtttacccccgcccactgcgtcagcgtcttcCTTTCGC
                                         -20


seq1     TCCCTGTCGCCACCGAGGTCGCACGCGTGAGACTTCTCCGCCGCCTCCGCCGCAGACGCC
           || | |||| |||||||||||||||||||   | ||| ||||  |||    ||  |||
seq2     CGCCGGACGCCGCCGAGGTCGCACGCGTGAGGTCTGTCCCCCGCAGCCGAgtgagtagcc


                                      +100
seq1     GCCGCgtgagtgtggtgaccgggcccggggtg
             ||   | |  | || | |    ||||
seq2     tggccgcctgggccgcgaacagtggtggggac
                                      +100
```
