# Supplementary material for: The architecture of mammalian ribosomal protein promoters
Source: BMC Evol Biol. 2005 Feb 13;5:15. doi: 10.1186/1471-2148-5-15 (PMC554972; doi:10.1186/1471-2148-5-15)
Supplement: Additional File 4 — ST3: Characterization of the rp initiator. Occupancy matrix for determination of the consensus sequence of the mammalian rp initiator. [file 1471-2148-5-15-S4.pdf]

SUPPLEMENTARY TABLE 3

OCCUPANCY MATRIX FOR INITIATOR REGION OF MAMMALIAN RIBOSOMAL PROTEIN GENES

Numbers at each position represent the occurrence of designated nucleotide(s) or gaps in the aligned initiator sequences of 79 orthologous human and mouse rp genes. A, G, C, and T are exact matches. Y = C or T, R = A or G, W = A or T, K = G or T, S = C or G, M = C or A. Consensus sequence was determined according to standard convention.

Position	A	G	C	T	Y	R	W	K	S	M	Gaps	Consensus	% pyrimidine
– 8	7	13	14	11	9	11	0	2	5	4	3	N	45
– 7	4	12	22	8	11	6	1	5	4	2	4	N	55
– 6	2	12	25	14	7	3	2	3	4	2	4	N	61
– 5	5	11	17	15	13	4	0	3	4	4	3	N	59
– 4	5	8	37	5	10	0	1	1	6	3	3	N	68
– 3	0	13	11	29	9	4	0	5	2	2	4	N	65
– 2	1	8	10	34	10	1	2	3	6	2	2	Y	70
– 1	0	3	19	31	23	0	0	1	0	0	2	Y	95
+ 1	0	0	79	0	0	0	0	0	0	0	0	C	100
+ 2	0	0	16	55	7	0	0	0	0	0	1	T	100
+ 3	0	0	28	40	11	0	0	0	0	0	0	Y	100
+ 4	0	0	9	64	6	0	0	0	0	0	0	T	100
+ 5	1	0	20	51	7	0	0	0	0	0	0	T	99
+ 6	0	4	24	38	10	0	1	0	1	0	1	Y	92
+ 7	0	14	36	17	10	0	0	1	0	0	1	Y	81
+ 8	5	13	23	18	10	0	2	2	0	2	4	Y	68
+ 9	5	17	21	15	7	4	1	1	4	0	4	N	57
+ 10	4	14	27	20	3	3	0	1	1	3	3	N	66
