# Supplementary material for: The architecture of mammalian ribosomal protein promoters
Source: BMC Evol Biol. 2005 Feb 13;5:15. doi: 10.1186/1471-2148-5-15 (PMC554972; doi:10.1186/1471-2148-5-15)
Supplement: Additional File 5 — ST4: Promoter region sequences of non-rp TOP genes. Sources of non-rp TOP gene sequences, the amount of analyzed sequence 5' of the tsp, and location of insertion elements nearest the tsp. [file 1471-2148-5-15-S5.pdf]

SUPPLEMENTARY TABLE 4
NON RP TOP GENES ANALYZED

GENE	h-s	h-tsp	h-5'n	h-5'is	m-s	m-tsp	m-5'n	m-5'is
eEF1A1	U	*a*	1027	--	N		1002	129
eEF1B	U	*b*	1268	--	U	*b*	998	--
eEF2	N		999	311	N	*c*	997	475
PABP c1	N	*d*	378	96	U		287	264
hnRNPA1	U	*e*	1041	--	U		947	--
B23	N	*f*	1217	--	U		976	--
Tpt1	U		979	--	U	*g*	1000	--
gas5	N		1000	--	U	*h*	1002	600
U17HG	N	*i*	595	330	N	*i*	547	399

Determinations. of human (h-) or mouse (m-) tsp's:

a. Uetsuki, T., Naito, A., Nagata, S., and Kaziro, Y. 1989. Isolation and characterization of the human chromosomal gene for polypeptide chain elongation factor-1□. *J. Biol. Chem.* **264:** 5791-5798.

b. Chambers, D.M., Rouleau, G.A., and Abbott, C.M. 2001. Comparative genomic analysis of genes encoding translation elongation factor 1B□ in human and mouse shows EEF1B1 to be a recent retroposon event. *Genomics* **77:** 145-148 and DBTSS entries for NM_021121 (human) and NM_018796 (mouse).

c. Nakanishi, T., Kohno, K., Ishiura, M., Ohashi, H., and Uchida, T. 1988. Complete nucleotide sequence and characterization of the 5□-flanking region of mammalian elongation factor 2□gene. *J. Biol. Chem.* **263:** 6384-6391.

d. Hornstein, E., Git, A., Braunstein, I., Avni, D., and Meyuhas, O. 1999. The expression of poly(A)-binding protein gene is translationally regulated in a growth-dependent fashion through a 5□-terminal oligopyrimidine tract motif. *J. Biol. Chem.* **274:** 1708-1714.

e. Biamonti, G., Buvoli, M., Bassi, M.T., Morandi, C., Cobianchi, F., and Riva, S. 1989. Isolation of an active gene encoding human hnRNP protein A1. Evidence for alternative splicing. *J.□Mol. Biol.* **207:** 491-503.

f. Chan, P.K., Chan, F.Y., Morris, S.W., and Xie, Z. 1997. Isolation and characterization of the human nucleophosmin/B23 (NPM) gene: identification of the YY1 binding site at the 5□enhancer region. *Nucleic Acids Res.* **25:** 1225-1232.

g. Fiucci, G., Lespagnol, A., Stumptner-Cuvelette, P., Beaucourt, S., Duflaut, D., Susini, L., Amson, R., and Telerman, A. 2003. Genomic organization and expression of mouse *Tpt1* gene. Genomics **81:** 570-578.

h. Smith, C.M. and Steitz, J.A. 1998. Classification of *gas*5 as a multi-small-nucleolar-RNA (snoRNA) host gene and a member of the 5□-terminal oligopyrimidine gene family reveals common features of sno-RNA host genes. *Mol. Cell. Biol.* **18:** 6897-6909.

i. Pelczar, P. and Filipowicz, W. 1998. The host gene for intronic U17 small nucleolar RNAs in mammals has no protein-coding potential and is a member of the 5'-terminal oligopyrimidine gene family. *Mol. Cell. Biol.* **18:** 4509-4518.

h-5'n and m-5'n: the amount in bp of 5' flanking sequence analyzed in human and mouse genes, respectively.

h-5'is and m-5'is: the distance in bp from the tsp to the nearest 5' insertion element.
