# Supplementary material for: The architecture of mammalian ribosomal protein promoters
Source: BMC Evol Biol. 2005 Feb 13;5:15. doi: 10.1186/1471-2148-5-15 (PMC554972; doi:10.1186/1471-2148-5-15)
Supplement: Additional File 6 — Aligned promoter sequences of non-rp TOP genes. Annotated sequence alignments of 9 orthologous human and mouse non-rp TOP promoters. [file 1471-2148-5-15-S6.pdf]

```
seq1 = human eEF1A1
seq2 = mouse eEF1A1

44.15% identity.

      -300                                             ←-Sp1--→    ←-
seq1     cgtcgttaaactcccactaacgtagaacccagagatcgctgcgttcccgccccctcaccc

seq2     ------------------------------------------------------------

         Sp1>                                -200
seq1     gcccgctctcgtcatcactgaggtggagaagagcatgcgtgaggctccggtgccc-gtca
                              ||  ||| ||||||  |   | ||||     ||| |
seq2     --------------------ggggagagcagagcacac---aagctcatagaccctggtc


seq1     gtgggcagagcgcacatcgcccacagtccccgagaagttgggggga--------------
         |||||  ||| |      || ||      | |||  |  |||||||
seq2     gtggggggaggg------gcgca------ctgagcggggggggggannnnnnnnnnnnnn


seq1     ------------------------------------------------------------

seq2     nnnnnnnnnnnnnnnnnnnnnnnnnnnnnnnnnnnnnnnnnnnnnnnnnnnnnnnnnnnn


seq1     ----------------------------ggggtcggcaattgaaccggtgcctagagaag
                                     | | | ||     | |||||        | ||
seq2     nnnnnnnnnnnnnnnnnnnnnnnnnngggtgatgggggggaggaccgg--------ggag


                                                    -SP1--  -50
seq1     gtggcgcggggtaaactgggaaagtgatgtcgtgtactggctccgcctttttcccgaggg
          |||||||||| |||||||||||| | |||||||| ||||||||||| | ||||||||||
seq2     ctggcgcggggcaaactgggaaagcggtgtcgtgtgctggctccgccctcttcccgaggg
                                                            -50

                        -25
seq1     tgggggagaaccgtatataagtgcagtagtcgccgtgaacgttCTTTTTCGCAACGGGTT
         ||||||||||| |||||||||||| | ||||||| || ||||||||||||||||||||||
seq2     tgggggagaacggtatataagtgcggcagtcgccttggacgttCTTTTTCGCAACGGGTT
                        -25

seq1     TGCCGCCAGAACACAGgtaagtgccgtgtgtggttcccgcgggcctggcctctttacggg
         ||||| |||||| ||||| || | || |||||| | |||||||||  |||    |   ||
seq2     TGCCGTCAGAACGCAGgtgaggggcgggtgtggcttccgcgggcc--gccgagctggagg


                              +100
seq1     ttatggcccttgcgtgccttga--a
         |  ||  ||  ||| |||  |
seq2     tcctgctccgagcgggccgggcccc
                              +100
```

```
seq1 = human eEF1B
seq2 = mouse eEF1B

68.36% identity.

      -300  -GABP-
seq1     cg---gaagtggccccagcctcgaggccgggcgtcttcggtcatctccggcgcttctagg
         ||   ||||| |||   |||| | | ||| |||  |||| ||| ||||||| | ||   |
seq2     cgccggaagtagccgaggccttgcgtccgagcgctttcgttcaactccggctcctcagag
      -300


seq1     gctggttcccgtcatcttcgggagccgtggaggtacgaacttaagacatgcctattttat
         |  | ||||| || | |||||   || |||||  | |   ||   | ||||||  |||||
seq2     ggcgtttcccttcgt-ttcggagtccttggagcgaaggcattttaaaatgcctcctttat


seq1     taatttacttccaaacgcaacgaaaggtccatggacaatttgtgggccatttaattcagg
         |||||| ||||||||  ||| | |||| |   |||| |  |||||||| |||| ||
seq2     taattttcttccaaagacaagggaaggccggcggacgacctgtgggccctttagtt----


seq1     gcccccaattcg------tacgtggagaagtgggaatgcaaaagtactttgacctttaac
             |||| |||      |||| |||| |  | || |   || | || ||||||||||||
seq2     ---accaagtcgcagtggtacgcggaggaccgagactaggaacggaccttgacctttaac

                 --Sp1--                          -25      -GABP-
seq1     cttcggtccggcgcggtggagggaaacgcctccgtctctatataaggaattttccggtct
         | ||   |||| |||| ||||||||||||||||||||||||||||||| |||||||| |
seq2     c-tctagccggggcggcggagggaaacgcctccgtctctatataaggagttttccggacg


seq1     cttcgggtcCTTTTTCCTCTCTTCAGCGTGGGGCGCCCACAATTTGCGCGCTCTCTTTCT
         ||  |||||||||||  |  | ||||||  || |||   |     ||||  |||    ||
seq2     ctctgggtcCTTTTT--TTCCCTCAGCGCTGGCCGCGGCCTCCCGGCGCTCTCGTCCTCC

                                                      +100
seq1     GCTGCTCCCCAGCTCTCGGATACAGCCGACACCATGGGTTTCGGAGACC--
         |||  | | |||||| | | |||||  | ||||||| |||||||||||
seq2     GCTTTTGCACAGCTCCCCGTTCCAGCCTTCGCCATGGGATTCGGAGACCTG
                                                        +100
```

```
seq1 = human eEF2
seq2 = mouse eEF2

55.32% identity.

      -300
seq1     ggttttaatagaagtactgga--caacttgtaagctaatatcgttgctatggttctcgtt
          |   ||  ||   | |  ||  ||  |||||||| | | |                |||
seq2     tgacctacaaggttttcaagaatcatgttgtaagcaactgt----------------gtt
      -300

seq1     ctcagc----------taaaa-------cggcgctctttactttgtgcac-----ctgaa
         || ||           |||||       ||  | ||||  |   | |  |     ||||
seq2     ctgaggaatctatgtttaaaaacccatccgtggatcttggcccagggtccagagactgag

                                      ←SP1--→                    -150
seq1     cactgcacaccgagggcg------accaccgcccccgagatgcccagcttctattctaga
         |    ||| ||  || ||      | |  | ||| ||  |   | ||  |  | ||   |
seq2     ctagccacgccccggccgcgccgcagccactcccacggcagttcaagtgttaagtcccaa


                                      ←-SP1---→        -100
seq1     gcgccgcgccggcgccgaatgggttaacgggcggggggacacgcctccgtgcgcttgcgc
            ||||||          |  ||  | | ||     ||| || |  |    |  | |
seq2     agaccgcgc----------tctgtgcatgcgc----agacccgtccacagctggctccta


                     ←SP1-→         -60         ←SP1-→
seq1     ggcgtcccttcgccccgccttcgcagcgcagtcacatgacccgcccaaccggcgtccgcc
         | |  |||  |    ||    | | |||| ||||| |||| | |||||||||||||  ||
seq2     gccaacccggccggacgagcacccggcgccgtcacgtgacgcacccaaccggcgtcgacc


           -25                                            --YY1--   +30
seq1     tataaaaagctgagtgttgacgtcagcgttCTCTTCCGCCGTCGTCGCCGCCATCCTCGG
         ||||||| || | | ||||||||||||| ||||||||||||  | |||||||||| ||||
seq2     tataaaaggccgggcgttgacgtcagcggtCTCTTCCGCCGCAGCCGCCGCCATCGTCGG
           -25                                                      +30

                                                 --YY1--  +80
seq1     CGCGACTCGCTTCTTTCGGTTCTACCTGGGAGAATCCACCGCCATCCGCCACCATGgtga
         ||||  || ||   |||   |||  ||  ||||||||  |||||||||||||||||||||
seq2     CGCGCTTCCCT--GTTCACCTCTGACTCTGAGAATCCGTCGCCATCCGCCACCATGgtga
                                                           +80

                 +100
seq1     gtaaccctg--c
         ||  ||  |
seq2     gtctcctcggct
                 +100
```

seq1 = human PABP
seq2 = mouse PABP

65.73% identity.

```
seq1      ---------------------------ggggactcagca-----------acacccccga
                                     ||| | || ||               |  |  |
seq2      tccctctctttcctggctttgcgcagcgggaagtcggcccctggctgtggcgcctgcgca
       -250

             <-SP1>         -250
seq1      ggaccgcccgcgagccgtcctccccacccccacaccgcagcctcca----ccgccgccac
          | | | ||  |  |||     ||| |||||||| ||     |  |    ||||||||||
seq2      gaagccccgccccgcctcgggcccaacccccacgcccgcagcagcggcagccgccgccac
              ←-SP1--→                         -150

seq1      ctctcccttcctctctgctctttcctcctgtt--ttctcttccctcctccccctggcctc
          ||||||||||||||  || |||||||||   |  | |    ||  ||
seq2      ctctcccttcctct--gcgctttcctccgcctcctccggcgccgtc--------------

                                                              -60
seq1      cgcgtctcctcctactccggcgctgacgctcgcgtaggggccctggcgtcagacgcgcgg
                                     ||||||||| ||||||||||||||||||||||
seq2      ---------------------------gctcgcgta-gggccctggcgtcagacgcgcgt
                                                              -60

          ---SP1--                   -25               ---SP1---
seq1      gggcggggcgagtgcggcgcggggtataagtagagggtgcaggaggcggtgcttCCCCTT
          ||||||||||||||||||| |||||||||||||||||||||||||||||||||||||||||
seq2      gggcggggcgagtgcggcgggggggtataagtagagggtgcaggaggcggtgcttCCCCTT
                         ←-SP1--→    -25

seq1      CTCCCCGGCGGTTAGTGCTGAGAGTGCGGAGTGTGTGCTCCGGGCTCGGAACACACATTT
          ||||||||||||||||||||||||||||||||||||||||||||||||||||||||||||
seq2      CTCCCCGGCGGTTAGTGCTGAGAGTGCGGAGTGTGTGCTCCGGGCTCGGAACACACATTT

                                        +100
seq1      ATTATTAAAAAATCCAAAAAAAATCTAAAAAAAT
          ||||||||||||||||||||||||||||||||
seq2      ATTATTAAAAAATCCAAAAAAAATCTAAAAAATC
                                        +100
```

```
seq1 = human hnRNPA1
seq2 = mouse hnRNPA1

71.14% identity.

      -300
seq1     aaaaagatgttaaacacccaaggtagattcaaatgtgaatgattggtcggttggccaatc
         |||   || | ||| ||   |  || |||||||| | | ||| |||   ||| |||||||
seq2     aaaggcatttaaaagacgtga-atacattcaaatataagtgactggctagttagccaatc
      -300


seq1     agactggttaacaataacattactcgggaaccaatggactccaaggggtggagacggcgt
         |    | ||||  |||| ||  || |||     |  ||||| |||||| |||| | |  |
seq2     aatgcgattaaggataagatcccttggggcatcagtgactcaaaggggcggagtctgaat
                                               -200   ←SP1-→

seq1     agaacgaccgaaggaatgacgttacacagcaatgtggcaccacaggccaatagcaggggg
         |||||| || || | ||| |||| |  ||||  | | |  ||| ||||||| |  || ||
seq2     agaacgcccaaacggatgccgtttcttagcagggcgtcttcacgggccaatggtgggagg


                                           -SP1--       -70
seq1     aagcgatttcaagtatccaatcagagctgttccagggcggagtctaccaatgccgaaagc
          |   ||||||| | |||||| |||        |||||||||||||||||| |||| |||
seq2     gaataatttcaaatctccaataagactcaagtaagggcggagtctaccaataccgagagc


              --SP1--     -40                -25
seq1     gaggaggcggggtaaaaaagagagggcgaaggtaggctggcagatacgttcgtcagcttg
         ||||||||||| |||||  | || |  |||||||||||||| |  |||||||| | | | 
seq2     gaggaggcgggataaaagggcga-gcagaaggtaggctggcgggcacgttcgttatcgta
                                                ←SP1→


seq1     ctCCTTTCTGCCCGTGGACGCCGCCGAAGAAGCATCGTTAAAGTCTCTCTTCACCCTGCC
          |||||||||| | | ||||| ||||| ||||||||| | ||| ||||| | || || ||
seq2     ttCCTTTCTGCTCTTTGACGCTGCCGAGGAAGCATCGCTGAAGGCTCTCGTAACTCTACC


                                                +100
seq1     GTCATGTCTAAGTCAGAGgtgagttaggcgcgctttcc--ca
         |||||||||||||| ||||| |||| |  ||||||| |  | 
seq2     GTCATGTCTAAGTCCGAGgtaagttggatgcgctttgcagct
                                                +100
```

seq1 = human B23
seq2 = mouse B23

72.79% identity.

```
      -304
seq1     gggagaggacgtggaagggttggaggtggggagtgcgcgttacgactggaaagcacgcgt
          ||||| ||||||||||   || |      ||| | | |  || |   |||| | ||| |
seq2     -ggagaagacgtggaagccctgaaaacccagagcgag-gccacaaagagaaaacgcgcct
       -304


seq1     gcgcacaggcgacagcagcggaggggtggggccagtgtacgagtgcgcgtgctcggtggg
         ||||   |||||||| || ||||||  |||||||| | ||||  |||||||| | ||| |
seq2     gcgcttgggcgacagtagtggagggacggggccagag-acgaacgcgcgtgcgcagtgag


                                    ---SP1---     -140
seq1     agcccgcggagtacctggaaggaggtgggggcgaggtagaaaggagtggggttgaaaagc
         |||||||  || ||||||| |  |  ||||||||||||   ||| ||| || ||||| ||
seq2     agcccgccaagcacctggaggaggaggggggcgaggtacctaggcgtgaggatgaaaggc
           ←SP1→                                     -140

                                                                    <-
seq1     -cttgcgcaggacggctacggtacgggg-gtgggagggcttcggagcacgcgcgcggagg
          |  |||||  |  || || || ||||| ||||||||| | | ||| | || ||||||||
seq2     gcacgcgcaaaagcgcgactgtgcggggagtgggagggatccagaggaggctcgcggagg

         SP1>            --BOXA--               -25
seq1     cgggacttgggaagcgctcgcgagatcttcagggtctatatataagcgcgg-gagcctgc
         | ||||| | | ||  |||||||||||| | | | | ||||||| | |||| ||||| ||
seq2     caggactagcgcagatctcgcgagatctcctgcgaccatatatacgtgcggagagcccgc
                                                  -25


seq1     gtcctttCCCTGGTGTGATTCCGTCCTGCGCGGTTGTTCTCTGGAGCAGC-----GTTCT
         ||||||||| ||| ||||||||||||||||||  |||||| |||| |||      ||| |
seq2     gtcctttCCTTGGCGTGATTCCGTCCTGCGCGTCTGTTCTGTGGAACAGGAGGCAGTTGT


                                                       +96
seq1     TTTATCTCCGTCCGCCTTCTCTCCTACCTAAGTGCGTGCCGCCACCCG
         ||    |||||||| |||||| |  ||| ||||||| ||| ||| ||
seq2     TT----TCCGTCCGGCTTCTCCCACACCGAAGTGCGCGCCTCCA-CCT
                                                       +96
```

```
seq1 = human Tpt1 (P23)
seq2 = mouse Tpt1 (P23)

66.74% identity.

      -300
seq1     tccgcttcccccg-------ctccacccacccagggctagggagcgccccgagag--ttg
         |   |   ||| |       ||| | |  ||  || |   ||   ||| | ||    ||
seq2     tggccacgcccgggcggcgactcaagcgtcc--ggccatcggtcggccgcaagtcccttc
      -300          ←Sp1→

                                     ←--Sp1--→         -200
seq1     gcctcctcccacgctgcgcgcgcacctccccgcccccacccctacccgctggcgtgccca
          | |||   || ||  || ||||||   ||   | || ||||      |  |||  ||||
seq2     ccgtcccagcatgccccgggcgcactatccgcacaccgcccccgttgccccgcgcaccca
                                             ←Sp1--→

seq1     gtggaacggagccttgtgtctccgcctcaagtccccggatgctcacctccccgactcgcc
         | || ||   || ||||||| | ||    |||||||||||||   |||||| |   || |
seq2     ggggcactccgcattgtgtcccagc--agagtccccggatgc---cctcccggggccggc

                   ----Sp1---      -100                          <GABP
seq1     cccgctgtggccccgcccccgcgcggctcttcgtgccacgtcaccgcctgcgtcgcttcc
         |  || || ||| ||||||||| | || || |||  |||||||||||   || |  ||||
seq2     cgggc-gtagccacgcccccgcaccgccctgcgt-tcacgtcaccgctgacgacagttcc
                                 ←Sp1->

         >                                         -25
seq1     ggaggcgcagcgggcgatgacgtagagggacgtgccctctatatgaggttggggagcggc
         || || || |||| || ||||||||  |  |||||||||||||||||||||||||||| |
seq2     gggggagccgcggacggtgacgtagccgagcgtgccctctatatgaggttggggagcgcc

seq1     tgagtcggcCTTTTCCGCCCGCTCCCCCCTCCCCCCGAGCGCCGCTCCGGCTGCACCGCG
          | |||||||||||||||||||||||||||||||||| ||||||||||||||||||||||
seq2     cgcgtcggcCTTTTCCGCCCGCTCCCCCCTCCCCCCGCGCGCCGCTCCGGCTGCACCGCG

                                                                 +100
seq1     CTCGCTCCGAGTTTCAGGCTCGTGCT---AAGCTAGCGCCGTCGTC-------GTCTCC-
         |||||| |            || |||   | |||||||||| ||||       ||| ||
seq2     CTCGCTTC------------CGCGCTGTCAGGCTAGCGCCGCCGTCCCCAGCCGTCACCA

seq1     -

seq2     T
      +100
```

seq1 = human gas5
seq2 = mouse gas5

55.14% identity.

```
       -300
seq1      ccc-------------------ccccttcccccatccccagagctttccttgcctcgcgc
          ||                    | ||   ||||| |||||| |||||||||| |  | ||
seq2      ccggaaggaaatcagtcaccctcaccccgccccacccccagcgctttccttg-caggagc
           <GABP>                 ←-SP1--→

                                                --SP1--          -200
seq1      cccctcccctctctgctcttcctcctcagtcgggaggagggcgggagcacggcatcacgt
          ||||||||  ||| ||   | ||||||||   || ||||||||       ||| |  |||
seq2      cccctccc--ctcagcctgtactcctcagggaggcggagggcg-------ggcctatcgt
                                                  -200

                                              ←-SP1-→    ←-SP1→   -140
seq1      ggacg-gtcatgtctctgcccacaatggcgaggaagggcggggaaaaggggcggacttaa
          |  || | ||   ||| |||| |   |||      ||||| ||  |  |||     ||
seq2      gcccgcggca--actccgccctc-cgggctctcgggggcgtggccagagggaaagttttg
                         ←SP1→                                  -140

seq1      ggggtgggaagaaggtgatactggatgagaggaagattgagggaggttagggggaggagc
           |||   ||||||||||   ||   ||||  | ||  |||||| |  |  |||| || |
seq2      tgggcctgaagaaggtggggct---tgaggaggagtctgagggcgtgtgaggggcggggt
                                                             ←-SP1--→

                                                              -25
seq1      cgaagcggacgctgccgggagcgt-gcgtcgtgacgtcatcaaaagaactcttatataca
            ||| |||||| ||  | ||| | ||||| ||||||||  || |     |||||||||
seq2      ttaagtggacgcagcaagcagctttgcgtcttgacgtcacgaaga----ccttatatact
                                                               -25

seq1      ggagcccaggcaccata-ctgtCTTTTCGAGGTAGGAGTCGACTCCTGTGAGgtatgttt
           || | ||| | ||||  | | ||||  ||| |  | | | | ||     |||||||
seq2      cga-ctcagccgccatctcagCCTTTCGGAGCTGTGCGGC-ATTCTGAGCAGgtatgcaa

seq1      tatctttgcgaatgttgcgggttttggggcgctccagcctttgtctgctaaggtcaccct
          | ||  || | |||||  |           ||| | | |||   ||| | |   | ||
seq2      tttc-ctgagtatgttatg-----------gcttcgggccttggaggctgaag---cact

                       +100
seq1      g---------------a
          |
seq2      ggaagctggtgggattc
                       +100
```

seq1 = human U17HG
seq2 = mouse U17HG

43.21% identity.

```
      -300
seq1     agggcatatcctacagactgagtccagtgattgcacagaagtaaacgtcctctgcagcta
                     ||| ||  |||             |||   |||    ||||| |
seq2     ------------acaaacaaagt-------------agattcaaa----ctctgga----


seq1     catacctacaaacctatttctgtaacgta--------------------cattccccagc
            | ||| |||  ||||||| ||   |                    |||   ||||
seq2     ---atctagaaa-atatttctctaggctttttttttttttttcattttgcatataccag-


seq1     aaggtcccgcgggaaggatccactaccgcgagagg-----cctcccagccaggaaggggt
                        | ||||   |   |||||||     |||| ||  |   |     |
seq2     --------------tgtatcc---aggccgagaggacaaacctcacaagctcaacaattt


seq1     ggggctcaatctgc---------agtagatt-------------cccagaagcctcagtg
            ||| |  | ||         |||||  |             ||||  ||||| || |
seq2     cacgctaagaccgccaacaccttagtagggtaaaagttctcgatcccatcagccttagcg

                                                                -60
seq1     agtttctgattct-ctaactgcgcatgcttctgcgcacgcgcaa------tagacattcc
           ||||  || || |||| || || |    ||||||| |||||       | |||    |
seq2     catttccaatgcttctaagtgagcgtaaccctgcgcaggcgcaggcatagtcgaccacgc
                                                                 -60

             -GABP-                       -25
seq1     aggacttccgggcacttcgtaaggtttaaaaa-ggatgcttcgcgttttctctctCCTTT
           |||||||||||  | | |||||| |||| | || ||| |||       ||| ||  |
seq2     ctgacttccgggcgttacttaaggtataaatatggctgcgtcggcgcgggtctgtCTCTC
                                   -25


seq1     TTGGAGACAGATTCGCAGTGGTCGCTTCTTCTCCTTGgtaagtgtgatccttggtaagtg
         | || | |            | || |||  | | ||||          | |||||| |||
seq2     TAGGCGTC-----------GCTCTCTTGGTGTGCTTG----------TTCTTGgtgagta


                                                                    +100
seq1     tgatcagatgcttgccaccggagttgtgggtcta------------------------a
            |  | ||||   | | |  | || |||| |
seq2     cagtgcgctgct---ctcggctgctgagggtttgcccgcgcggtcccgcacgggctccg
                                                                    +100
```
