# Supplementary material for: The architecture of mammalian ribosomal protein promoters
Source: BMC Evol Biol. 2005 Feb 13;5:15. doi: 10.1186/1471-2148-5-15 (PMC554972; doi:10.1186/1471-2148-5-15)
Supplement: Additional File 7 — ST5: Housekeeping gene promoters. List of 20 housekeeping gene sequences extracted from the Eukaryotic Promoter Database, which were analyzed for GABP, Sp1 and YY1 binding sites and for TATA box quality. [file 1471-2148-5-15-S7.pdf]

SUPPLEMENTARY TABLE 5
HOUSEKEEPING GENE PROMOTERS

**GENE**	**EPD NUMBER ep--**
Hs superoxide dismutase 1 (*SOD-1*)	07053
Hs dihydrofolate reductase (*DHFR*)	07056
Mm hypoxnthine phosphoribosyltransferase (*HPRT*)	07058
Hs adenosine deaminase (*ADA*)	11113
Mm thymidylate synthetase (TS)	15031
Mm adenosine deaminase (*ADA*)	15032
Mm DNA polymerase b'	16049
Mm dihydrofolate reductase P1 (*DHFR*)	24032
Hs thymidine kinase (*TK*)	25035
Mm RNA polymerase II (*RP215*)	28001
Hs glucose-6-phosphate dehydrogenase (*G6PD*)	30014
Mm malate dehydrogenase cytosolic (MDH1)	33012
Hs nucleolin	36018
Hs ADP-ribosylation factor 1 (ARF1)	39001
Mm p97 ATPase	59010
Hs cyclin D1	60011
Hs DNA nucleotidylexotransferase (*TdT*)	64003
Hs topoisomerase 3 alpha (*TOP3A*)	64007
Hs single stranded DNA binding protein 1	73019
Hs glyceraldehyde-3-phosphate dehydrogenase (*GAPD*)	73651

The – 200 to +100 regions of these promoters were scanned for potential GABP, Sp1, YY1 and BoxA factor binding sites (as defined for nonaligned motifs in Figure 2).
